# Temporal stability and community assembly mechanisms in healthy broiler cecum

**DOI:** 10.3389/fmicb.2023.1197838

**Published:** 2023-09-13

**Authors:** Aqsa Ameer, Youqi Cheng, Farrukh Saleem, Uzma, Aaron McKenna, Anne Richmond, Ozan Gundogdu, William T. Sloan, Sundus Javed, Umer Zeeshan Ijaz

**Affiliations:** ^1^Department of Biosciences, COMSATS University, Islamabad, Pakistan; ^2^Water and Environment Research Group, Mazumdar-Shaw Advanced Research Centre, University of Glasgow, Glasgow, United Kingdom; ^3^Moy Park, Armagh, United Kingdom; ^4^Department of Infection Biology, Faculty of Infectious and Tropical Diseases, London School of Hygiene and Tropical Medicine, London, United Kingdom; ^5^Department of Molecular and Clinical Cancer Medicine, University of Liverpool, Liverpool, United Kingdom; ^6^College of Science and Engineering, University of Galway, Galway, Ireland

**Keywords:** *16SrRNA* gene sequencing, microbiota, stability, assembly, broiler

## Abstract

In recent years, there has been an unprecedented advancement in *in situ* analytical approaches that contribute to the mechanistic understanding of microbial communities by explicitly incorporating ecology and studying their assembly. In this study, we have analyzed the temporal profiles of the healthy broiler cecal microbiome from day 3 to day 35 to recover the stable and varying components of microbial communities. During this period, the broilers were fed three different diets chronologically, and therefore, we have recovered signature microbial species that dominate during each dietary regime. Since broilers were raised in multiple pens, we have also parameterized these as an environmental condition to explore microbial niches and their overlap. All of these analyses were performed in view of different parameters such as body weight (*BW-mean*), feed intake (*FI*), feed conversion ratio (*FCR*), and age (*days*) to link them to a subset of microbes that these parameters have a bearing upon. We found that gut microbial communities exhibited strong and statistically significant specificity for several environmental variables. Through regression models, genera that positively/negatively correlate with the bird’s age were identified. Some short-chain fatty acids (SCFAs)-producing bacteria, including *Izemoplasmatales*, *Gastranaerophilales*, and *Roseburia*, have a positive correlation with age. Certain pathogens, such as *Escherichia-Shigella*, *Sporomusa*, *Campylobacter*, and *Enterococcus*, negatively correlated with the bird’s age, which indicated a high disease risk in the initial days. Moreover, the majority of pathways involved in amino acid biosynthesis were also positively correlated with the bird’s age. Some probiotic genera associated with improved performance included *Oscillospirales*; *UCG-010*, *Shuttleworthia*, *Bifidobacterium*, and *Butyricicoccaceae*; *UCG-009*. In general, predicted antimicrobial resistance genes (piARGs) contributed at a stable level, but there was a slight increase in abundance when the diet was changed. To the best of the authors’ knowledge, this is one of the first studies looking at the stability, complexity, and ecology of natural broiler microbiota development in a temporal setting.

## 1. Introduction

The challenge of global food scarcity is paramount, and efforts have been directed at agro-based sectors toward high generative capacities with the minimum possible resource utilization. In this respect, the poultry industry, particularly the broiler farming sector, has been playing a pivotal role for decades, with annual growth of 5% (FAO, 2017). Broilers are an important food source, specifically due to their cheap and high-quality protein content (El-Deek et al., 2020). Bird feed conversion efficiency is actuated by gastrointestinal microbial species (Kogut, 2019; Sztandarski et al., 2022). Over 900 bacterial species have been explored so far in the chicken gut, comprising commensals and pathogens (Ranjitkar et al., 2016; Binek et al., 2017; Fathima et al., 2022).

The advantageous bacterial communities are involved in chicken feed digestion, nutrient assimilation and absorption, pathogen proscription, and immunity development, contributing to disease resistance (Stanley et al., 2012; Park et al., 2017; Borda-Molina et al., 2018). The commensal microbiota have an evident antagonistic effect on pathogenic bacteria by way of colonization conflict, immune intonation, and production of antimicrobial molecules such as organic acids, bacteriocin, and hydrogen peroxide (Yeoman et al., 2012; Kim et al., 2015). This phenomenon of competitive exclusion, first coined by Nurmi, is illustrated by the decreased relative load of *Helicobacter* and *Campylobacter* by increasing the relative load of certain other (probiotic) genera (Nurmi and Rantala, 1973; Kaakoush et al., 2014).

The chicken cecal microbial environment is dominated by a high abundance of unclassified bacteria and bacterial low abundance reads at both the genus and species levels at the early stages of development (Proszkowiec-Weglarz et al., 2022). These bacteria are mainly acquired through the passage of eggs via the mother hen’s reproductive tract (Lee et al., 2019). Newly hatched chicks are usually exposed to environmental and non-avian sources of bacteria; therefore, the gut colonization pattern is highly variable and characterized by low diversity and high instability that is governed by several environmental and host-associated factors (McKenna et al., 2020; Fathima et al., 2022; Proszkowiec-Weglarz et al., 2022). The highest microbial diversity has been observed in cecal content, holding up to 10^11^ organisms per gram (Huang et al., 2018). Chicken gut development in commercial production systems is relatively specific, as chicks never come into contact with adult birds (Polansky et al., 2016).

Recently, the diversity of gut bacterial communities in broiler birds was examined, and it was found that 90% of ASVs belonged to *Firmicutes* and *Proteobacteria* (Li et al., 2022). The microbial community’s complexity increased with age throughout the productive lifespan, with maximum stability during the 3rd week and instability during the 6th week (Yan et al., 2017; Huang et al., 2018; Feye et al., 2020). Previously, it has been reported that colonization and replacement of certain microbial communities with more stable taxa occurs as broiler age advances (Lu et al., 2003; Mohd Shaufi et al., 2015). In general, age is considered an important factor influencing the gut community structure, function, and diversity, with initial colonizers as facultative anaerobes followed by strict anaerobes (Zhu et al., 2002; Palmer et al., 2007; Oakley and Kogut, 2016).

In our previous study (Ijaz et al., 2018), an in-depth sampling regime was used to assess daily changes in microbiota. The study highlighted how alpha diversity increases rapidly during the initial 12 days, as well as how beta diversity converges to a stable solution. More importantly, phylogenetic alpha diversity measures such as the net relatedness index (NRI) and the nearest taxon index (NTI) showed a step response (days 12–20), hypothesizing a window of opportunity where a pathogen such as *Campylobacter* may appear. However, beyond diversity estimates, we did not explore the spatial (pens) and temporal dynamics and how the structure of microbial communities can reveal information about the stability of ecosystems. In the past few years, there has been a reinvigorated interest in microbial ecology, particularly in view of advancements in null modeling techniques. Therefore, to consolidate these new analytical approaches, we are revisiting them (Ijaz et al., 2018) to explore important concepts such as *microbial niche differentiation* (Verster and Borenstein, 2018). There are several hypotheses in circulation on how species occupy a certain niche (Granot et al., 2017), namely, the *early succession hypothesis*: early successional species are eventually responsible for occupying a niche; the *abundance hypothesis*: abundant species are more likely to occupy a niche; and the *niche-breadth hypothesis*: generalist species are more likely to thrive as they are capable of surviving under a wide range of conditions. In our original study, the microbial community data originated from 12 pens (each with their own environmental properties), which makes it a perfect candidate to explore concepts such as niche breadth and overlap as well as taxon-environment relationships in a recently developed null modeling-based MicroNiche framework (Finn et al., 2020).

To identify transient microbial taxa at very narrow ranges of a parameter of interest or as a result of disturbances on a temporal scale, there is an emphasis on *conditionally rare taxa* that have a bimodal response in terms of abundance (Lynch and Neufeld, 2015). These rare taxa provide a reservoir of functions and offer a means toward achieving resilience. Beyond this approach, we apply a null modeling-based *specificity* measure by Darcy et al. (2022) that is able to pick out taxa becoming active at narrow ranges. Our original study (Ijaz et al., 2018) has three diets, *starter* (days 0–10), *grower* (days 11–25), and *finisher* (days 26–35). We hypothesize that the introduction of individual diets should shift the microbiome by either the proliferation or disappearance of species and by making some microbes specific. This would also offer additional insights into microbial community function linked to dietary or environmental changes.

While the above approaches identify individual taxa, we are also interested in a minimal subset of taxa that form functional ecological groups based on statistical patterns. There is reasonable evidence to suggest that simplistic structure in community-level function and coarse-grained taxonomic groups arise as a result of nutrient availability (Goldford et al., 2018). These taxonomic or functional guilds assembled as a result of some outcome predictors have recently been studied as an *ensemble quotient optimization* problem (Shan et al., 2022), through which an “*ensemble*” (a minimal subset of the microbial community associated with a categorical or continuous outcome) can be extracted.

Working beyond understanding single species and their sub-composition, it is also important to understand top-level microbial community or ecosystem functioning. The recently developed method by Yonatan incorporates interacting traits such as *commensalism*, *amensalism*, *competition*, *mutualism*, and *predator*–*prey* in stability criteria (Allesina and Tang, 2012) without the need to generate co-occurrence patterns (Yonatan et al., 2022), as opposed to the traditional methods, to suggest the resilience of microbial communities against outside influences or disturbances.

The aim of this study is to explore the stability and assembly patterns of chicken cecum microbiota at diverse levels (coarse-grained to fine-grained) by revisiting the dataset of Ijaz et al. (2018) through the aforementioned novel analytical approaches. Some of these incorporate underlying ecology and environment (as discussed above); others, such as the generalized linear latent variable model (GLLVM) approach (Niku et al., 2019), were not available at the time of original publication and offer better predictive modeling for the metadata. Furthermore, with recent advancements in predictive metabolic modeling tools such as PICRUSt2 (Douglas et al., 2020), by virtue of an updated and larger database of gene families and functions, we are now better able to explore functional dynamics. Therefore, we have also explored the temporal dynamics of functional pathways and enzymes, including those related to antimicrobial resistance.

## 2. Materials and methods

A detailed description of the experimental design, sample collection, bird’s performance measurements, and sequencing is provided in the previous study (Ijaz et al., 2018). Briefly, the study comprises cecum microbiome samples of 396 broiler (Ross-308) that were allocated to 12 pens (33 broiler chicks/pen) and were fed with a three-phase diet, i.e., *starter diet* (up until day 10), a *grower diet* (from day 11 to day 25), and a *finisher diet* (from day 26 to day 35). One cecal sample was collected daily from each pen (12 samples/day), leading to 396 samples in total, out of which a total of 17 were removed from the final analysis due to poor gDNA quality, giving a final *n* = 379 samples, which were subjected to *16S rRNA* amplicon sequencing using a V3–V4 primer set on an Illumina MiSeq, giving 300 bp paired-end reads.

### 2.1. Bioinformatics and statistical analysis

**Figure 1:**
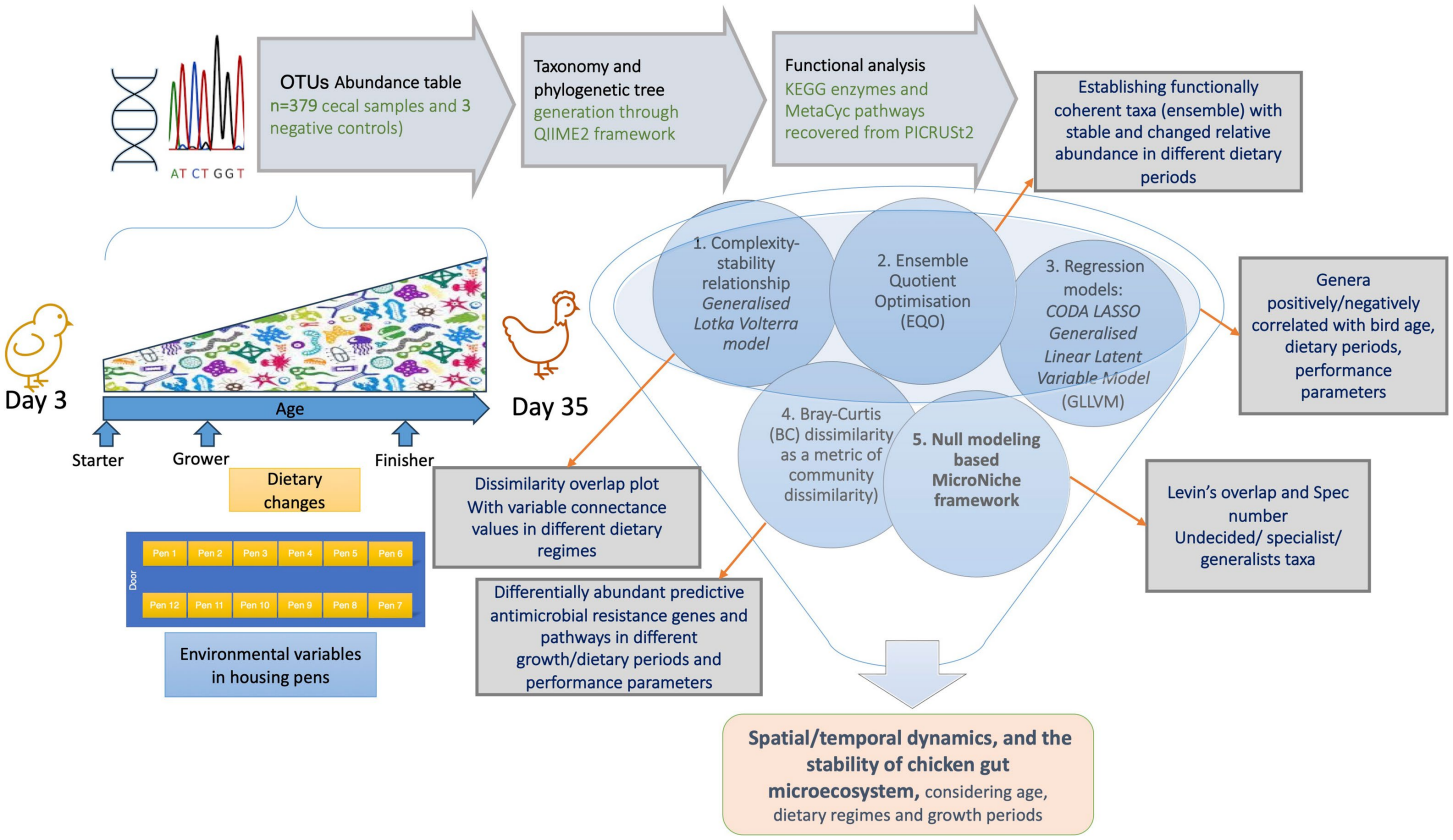
Schematic of the methodology used in this study with the details given in the Supplementary material.

We have used the abundance table (*p* = 18,588 OTUs for *n* = 382 samples that include additional negative controls) and representative operational taxonomic units (OTUs) from Ijaz et al. (2018) with two modifications: (a) we re-classified the taxonomy of the OTUs using the recent SILVA SSU Ref NR database release v.138 (Quast et al., 2012), and (b) we re-generated the rooted phylogenetic tree with the QIIME2 framework (Caporaso et al., 2010). Furthermore, we used PICRUSt2 (Douglas et al., 2020) within the QIIME environment to recover KEGG enzymes and MetaCyc pathway predictions (not done in the original publication) for all the samples. For this purpose, we used the parameters --p-hsp-method pic --p-max-nsti 2 in qiime picrust2 full-pipeline.^1^ QIIME2 was also used to generate a final BIOM file that combined abundance information with the new taxonomy, along with the new phylogenetic tree, and the metadata were used for the downstream statistical analysis. The metadata comprised performance parameters related to each broiler including mean body weight (*BW_mean*), body weight gain (*gain*), feed intake (*FI*), and feed conversion ratio (*FCR*). These measurements were taken at time points 3–7 days, 8–14 days, 15–24 days, and 25–35 days. Statistical analysis was performed using the R software (R Core Team, 2013), with the methodology summarized in Figure 1 and the details provided in the Supplementary material.

## 3. Results

### 3.1. Overall complexity-stability patterns at a temporal scale

**Figure 2:**
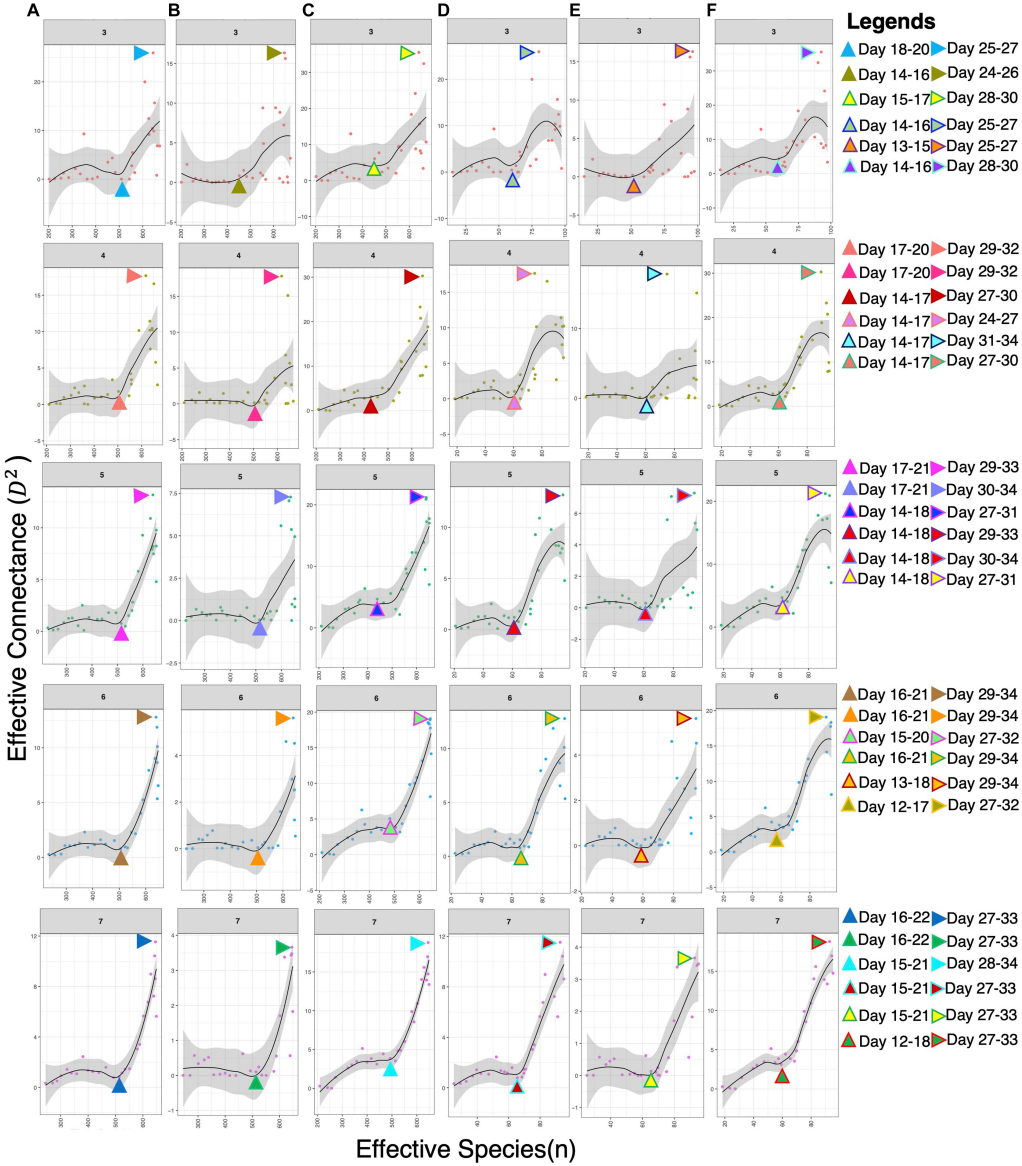
Stability-complexity relationship recovered for different dissimilarity measures and the ways in which the effective number of species *n* is calculated: **(A)** dissimilarity metric (DM): Jensen–Shannon divergence (RJSD); *n*: binary counting; **(B)** DM: *Euclidean distance*; *n*: binary counting; **(C)** DM: Spearman correlation; *n*: binary counting; **(D)** DM: RJSD; *n*: richness; **(E)** DM: Euclidean; *n*: richness; **(F)** DM: Spearman; *n*: richness. Since the method requires >35 samples, we have calculated the values on a moving window average for sizes 3, 4, 5, 6, and 7 days shown in the strips. For each window size and the chosen parameters, we have identified the days at which there is a drastic change in stability (represented by the triangle pointing up) with the values shooting up immediately after, and the days where there is maximum instability (represented by the triangle pointing right).

To estimate the interactions and complexity-stability profile of microbial communities within broiler guts, we calculated the values of effective connectance and effective number of species. Results were obtained in the form of a dissimilarity overlap curve for the moving window sizes of days 3, 4, 5, 6, and 7. A sudden shift in microbial communities observed during the 3rd week is more likely due to a change in diet or age of the bird, making communities more complex, dense, and unstable. This might result in the emergence or exclusion of some genera, thereby revealing the sensitivity of their abundances toward each other. High connectance values in the figure showed maximum instability in the 4th week, specifically after introducing the finisher diet (Figure 2).

**Figure 3:**
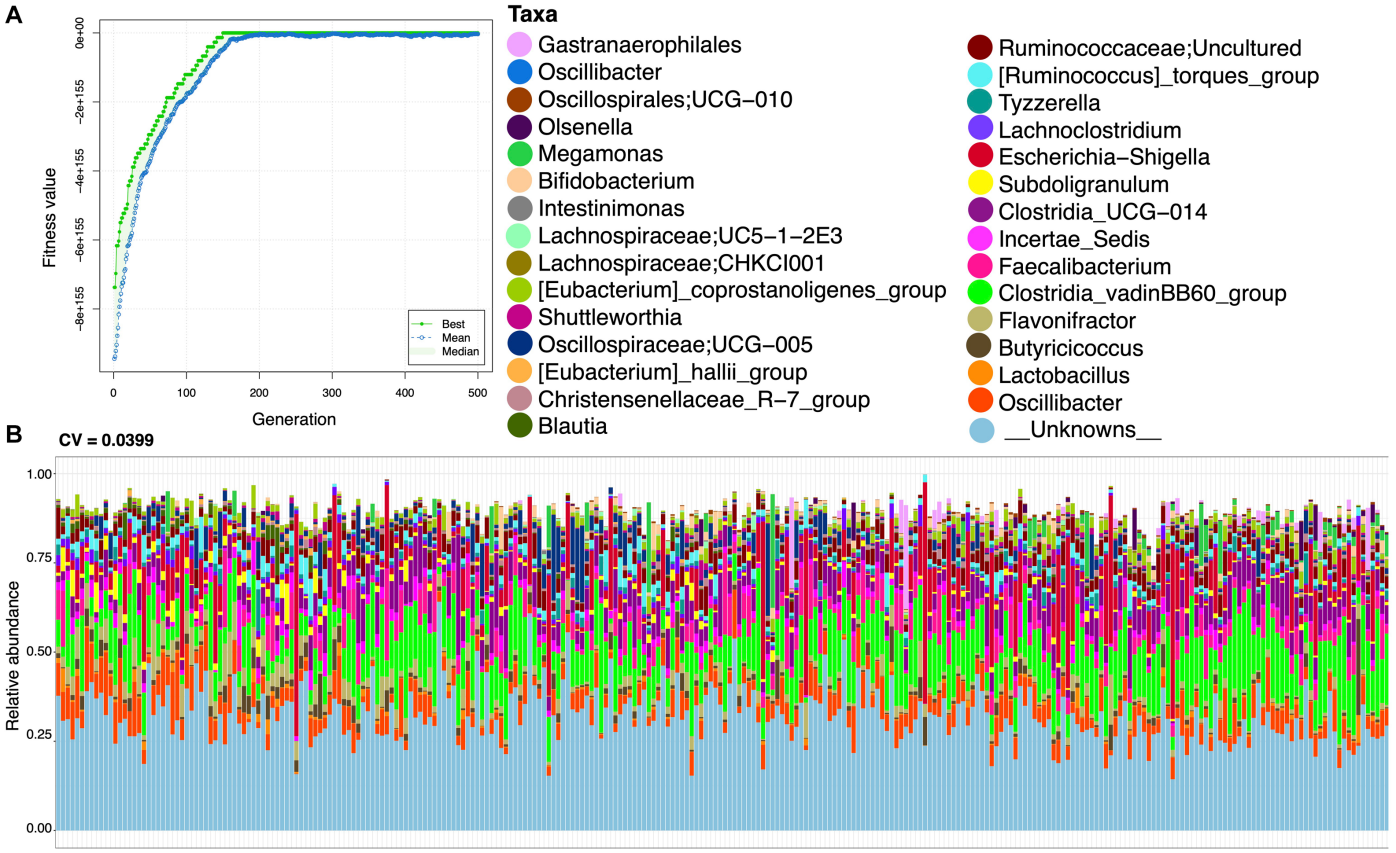
Stable ensemble returned after running the EQO algorithm in uniform phenotypic variable mode, with **(A)** showing the fitness value evolution of the genetic algorithm in finding these ensembles, highlighting the convergence to a steady state solution, and **(B)** showing the relative abundance profiles with coefficient of variation (CV) values given on the top of the plot. The lower CV value signifies higher stability. Here, we have only used the samples when the starter diet was given to the broiler.

**Figure 4:**
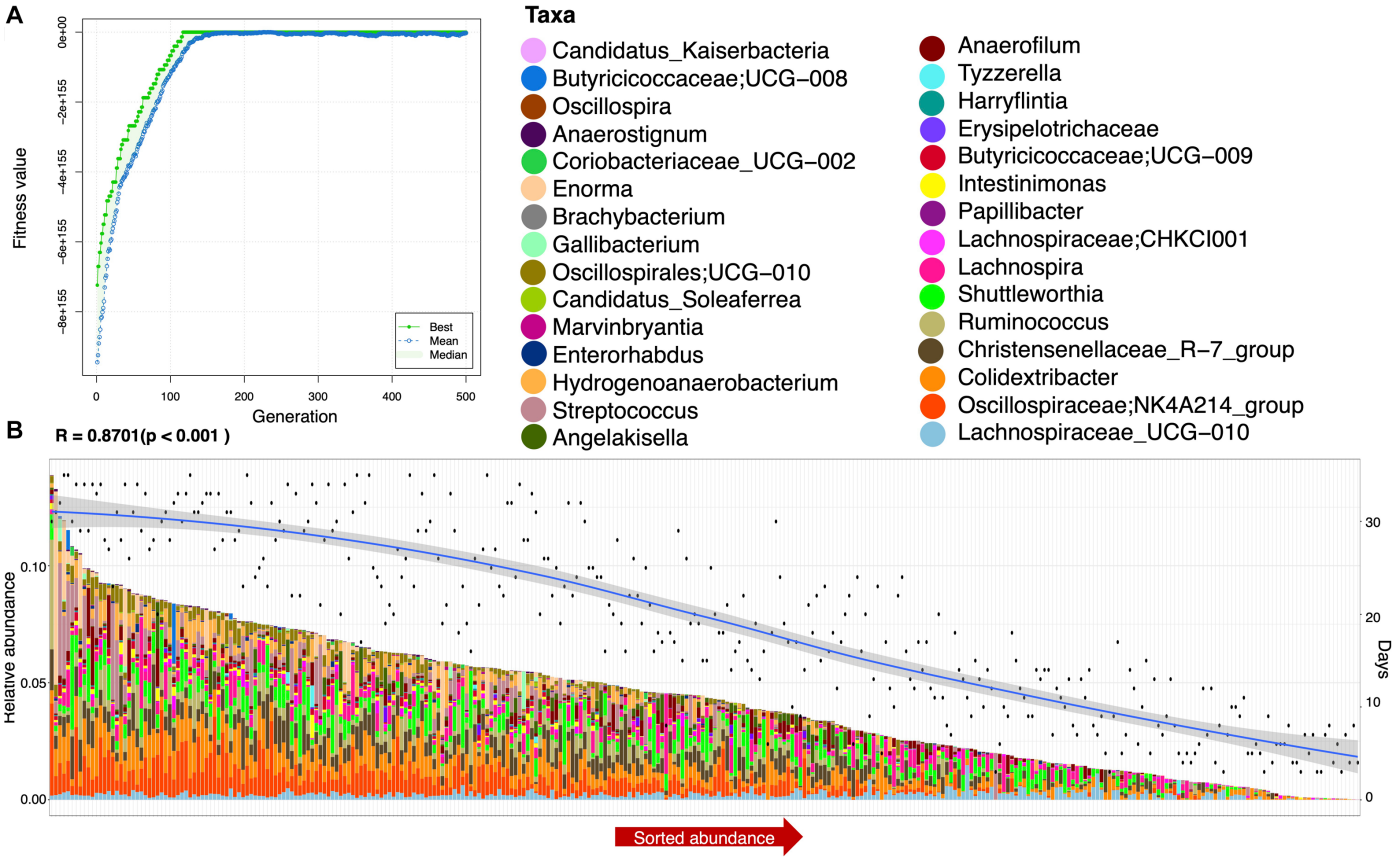
Ensemble (a minimal subset of genera) returned after applying the EQO technique using *days* as a continuous predictor. **(A)** Shows the fitness value evolution of the genetic algorithm in finding these ensembles, highlighting the convergence to a steady state solution, and **(B)** shows the sorted abundance of the ensembles with a biplot of smooth values of *days* (right *y*-axis) and shows the correlation value between the ensemble and *days* just above the plot. This has been performed for all samples.

To find functionally coherent taxa (ensemble) with stable and changed relative abundance, we applied a novel approach, i.e., ensemble quotient optimization (EQO). We identified bacterial genera with stable relative abundance and minimum coefficient of variation (CV = 0.0399), including major anaerobes as commensals, e.g., *Oscillibacter*, *Olsenella*, *Megamonas*, *Bifidobacterium*, *Lachnospiraceae*, *Shuttleworthia*, *Oscillospiraceae*, *Blautia*, *Lachnoclostridium*, *Subdoligranulum*, *Faecalibactrium*, *Butyricicoccus*, and *Lactobacillus* (Figure 3). We also identified genera with changed relative abundance and a high *correlation* (*R* = 0.8701) based on statistical variations in age (and other environmental factors provided in the Supplementary material) and gut microbiome composition (see Figure 4). In addition to others, *Colidextribacter*, *Oscillospiraceae*; *NK4A214_group*, *Intestinimonas*, and *Ruminococcus* were picked out as major genera with changed relative abundance throughout the productive age of birds (Figure 4).

**Figure 5:**
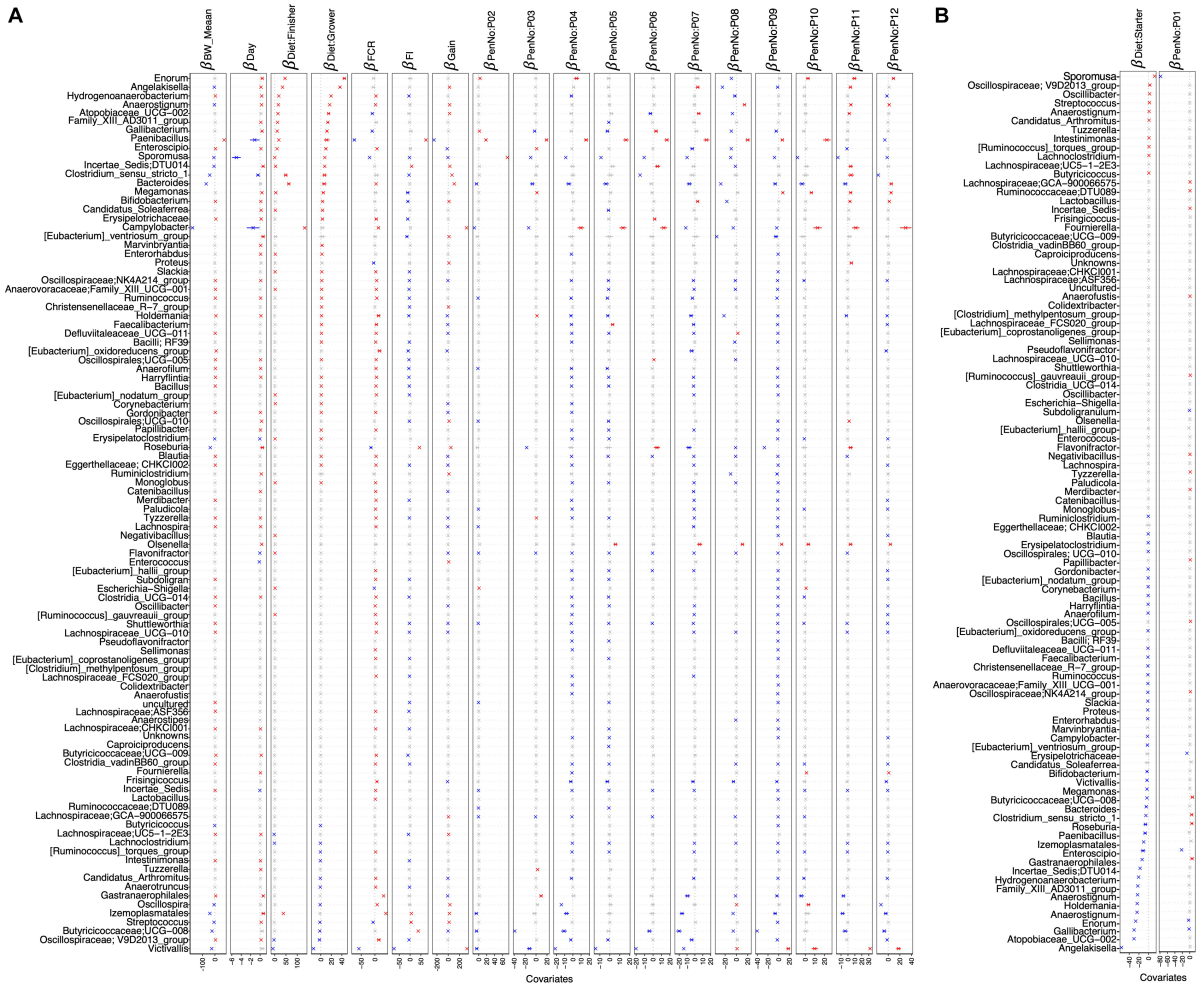
β -coefficients returned from the GLLVM procedure for covariates considered in this study. The top 100 most abundant genera were considered, incorporating both continuous data (*BW_mean*, *day*, *FCR*, *FI*, and *gain*) as well as categorical labeling of the samples (*pen* and *diet*). Those coefficients that are positively associated with the microbial abundance of a particular genus are represented in red color, while those that are negatively associated are represented in blue color, respectively. Where the 95% confidence interval of the β -coefficients crosses the 0 boundary, the coefficients are insignificant and are represented by a gray color. Since the collation of OTUs was performed at the genus level, all those OTUs that cannot be categorized based on taxonomy are collated under “__unknowns__” category. Incorporating a categorical variable in a regression model involves dropping one level as a reference when using one-hot encoding, and therefore, the algorithm was run twice: **(A)** with *diet*: *starter* and PenNo: P01 as references and **(B)** with *diet*: *grower* and PenNo: P02 as references. For **(B)**, the remaining coefficients are shown in Supplementary Figure S12.

Next, we applied a regression model, i.e., the generalized linear latent variable model (GLLVM), in order to identify genera that positively or negatively correlate with bird’s age (Figure 5A). Some SCFA-producing bacteria, including *Izemoplasmatales*, *Gastranaerophilales*, *(Eubacterium)_ventriosum_group*, and *Roseburia*, are found as top genera in positive correlation with age. Certain taxa, such as *Sporomusa*, *Campylobacter*, *Clostridium_sensu_stricto_1*, *Enterococcus*, and the phytopathogen *Paenibacillus*, negatively correlated with bird’s age, indicating a greater risk of disease in the initial days.

### 3.2. Taxonomic and functional structure (stable and changing) for different dietary regimes (*starter*, *grower*, and *finisher*)

After applying EQO to different dietary regimes, i.e., *starter*, *grower*, and *finisher*, genera with stable and changed relative abundances were identified. The genera with stable relative abundance in the initial 10 days when the *starter* diet was offered to the birds mostly include genera involved in feed digestion, such as *Oscillibacter*, *Clostridia_vadinBB60_group*, *Flavonifractor*, *Lachnoclostridium*, and *Faecalibacterium*. Broilers were also found to be at higher risk of some pathogenic genera, e.g., *Escherichia-Shigella*, during that time (Supplementary Figure S6). As the diet shifted from *starter* to *grower*, a change in stable genera was noticed, including *Tyzzerella*, *Clostridia_UCG-014*, *(Eubacterium)_coprostanoligenes_group*, and *Bifidobacterium* (Supplementary Figure S8). In addition to the previously mentioned genera, *Ruminococcus and (Ruminococcus)_torques_group* also stabilized their abundance when a *finisher* diet was offered (Supplementary Figure S10).

The genera identified with changed abundance in the *starter* diet period were *Subdoligranulum*, *Tyzzerella*, *Ruminococcaceae*; *DTU089*, *Oscillospiraceae*; *UCG-005*, and *(Eubacterium)_coprostanoligenes_group* (Supplementary Figure S7). As a *grower* diet was provided, *Angelakisella*, *Hydrogenonanaerobacterium*, *Oscillospirales*; *UCG-010*, *Butyricicoccaceae*; *UCG-009*, *Streptococcus*, and *Anaerofilum* were found in varied abundances (Supplementary Figure S9). It is suggested that the relative abundance of *Megamonas* and *Enorma* decreased when a *finisher* diet was provided (Supplementary Figure S11).

After applying the generalized linear latent variable model (GLVVM), *Oscillospiraceae*; *V9D2013_group*, and *Streptococcus* were found to be positively associated with the *starter* diet period but negatively associated with the *grower* and *finisher* diet periods. *Angelakisella* and *Coriobacteriaceae_UCG-002* are found as top genera associated positively with *grower* diet periods but negatively with *starter* diet periods. *Izemoplasmatales* are associated positively with the *finisher* diet but negatively with the *grower* diet. *Enorma* and *Victivallis* showed positive and negative associations, respectively, with *grower*/*finisher* diet periods. Moreover, *Sporomusa*, *Oscillospira*, and *Anaerotruncus* positively correlated with the *starter* diet. Similarly, *Hydrogenoanaerobacterium* showed a positive correlation with the *grower* diet. In addition, *Campylobacter*, *Clostridium_sensu_stricto_1*, and *Bacteroides* are among the top genera with a positive association with *finisher* diet. *Gallibacterium* is negatively correlated with the *starter* diet. *Butyricicoccaceae*; *UCG-008*, *Lachnospiraceae*; *UC5-1-2E3*, and *Lachnoclostridium* are negatively associated with *grower*/*finisher* diets (Figures 5A,B).

### 3.3. Performance parameters and their association with key microbes and functions

To identify the significant genera in terms of their increased or decreased abundance in association with performance parameters including *BW_mean*, *FI*, *FCR*, and *gain*, we applied two regression models, including CODA LASSO and GLLVM (Supplementary Table S1). From both of the analyses, we found key probiotic genera positively associated with BW_mean, including *Paenibacillus*, *Oscillospiraceae*; *V9D2013_group*, *Oscillospirales*; *UCG-010*, *NK4A214_group*, *UCG-005*, *Holdemania*, *Shuttleworthia*, *Bifidobacterium*, *Colidextribacter*, and *Butyricicoccaceae*; *UCG-009*. In contrast, some pathogenic anaerobes are found to be in negative association with BW_mean, such as *Victivallis*, *Campylobacter*, *Sporomusa*, *Clostridium_sensu_stricto_1*, *Anaerofustis*, and *Anaerovoracaceae*; *Family_XIII_AD3011_group* (Figure 4A; Supplementary Figure S20).

Some major commensals, including *Oscillospiraceae; V9D2013_group*, *Oscillospirales; UCG-010*, *NK4A214_group*, *UCG-005*, *Butyricicoccaceae*; *UCG-008*, *Roseburia*, *Shuttleworthia*, *Hydrogenoanaerobacterium*, *Fournierella*, and *Colidextribacter* are related to improved gut health and increased *FI*. We also found some probiotic genera negatively associated with *FI* (improved FCR), including *Holdemania*, *Eubacterium_ventriosum_group*, *nodatum_group*, *hallii_group*, *Bifidobacterium*, *Lachnospiraceae*; *GCA-900066575*, *ASF356*, *Eubacterium_oxidoreducens_group*, *Ruminiclostridium*, *Allisonella*, *Lachnospiraceae*; *UC5-1-2E3*, *FCS020_group*, *Erysipelotrichaceae*, *Anaerofustis*, *Butyricicoccaceae*; *UCG-009*, and *Subdoligranulum* (Figure 5A; Supplementary Figure S22).

Increased abundance of some beneficial genera such as *Oscillospiraceae*; *V9D2013_group*, *Oscillospirales*; *UCG-010*, *NK4A214_group*, *UCG-005*, *Roseburia*, *Shuttleworthia*, *Oscillospira*, *Bifidobacterium*, *Anaerovoracaceae*; *Family_XIII_UCG-001*, *Fournierella*, and *Colidextribacter* are noticed, resulting in more gain. Some commensals are also found to be negatively related to gain (Figure 5A; Supplementary Figure S21).

In terms of *FCR*, major commensals are found to be in negative association, including *Paenibacillus*, *Sporomusa*, *Roseburia*, *Lachnospiraceae*; *GCA-900066575*, *ASF356*, *(Clostridium)_methylpentosum_group*, and *Clostridiaceae*; *Candidatus_Arthromitus*, which may result in improved feed efficiency and more weight gain (Figure 5A; Supplementary Figure S23).

### 3.4. Predictive antimicrobial resistance evolution and key relationships with predictors

**Figure 6:**
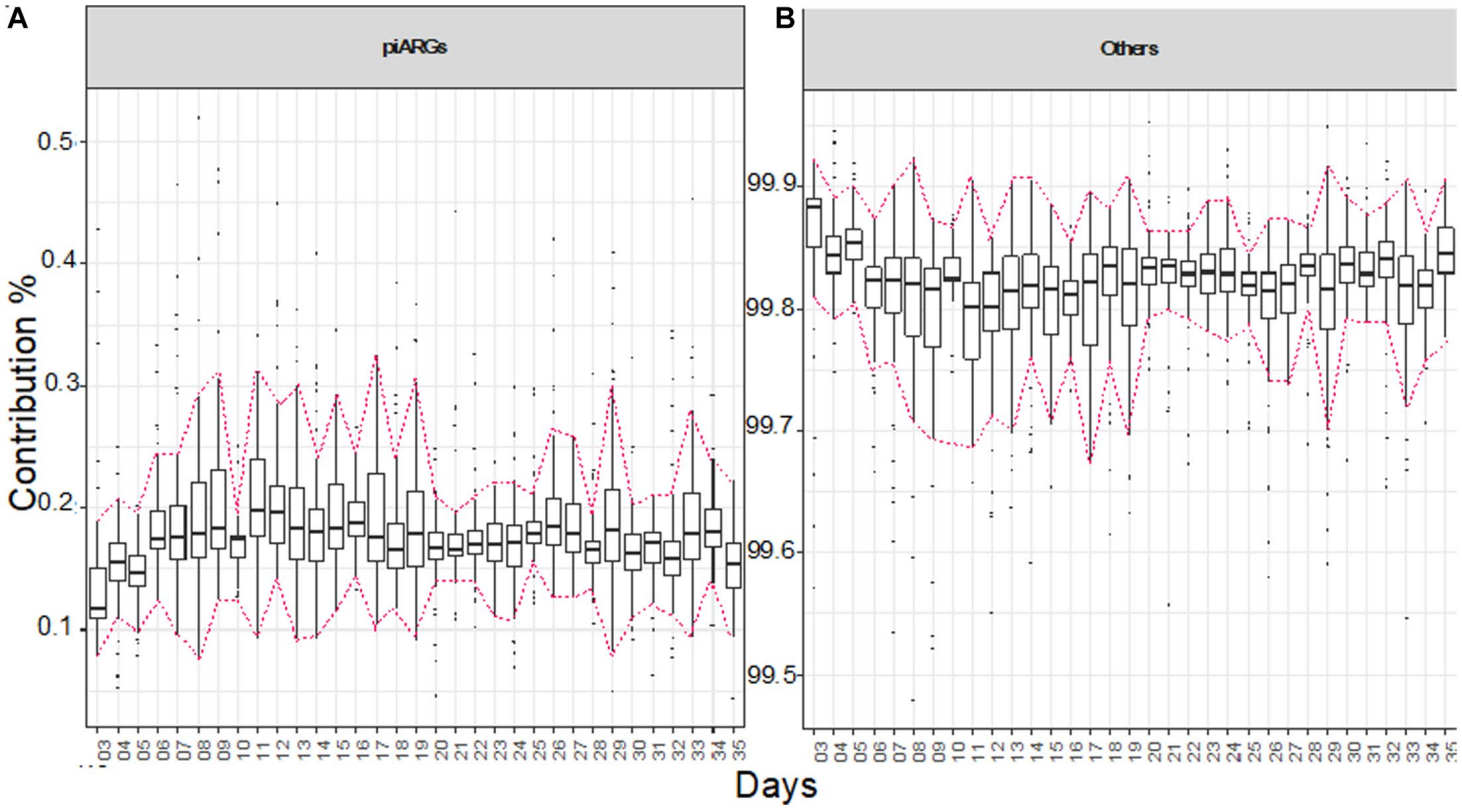
Bray–Curtis contribution split of KEGG genes to piARGs genes **(A)** and the rest of the genes **(B)**. For each day, the plot shows N(N−1)/2 pair-wise comparison of samples (*N* ≅ 12 pens/day). The highlighted area shows when the diets were administered, in chronological order as *starter*, *grower*, and *finisher* diets. Tukey’s honest significant difference (HSD) test as a *post-hoc* test was used to assess if the contribution by piARG KOs was significantly different between the days, with the details given in Supplementary Table S5.

To find the contribution of predictive antimicrobial resistance genes (piARGs) and other genes for the whole experimental period, we applied Bray–Curtis contribution analysis, doing pairwise comparisons (Figures 6A,B). Overall, piARGs contribute at a stable level, but there is a slight increase in abundance, particularly when the diet is changed. An increase in piARGs abundance can be seen on day 11 when the *grower* diet was introduced. In the initial 10 days until the *starter* diet was offered, it was observed using EQO analysis that genera like *Oscillibacter*, *Clostridia_vadinBB60_group*, *Flavonifractor*, *Lachnoclostridium*, and *Faecalibacterium* were found in most abundance, and here, we can predict that these genera are involved in contributing the piARGs. Similarly, the percentage of piARGs increased in the *finisher* diet period, and during this period, *Ruminococcus and (Ruminococcus)_torques_group* also stabilized their abundance, so we can predict that these genera are contributing more piARGs. In general, piARG’s contribution increased in the initial couple of weeks and then stabilized for the rest of the life span.

**Figure 7:**
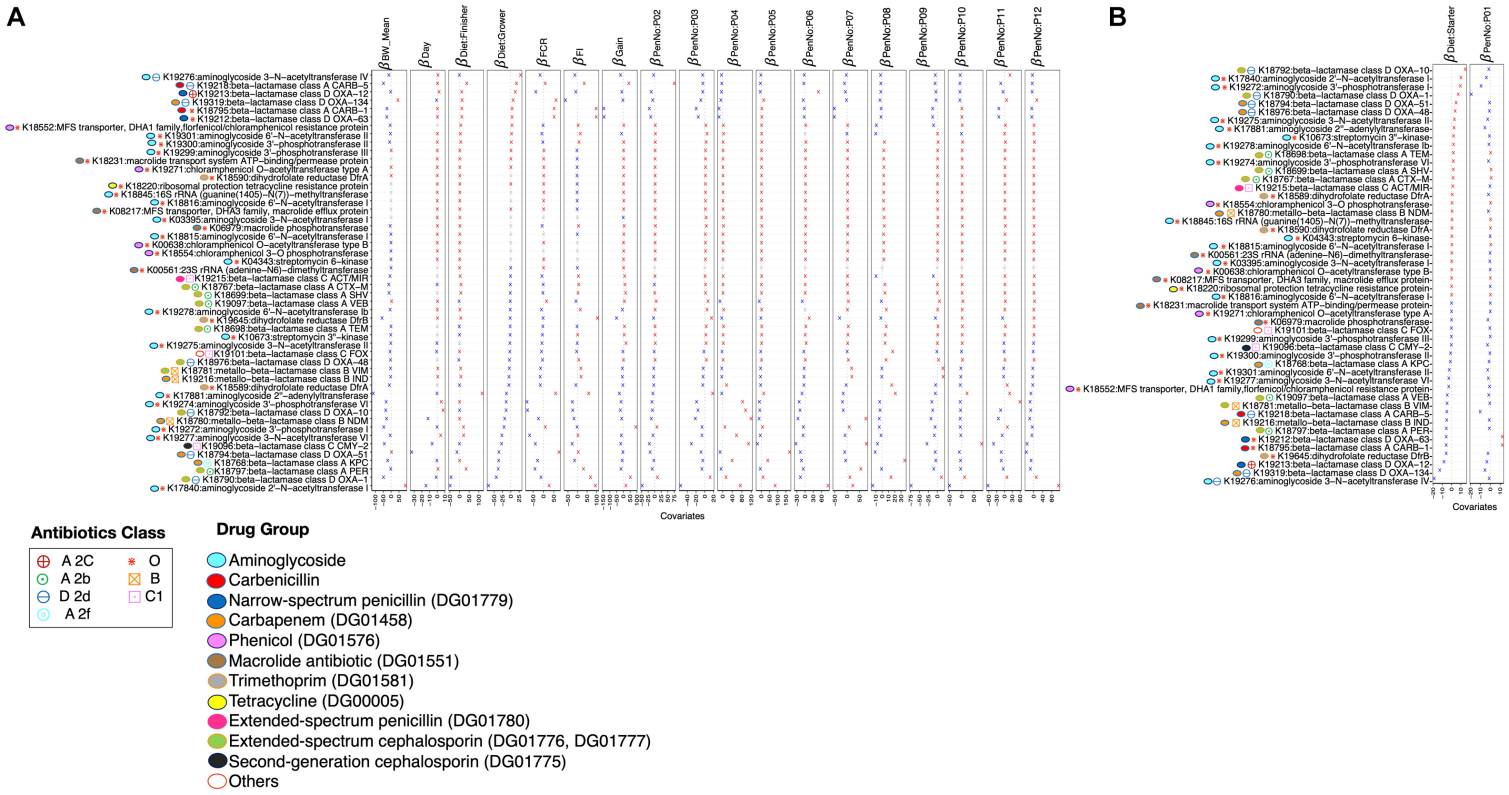
β -coefficients returned from the GLLVM procedure for covariates considered in this study by considering 50 out of 90 reference piARGs KEGG orthologs (KOs) detected in this study using the PICRUSt2 procedure. Those coefficients that are positively associated with the abundance of a particular piARG KO are represented in red color, while those that are negatively associated are represented in blue color. Where the 95% confidence interval of the β -coefficients crosses the 0 boundary, the coefficients are insignificant and are represented by a gray color. Similar to Figure 4, the algorithm was run twice: **(A)** with diet: *starter* and PenNo: P01 as references and **(B)** with diet: *grower* and PenNo: P02 as references. For **(B)**, the remaining coefficients are shown in Supplementary Figure S13. Additional annotations of threat levels, classes, and drug groups; these piARGs are categorized.

Considering performance parameters, i.e., *BW_mean*, *FI*, *FCR*, *gain*, and different *pens* in which birds were allocated, as environmental covariates, the abundance of piARGs considering KEGG orthologs (KOs) was analyzed using GLLVM (Figures 7A,B and Supplementary Table S2). A total of 34 piARGs were found to have a positive correlation with the defined variables, out of which 20 belong to the drug group of beta-lactams, including carbapenems, extended-spectrum cephalosporins, extended-spectrum penicillin, and monobactams; 12 belong to aminoglycoside drug groups; and 2 belong to trimethoprim.

We also identified 24 piARGs with a negative correlation to defined variables, out of which 15 belong to the beta-lactam drug group, including carbapenem, extended-spectrum cephalosporins, extended-spectrum penicillin, and monobactams; 7 belong to the aminoglycoside drug group; 1 belongs to trimethoprim; and 1 belongs to phenicol-resistant protein.

The piARGs are positively associated with the species that are categorized in a group with serious threats by the CDC, which includes multidrug-resistant *Acinetobacter*, multidrug-resistant *Pseudomonas aeruginosa*, drug-resistant non-typhoidal *Salmonella*, drug-resistant *Salmonella enterica serovar Typhi*, drug-resistant *Shigella*, and drug-resistant *Streptococcus pneumoniae*.

In general, the majority of the piARGs belonging to the drug group of aminoglycosides (DG01447) are upregulated in the *finisher* diet period and are also positively correlated with most performance parameters. In contrast, piARGs belonging to the drug group of carbapenems (DG01458) are found to be downregulated in *grower* diet/*finisher* diet periods and negatively associated with most performance parameters. piARGs belonging to extended-spectrum cephalosporin (DG01776 and DG01777) and penicillin (DG01780) are found to be more abundant in the *starter* diet period but less abundant in the *finisher* diet period, and they also show a negative correlation with the age of the birds. It has been made evident that the drug groups of second- and third-generation cephalosporin (DG01775 and DG01776) are positively correlated with *BW_mean* of birds. Extended-spectrum cephalosporin (DG01776 and DG01777) and monobactam (DG01454) are negatively associated with the *grower* and *finisher* diet periods. Carbenicillin (DG00519) showed a positive association with the *grower* and *finisher* diet periods but a negative association with the *starter* diet period. Some KOs belonging to trimethoprim (DG01581), extended-spectrum penicillin (DG01780), second-generation cephalosporin (DG01775), phenicol (DG01576), and cephalosporins (no DG number) are positively or negatively associated with some of the pens (e.g., P08).

To determine how age (*days*) and different performance parameters (*BW_mean*, *FCR*, *FI*, and *gain*) contribute to the functional profiles of bacterial communities, we applied the *CODA-LASSO* regression model to recovered *metacyc* pathways (Supplementary Tables S3, S4). The majority of pathways involved in amino acid biosynthesis are found to be positively correlated with bird’s age such as L-histidine biosynthesis, L-arginine biosynthesis I (via L-ornithine), and the superpathway of taurine degradation. Some nucleotide biosynthesis/repair pathways, like guanosine ribonucleotides *de novo* biosynthesis and pyrimidine deoxyribonucleotides *de novo* biosynthesis II, were also found to have a positive correlation with the age of birds. Additionally, a peptidoglycan biosynthesis pathway and a coumarin biosynthesis pathway, most likely conferring some antibiotic resistance, were also found among the top positively correlated pathways to age. In contrast, pathways related to cofactors/coenzymes biosynthesis like tetrapyrrole biosynthesis I (from glutamate), foliate transformations III (*E. coli*), and nicotinamide adenine dinucleotide (NAD) biosynthesis are found to be negatively associated with age (Supplementary Figure S19). Age progression was positively associated with pathways involved in protein, DNA, and peptidoglycan biosynthesis, while pathways related to cofactors/coenzymes biosynthesis were negatively associated with most of the performance parameters (Supplementary Figures S20–23).

### 3.5. Environmental niche breadth and overlap

From the *GLLVM*, we estimated the effect of different environments, considering the 12 *pens* in which birds were allocated (Supplementary Figure S1), on the abundance of major bacterial genera. Some commensals are found in positive association with most of the pens, including *Paenibacillus, Enorma, Roseburia, Olsenella*, and *Megamonas*. Similarly, some other commensals such as *Oscillospiraceae*; *V9D2013_group*, *Oscillospirales*; *UCG-010*, *Bacteroides, Butyricicoccaceae*; *UCG-008*, and *Shuttleworthia* were detected in negative relation to some pens. Some pathogenic genera, such as *Victivallis* and *Campylobacter*, were also found to be either positively or negatively associated with certain pens (Figures 5A,B and Supplementary Table S1).

**Figure 8:**
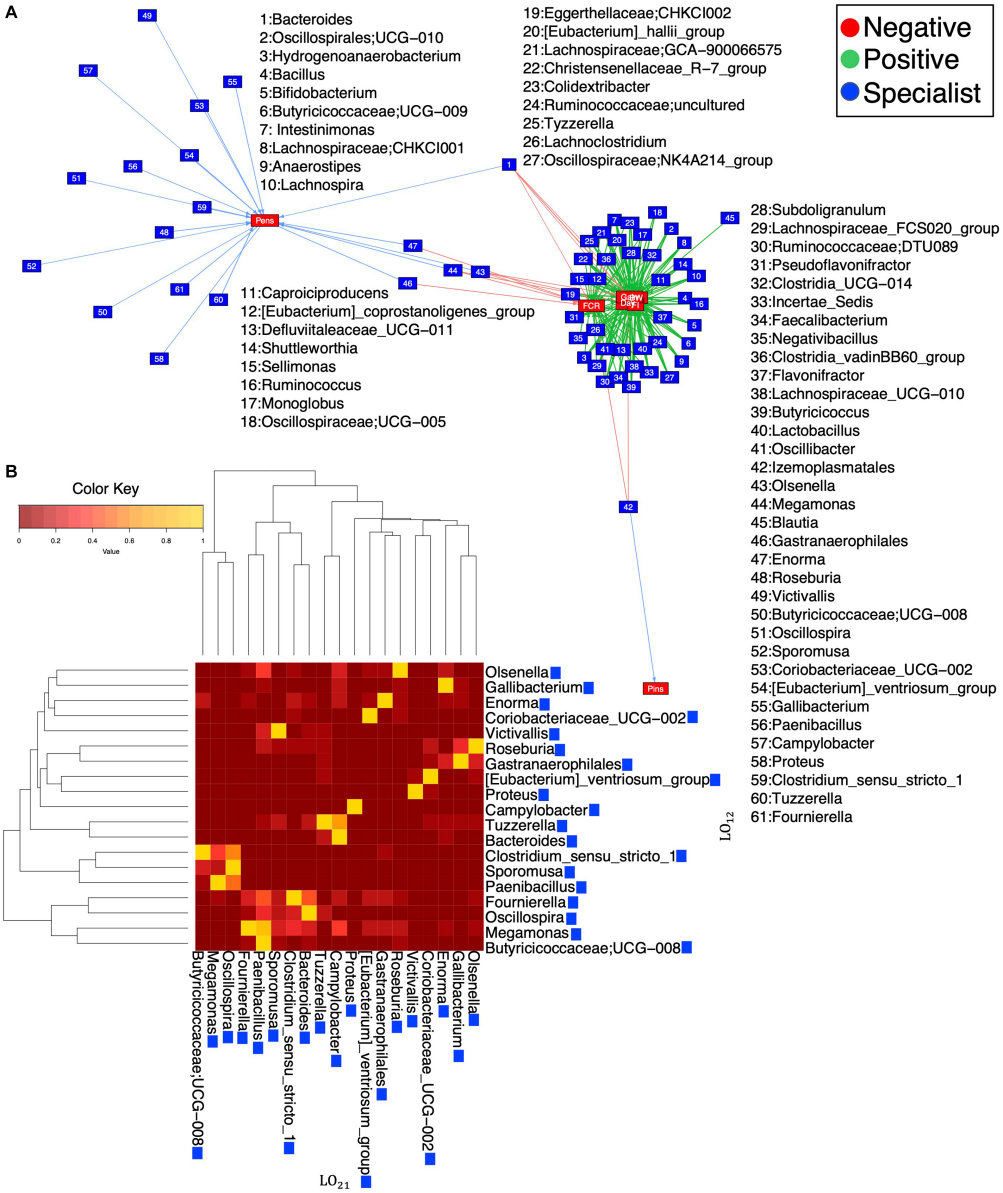
Network of relationships **(A)** recovered after applying Levin’s *B*_N_ to find generalists and specialists and Hurlbert’s *B*_N_ to find positive and negative associations with respect to environmental properties. These properties are *mean body weight* (*BW_mean*), *feed intake* (FI), *feed conversion ratio* (FCR), *gain*, and *days* calculated on different sets of environments (*R* ≅ 12 pens). The genera selected from Levin’s *B*_N_ are further used to calculate Levin’s overlap **(B)**, with blue boxes signifying that they are all specialists. See further details in Supplementary Figures S17, S18.

We applied Levin’s *B*_N_ to find bacterial genera that are generalists or specialists, considering *pens* as different sets of environments. We did not find any generalists, but all the above-mentioned genera were found as specialists in addition to others. Then, we applied Hulbert’s *B*_N_ to find positive and negative associations between specialist genera and environmental properties, i.e., *BW_mean, FI*, *FCR*, *gain*, and *days* (Supplementary Figures S17, S18), within different environments. Furthermore, genera were selected from Levin’s *B*_N_ to calculate Levin’s overlap, highlighting some specialists including *Gallibacterium*, *Enorma*, *Coriobacteriaceeae_UCG-002*, and *Proteus* (Figures 8A,B).

**Figure 9:**
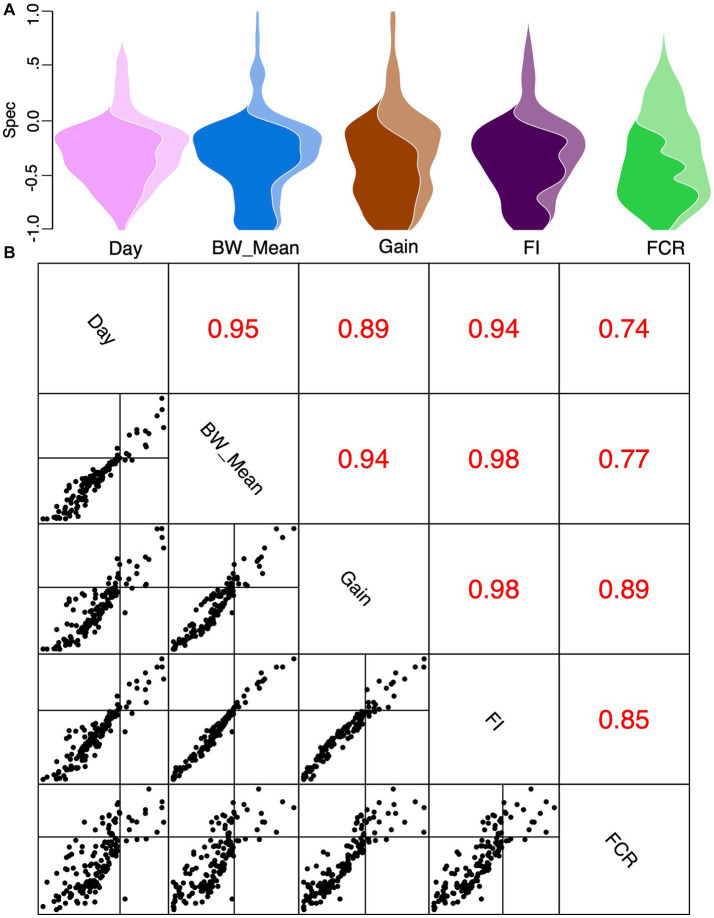
Specificity values *Spec* shown as a violin plot with the area divided between genera with statistically significant specificity (dark) versus genera without (light) for different covariates considered in this study **(A)**. Pairwise *Spec* correlations **(B)** with correlation coefficients (*R*) for each pairwise comparison of covariates are shown in this subplot’s upper triangles for the data plotted in the lower triangle of this subplot.

To explore if certain genera exist within a narrow range of covariates (*days*, *BW_mean*, *FI*, *FCR*, and *gain*) and different dietary regimes (*starter*, *grower*, and *finisher*), we used a null modeling approach to calculate the “*spec*” number. This number offers a threshold to decide between *cosmopolitan* (>0) and *specific* (<0) in Figure 9A. Pairwise comparisons of all covariates were done to calculate correlation coefficients, indicating a high correlation between covariates (Figures 8A,B; Supplementary Figures 24–31). We found that gut microbial communities exhibited strong and statistically significant specificity to several environmental variables, in particular *days* (bird’s age), which implicitly capture the underlying dietary regimen. Genera found specific to the *starter* diet period were *Brebundimonas*, *Brevibacillus*, *Sphingopyxis*, *Allorhizobium-Neorhizobium-Paraihizobium-Rhizobium*, *Paenibacillus*, *Pediococcus*, *Trachelomonas*, and *Cupriavidus*. Specific genera in the *grower* diet period include *Microbacterium*, *Allisonella*, *Campylobacter*, *Rhodococcus*, *Lysinibacillus*, and *Mesorhizobium*. In the *finisher* diet period, *Oscillospiraceae*; *UCG-007*, *Bacteroides*, *Gallibacterium*, *Izemoplasmatales*, *Incertae_Sedis*; *DTU014, DTU014, DTU014*, *Romboutsia*, *Gastranaerophilales*, *Victivallis*, *Butyricicoccaceae*; *UCG-008*, *Oscillospira*, *Coriobacteriaceae_UCG-002*, and *Enteroscipio* found as specific genera (Supplementary Figures S24, S25).

## 4. Discussion

At the global level, chicken, being the dominant livestock, has the optimal calorie retention when considering feed conversions (Fry et al., 2018). Productivity in terms of weight gain and disease resistance is highly influenced by gut bacterial communities. Here, in this manuscript, we elucidate the spatial temporal dynamics of broiler cecal microbiota development by emphasizing on stability and complexity of bacterial species as different diets are introduced in their development cycle (from day 3 to day 35). In doing so, we are also able to ascertain the niches some of the species occupy and establish links between gut bacterial profiles and environmental (performance) covariates.

Our results demonstrate that gut communities are majorly influenced by the age of birds, highlighting the significance of different growth periods in microbial community assembly mechanisms, which is also supported by previous studies (Crhanova et al., 2011; Danzeisen et al., 2011; Oakley et al., 2014; Mohd Shaufi et al., 2015; Pourabedin et al., 2015; Joat et al., 2021; Xiao et al., 2021; Zou et al., 2022). The age of birds is found to be the most important factor that may affect the stability of microbial communities. Our results suggest that based on the interaction of bacterial species, the microbiota starts to become unstable at the onset of the 3rd week, with instability gradually increasing. Instability is also previously reported beyond the 5th week (Lu et al., 2003; Feye et al., 2020; Li et al., 2022), which affects the growth performance of the bird. We explored some ecological relationships between microbial communities and found functionally coherent taxa with respect to their relative abundances (uniform or varied). In the majority of cases, anaerobic bacteria dominated as commensals and were identified as members of a stable or uniform ensemble, including *Oscillibacter*, *Megamonas*, *Shuttleworthia*, *Blautia*, *Lachnoclostridium*, *Subdoligranulum*, *Faecalibactrium*, *Butyricicoccus*, *Lactobacillus, Olsenella*, and *Bifidobacterium*. While some anaerobes, such as *Oscillospiraceae*; *NK4A214_group*, *Intestinimonas*, and *Ruminococcus* were found to be part of a changing ensemble with varied relative abundances. Most of the genera are involved in saccharolytic fermentation and belong to *Firmicutes*, the dominant phylum, which has also been braced by many microbiome studies in the preceding decade (Danzeisen et al., 2011; Choi et al., 2014; Xiao et al., 2017; Clavijo and Flórez, 2018; Venegas et al., 2019; Mandal et al., 2020; Han et al., 2022). Furthermore, genera having a positive or negative correlation with age were also highlighted through regression models, i.e., *GLLVM*. Some genera, predominantly short-chain fatty acids (SCFAs) producers, e.g., *Izemoplasmatales*, *Gastranaerophilales*, *(Eubacterium)_ventriosum_group*, and *Roseburia* were found to have increasing species abundance as birds matured, and vice versa. Certain taxa, such as *Sporomusa*, *Clostridium_sensu_stricto_1*, and *Enterococcus*, negatively correlated with the bird’s age. These findings indicate that birds are potentially at higher risk of disease and death (especially under poor management practices) in their initial days, as supported by some studies (Hafez and Attia, 2020; Yerpes et al., 2020). Although there are disparities in the reported literature, the major factors often implicated in microbial compositional changes include rearing periods, genetics, sampling sections of gastrointestinal tracts, age, feed, and management practices (Lu et al., 2003; Ballou et al., 2016; Ranjitkar et al., 2016; Wang et al., 2016; Ngunjiri et al., 2019).

In addition to applying EQO to the whole 33 days time window, we wanted to know what remained stable during different diet periods. We found the diet to drive changes in microbial communities, particularly when they changed. Studies have demonstrated the effect of dietary composition along with food particle size on chicken gut microbial populations (Apajalahti et al., 2001, 2004). The genera with stable relative abundance in the initial 10 days (*starter* diet period) mostly included anaerobic *Firmicutes* and *Bacteroidetes* such as *Oscillibacter*, *Clostridia_vadinBB60_group*, *Flavonifractor*, *Lachnoclostridium*, and *Faecalibacterium*. The majority of them were previously found to be involved in proteolytic fermentation, producing SCFAs, and branched chain fatty acids (BCFAs) (Macfarlane et al., 1992; Smith and Macfarlane, 1998; Aguirre et al., 2016). We hypothesize that these findings are a consequence of common dietary practices at poultry farms where the *starter* feed is typically rich in protein composition (>20%) (Esmail, 2016). Note that, in the initial days, birds are found to be at higher risk of being exposed to pathogenic genera such as *Escherichia-Shigella*. When birds are shifted to a *grower* diet, a change in stable genera is noticed, with the major inclusion of commensals such as *Tyzzerella*, *Clostridia_UCG-014*, *(Eubacterium)_coprostanoligenes_group*, and *Bifidobacterium*. Likewise, some cellulolytic genera, including *Ruminococcus and (Ruminococcus)_torques_group* also stabilized their abundance when the *finisher* diet was introduced. The genera with varied abundance in the *starter* diet period are predominantly SCFA producers, including *Subdoligranulum*, *Tyzzerella*, *Ruminococcaceae*; *DTU089*, *Oscillospiraceae*; *UCG-005*, and *(Eubacterium)_coprostanoligenes_group*. Some commensals as well as pathogens, including *Angelakisella*, *Hydrogenonanaerobacterium*, *Oscillospirales*; *UCG-010*, *Butyricicoccaceae*; *UCG-009*, *Streptococcus*, and *Anaerofilum*, are found to have changed abundance during the *grower* diet period. It was also found that the abundances of some commensals, such as *Megamonas* and *Enorma*, went down when the *finisher* diet was introduced.

To further our understanding of different genera in the context of sources of variation, a regression model (GLVVM) was applied to fit the abundance of each genus against all sources of variability that we observed, primarily focusing on different diets. Strict anaerobes like *Oscillospiraceae*; *V9D2013_group* were found to be positively associated with the *starter* diet but negatively associated with the *grower* and the *finisher* diets. This is in line with studies revealing that initial chicken gut colonizers are mostly facultative anaerobes, followed by strict anaerobes specifically after day 3 (Rehman et al., 2007; Stanley et al., 2014). Some anaerobes most likely involved in energy production, such as *Angelakisella* and *Coriobacteriaceae_UCG-002*, are found to be the top genera associating positively with the *grower* diet but negatively with the *starter* diet. This is supported by the fact that *grower* diets are high in energy content as compared to the *starter* diet (Yücel and Taşkin, 2018). *Enorma* has shown a positive association with *grower/finisher* diets, which was previously found to be involved in the production of micronutrients (Khan and Chousalkar, 2020; Gong et al., 2021). Moreover, some probiotic anaerobes, including *Sporomusa*, *Oscillospira*, and *Anaerotruncus*, were positively associated with the *starter* diet. A positive association of *Hydrogenoanaerobacterium* with the *grower* diet was also observed in (Clavijo et al., 2022), where they showed a strong association with the diet and age of broilers. In addition to some opportunistic pathogens like *Campylobacter* and *Clostridium_sensu_stricto_1,* saccharolytic bacterial genera such as *Bacteroides* are among the top genera with a positive association with the *finisher* diet. Gamma proteobacteria, i.e., *Gallibacterium*, was found to be positively associated with the *grower* diet and was previously implicated in many avian species (El-Adawy et al., 2018). Additionally, some anaerobic genera like *Butyricicoccaceae*; *UCG-008*, *Lachnospiraceae*; *UC5-1-2E3*, and *Lachnoclostridium* were negatively associated with both *grower* and *finisher* diets.

Genera that had increased or decreased abundances in association with the performance parameters were identified through two regression models, i.e., CODA LASSO and GLLVM. We found key probiotic genera positively associated with *BW_mean*, *FI*, *gain*, and *FCR*, including *Paenibacillus*, *Oscillospiraceae*; *V9D2013_group*, *Oscillospirales*; *UCG-010*, *NK4A214_group*, *UCG-005*, *Lachnospiraceae*; *GCA-900,066,575*, *ASF356, Holdemania*, *Roseburia, Fournierella*, *Shuttleworthia*, *Bifidobacterium*, *Colidextribacter*, and *Butyricicoccaceae*; *UCG-009, UCG-008*. The majority of these were previously associated with improved growth performance in chickens as they increased resistance to infections and balanced the gut ecosystem (Perić et al., 2010; Amara and Shibl, 2015; Sarangi et al., 2016; Wang et al., 2017). In parallel, we have also found some understudied but most likely pathogenic genera in a negative association with the growth parameters, such as *Victivallis*, *Sporomusa*, *Clostridium_sensu_stricto_1*, *Anaerofustis*, and *Anaerovoracaceae*; *Family_XIII_AD3011_group*.

In addition to finding positive and negative associations between microbial species and the continuous predictor variables, a specificity analysis was performed. This unraveled proliferation of microbes within a narrow “*specific*” range, e.g., bird’s age, coincided with time periods when the diets were introduced and are in line with the literature (van der Wielen et al., 2002; Hiett et al., 2013; Cui et al., 2017). Note that some of the variables had a very strong correlation, and thus species that were found to be specific to *mean body weight* are likely to be similar to those found to be specific to *days*. Indeed, the same is true for other parameters, albeit to a lesser extent. Genera that were found to be specific to the *starter* diet, albeit when birds were of low weight, were non-fermenting aerobic autotrophs such as *Brebundimonas*, *Brevibacillus*, *Sphingopyxis*, and *Cupriavidus* (García-Romero et al., 2016; Ryan and Pembroke, 2018; Amaresan et al., 2020; Jahn et al., 2021). Other genera were either obligate aerobes or anaerobes and were found to be specific to the *grower* diet when the birds had already gained weight. These are *Microbacterium*, *Allisonella*, *Campylobacter*, *Rhodococcus*, *Lysinibacillus*, and *Mesorhizobium*. Furthermore, *Oscillospiraceae*; *UCG-007*, *Bacteroides*, *Gallibacterium*, *Izemoplasmatales*, *Incertae_Sedis*; *DTU014, DTU014, DTU014, Romboutsia*, *Gastranaerophilales*, *Victivallis*, *Butyricicoccaceae*; *UCG-008*, *Coriobacteriaceae_UCG-002*, and *Enteroscipio* were found to be specific to the *finisher* diet.

Next, we wanted to see if there was a selection of predictive antimicrobial genes (piARGs) in the observed range. We found that bacterial community structure predicts the growth performance of the broiler, but in parallel, these communities are also contributing to antimicrobial resistance. As we have reported above, *Firmicutes* are the major phyla found in the gut microbial communities, and we hypothesize that they may be the major contributor of piARGs as depicted earlier (Juricova et al., 2021; Li et al., 2022; Xu et al., 2022). It has been observed in our results that most of the piARGs belonging to aminoglycosides (DG01447) were positively associated with the *finisher* diet period and also with the majority of the performance parameters, such as body weight. In a similar manner, we also observed that piARGs belonging to carbapenems (DG01458) were negatively associated with the *grower/finisher* diets and also with performance parameters. When the diet changed from *starter* to *grower* on day 11, a slight increase in piARGs was observed, indicating the effect of the feed change. A similar trend was observed on day 26 when the diet was switched to *finisher*, followed by a slight decrease in the 5th week. In general, the abundance of piAGRs remained stable throughout the experimental period. This is in line with recent studies that piARGs contribution is a continuous process effectuated by microbial communities themselves but is invariably influenced by the use of specific antimicrobials during the growth period (Thomas et al., 2017; Skarżyńska et al., 2020; Li et al., 2022). Since the diets were proprietary and we had no information about which antibiotics were used, with these results, we can only hypothesize that the predicted piARGs give sufficient evidence to suggest the above antibiotics were used. Our results indicate that the abundance of piARGs is influenced by the change in bacterial community structure, which we witnessed on days 11 and 26. It has been reported that the use of antimicrobials during the growth of broilers changes the gut microbial communities and enhances their resistance (Allen and Stanton, 2014; Xiong et al., 2018).

Overall, piARGs (specifically belonging to ß-lactams and aminoglycosides) abundance increased with the age of broilers, which may be linked with the increased microbial richness, as supported by previous studies (Butaye et al., 1999; Ozaki et al., 2011; Temmerman et al., 2022). piARGs belonging to carbapenems (DG01458) are found to decrease with the age of birds and are also negatively associated with the majority of the performance parameters. Our results are in agreement with the studies performed in the broiler in which it has been observed that ARGs decrease with age (Linton et al., 1982; Gaire et al., 2021). Many studies suggest that using antimicrobials as growth promoters or prophylaxis during the growth and production periods ultimately contributes to the resistance against the same antimicrobial class (Luiken et al., 2019; Gupta et al., 2021; De Cesare et al., 2022). As the diet used in this study was proprietary and we were not able to obtain information regarding antibiotic supplementation within the feed, it is not possible to determine whether the ARG enrichment observed with the diet was due to a change in bacterial communities because of protein content variation or due to antibiotic supplementation. However, it has been observed that AGP discontinuation in feed leads to decreased antibiotic selection pressure, and the resultant antibiotic resistance profiles of intestinal bacteria change rapidly (Wang et al., 2020). Antibiotic alternatives in broiler production include phytogenics, organic acids, prebiotics, probiotics, enzymes, and their derivatives. Antibiotic alternatives have been reported to increase feed intake, stimulate digestion, improve feed efficiency, increase growth performance, and reduce the incidence of diseases by modulating the intestinal microbiota and immune system, inhibiting pathogens, and improving intestinal integrity (Ayalew et al., 2022). However, how these interventions modulate the resistance within the chicken gut needs to be explored further.

We also explored how age and different performance parameters contribute to the functional profiles of bacterial communities through the *CODA-LASSO* regression model on the recovered *metacyc* pathways. Amino acid biosynthesis pathways such as L-histidine biosynthesis, L-arginine biosynthesis I (via L-ornithine), and the super-pathway of taurine degradation are found to be positively associated with a bird’s age. It has been reported that these amino acids are involved in the growth of the bird, specifically during the early days, and also play an important role in managing stress responses (Chowdhury et al., 2021). Histidine has an effect on the metabolites of various metabolic pathways. L-arginine has a vital role in reducing fat deposition by moderating lipid metabolism (Fouad et al., 2013; Lackner et al., 2022). Taurine affects meat quality by enhancing the anti-oxidative capacity and lipid metabolism, particularly under heat stress (Lu et al., 2019; Xu et al., 2019; Han et al., 2020). Peptidoglycan, coumarins, and some nucleotide biosynthesis/repair pathways are found to be positively associated with the age and growth of the birds, as supported by a few other studies (McFarland and Coon, 2016). As evident from previous studies, peptidoglycan biosynthesis, which is more perfectly enabled by gram-positive than gram-negative bacteria, may also be one cause of the proliferation of piARGs. If a gut has more probiotic bacterial species producing SCFAs such as lactic acid, this will then positively affect the growth by hindering the peptidoglycan biosynthesis in pathogenic bacteria (Lovering et al., 2012; Hidayat et al., 2018; Lee et al., 2022). Furthermore, the coumarins biosynthesis pathway (which increases with age) plays an important role in gut homeostasis. These have antibacterial, antiviral, antifungal, antioxidant, anticancer, and anti-inflammatory activity (El-Far et al., 2016; Duskaev et al., 2021; Sahoo et al., 2021; Cheke et al., 2022). Another important pathway, the tetrapyrrole biosynthesis pathway, that was negatively associated with the age and weight of the broilers was also implicated in earlier studies (Zappa et al., 2010; Plata et al., 2022). On a similar note, foliate transformations and nicotinamide adenine dinucleotide (NAD) biosynthesis are negatively associated with the bird’s age. Similar results regarding the anti-aging effects of NAD are found in other recent studies as well (Johnson and Imai, 2018; Zhang et al., 2020). We have used a predictive functional modeling approach, and there is often a degree of skepticism associated with applying such approaches. However, the majority of our findings were corroborated by published literature, increasing our confidence in the utility of PICRUSt2 to supplement microbial community analyses.

## 5. Conclusion

Through the use of new *in situ* analytical tools, we have demonstrated how certain species persisted as stable components of microbial communities by emphasizing periods where diets remained unchanged. In parallel, we have explored the dynamic components of microbial communities, whether they were positively or negatively associated with the performance parameters of interest, or whether they shot up within a specific narrow range. The microbial community dataset, which originated from 12 pens, each with their own idiosyncrasy, was explicitly modeled in the MicroNiche algorithm to mask out any biases associated with the environments and reveal patterns that lead us to harness their true potential. Overall, our findings suggest a route toward improving the performance of the birds by modulating their microbiome and improving their health by highlighting specific parameters such as diet that are associated with microbial communities’ flux. Considering the content estimation of the different diets and looking at what microbes were promoted by the diet (specificity analyses), we should be able to come up with a dietary intervention to modulate the microbiome to optimize some sort of fitness function. A strong association of microbes with FCR, FI, body weight, and weight gain then enables us to design future intervention strategies where the findings of this study can serve as a reference, as well as help us establish a dietary plan that can help us forgo the use of antibiotic growth promoters for enhancing feed conversion.

## Data availability statement

The raw sequence files supporting the results of this article are available in the European Nucleotide Archive under the project accession number PRJEB25776.

## Ethics statement

The animal study was approved by Agri-Food and Biosciences Institute (AFBI) Establishment License 5002 for AFBI Veterinary Science Division. The study was conducted in accordance with the local legislation and institutional requirements.

## Author contributions

AA: data curation, investigation, methodology, writing—original draft, and writing—review and editing. YC: data curation, investigation, methodology, visualisation, validation, and writing—review and editing. FS: data curation, investigation, methodology, writing—original draft, and writing—review and editing. Uzma: data curation, methodology, visualisation, validation, writing—review and editing, and software. AM: investigation, resources, and funding acquisition. AR: resources, funding acquisition, and project administration. OG and WS: writing—review and editing and funding acquisition. SJ: writing—original draft, writing—review and editing, project administration, and supervision. UI: methodology, software, writing—original draft, writing—review and editing, project administration, supervision, and funding acquisition. All authors contributed to the article and approved the submitted version.

## Funding

AA acknowledges support from the International Research Support Initiative Program of the Higher Education Commission, Pakistan Project No. 1-8/HEC/HRD/2023/12790. UI is funded by the NERC Independent Research Fellowship (NE/L011956/1). UI and WS are further supported by EPSRC (EP/P029329/1 and EP/V030515/1).

## Conflict of interest

AM and AR were employed by Moy Park.

The remaining authors declare that the research was conducted in the absence of any commercial or financial relationships that could be construed as a potential conflict of interest.

## Publisher’s note

All claims expressed in this article are solely those of the authors and do not necessarily represent those of their affiliated organizations, or those of the publisher, the editors and the reviewers. Any product that may be evaluated in this article, or claim that may be made by its manufacturer, is not guaranteed or endorsed by the publisher.

## Footnotes

^1^ https://github.com/gavinmdouglas/q2-picrust2
