# Supplementary material for: Temporal stability and community assembly mechanisms in healthy broiler cecum
Source: Front Microbiol. 2023 Sep 13;14:1197838. doi: 10.3389/fmicb.2023.1197838 (PMC10534011; doi:10.3389/fmicb.2023.1197838)
Supplement: Supplementary file 1 [file Data_Sheet_1.PDF]

eSupplementary Material

Temporal stability and community assembly mechanisms in healthy broiler cecum

Aqsa Ameer[1,†], Youqi Cheng[2,†], Farrukh Saleem[1,†], Uzma[2,†], Aaron McKenna[3], Anne Richmond[3], Ozan Gundogdu[4], William T. Sloan[2], Sundus Javed[1*], and Umer Zeeshan Ijaz[2,5,6*]

[1]Department of Biosciences, COMSATS University Islamabad, Pakistan
[2]Water & Environment Research Group, University of Glasgow, Mazumdar-Shaw Advanced Research Centre, Glasgow, United Kingdom
[3]Moy Park, Armagh, United Kingdom
[4]Department of Infection Biology, Faculty of Infectious and Tropical Diseases, London School of Hygiene and Tropical Medicine, London, United Kingdom
[5]Department of Molecular and Clinical Cancer Medicine, University of Liverpool, Liverpool, United Kingdom
[6]College of Science and Engineering, University of Galway, Ireland

*** Joint Corresponding Authors:**
sundus.javed@comsats.edu.pk
umer.ijaz@glasgow.ac.uk

[†]These authors contributed equally to this work and share first authorship.

1 Statistical analyses

As a pre-processing step, we removed typical contaminants such as *Mitochondria*, and *Chloroplasts*, as well as any Operational Taxonomic Units (OTUs) that were unassigned at all levels, as per recommendations given at https://docs.qiime2.org/2022.8/tutorials/filtering/ and also filtered out samples (<5,000 reads) and singletons giving an abundance table of *n*=337 samples X *P*=2,309 OTUs with sample-wise read statistics as follows: [1st Quartile: 52,682; Median: 86,239; Mean: 93,008; 3rd Quartile: 123,772].

1.1 Complexity-Stability Relationship

The original study (Ijaz et al., 2018) generated temporal microbiome dataset for cecal samples at 12 pens/day and highlighted changes in phylogenetic alpha diversity metrics, particularly Nearest Taxon Index (NTI) and Nearest Relatedness Index (NRI) showing a step response, and creating a window of opportunity for pathogen such as *Campylobacter* to proliferate. This demanded further exploration in terms of complexity and stability relationship, and as a result, we employed the recent framework of (Yonatan et al., 2022). In the past, researchers have modelled the complexity-stability relationship of an ecosystem based on a *Generalised Lotka Volterra model* of the form $\frac{dx_i}{dt} = x_i\left(r_i + \sum_{j=1}^{n} A_{ij}x_j\right)$, where $x_i$is the abundance of species i, r_i is the intrinsic growth rate of species i, and A_{ij} is the interaction coefficient, i.e., the effect of species j on species i. (May, 1972) studied large complex ecosystems, particularly the A_{ij} interaction matrix, where an ecosystem is deemed to be stable if it follows $\alpha\sqrt{nC} < 1$ (also called May's stability criteria), where α^2 and C are the variance and density ("connectance") of the non-zero off-diagonal elements of A_{ij}. Traditionally, the criteria is studied by extracting co-occurrence networks which may not capture the underlying interactions accurately. (Yonatan et al., 2022) gave an alternate criteria (preventing the need to infer A_{ij}) by estimating the *effective connectance* D^2 (where $D^2 \propto \alpha^2 C$) after fitting a regression model to samples overlap in terms of species they share and the sample dissimilarities. We followed the same approach where D^2 was obtained by the slope of regression fitted to the dissimilarity-overlap plot to the 25% top overlap values for the paired-wise dissimilarity/overlap values for q samples where we can calculate $q(q-1)/2$ paired-wise dissimilarity and overlap values. (Yonatan et al., 2022) suggested a minimum q samples to be > 35 to effectively calculate the complexity-stability relationship. Since we have 12 pens/day, in order to satisfy the minimal sample requirement, we have used a moving-window approach, where we have assumed that the samples can be grouped together for 3 (~36 samples), 4 (~48 samples), 5 (~60 samples), 6 (~72 samples), and 7 (~84) days to obtain a temporal complexity-stability profile. For each of these windows, we have calculated D^2 (stability) and n (complexity) using different dissimilarity metrics as per original authors' suggestions, i.e., *Euclidean distance*

(e), *Jensen–Shannon divergence* (rjsd), and *Spearman correlation* (spearman). For calculating n, i.e., the effective number of species, we have used two approaches, the *exponential of Shannon entropy*, i.e., richness, as well as a simpler *binary counting* of the total number of unique species that appear in a particular window. **A high effective connectance D^2 value suggests that a local perturbation in abundance of one or a few taxa is expected to propagate and disrupt the entire microbial community much more substantially than a microbial community with low effective connectance.**

1.2 Antimicrobial Resistance Characterisation

In an approach similar to (Su et al., 2020), we have used KEGG Orthologs (KOs) from PICRUST2 to select a subset of predictive antimicrobial resistance genes (piARGs), with the master list of KEGG KOs to piARGs mapping available at https://www.genome.jp/kegg/annotation/br01600.html, and comprising a total of ~90 KOs primarily beta-lactamase genes. To see how these piARGs vary within a temporal setting (for the three diets, Starter, Grower and Finisher), we calculated how much these genes contributed to beta diversity patterns observed in the dataset. For this purpose, we employed the *Bray-Curtis* (BC) dissimilarity as a metric of community dissimilarity, and is defined as $BC_{jk} = \frac{\sum |X_{ij}-X_{ik}|}{\sum (X_{ij}-X_{ik})}$, where BC is the Bray-Curtis dissimilarity between communities j and k and X is the relative abundance of the KEGG orthologs i. Since BC is a scaled summation of abundance differences between two communities, we can easily partition BC dissimilarity between two samples attributable to the piARGs. To obtain the contribution of piARGs, for two sample, we calculate the BC twice, first calculating the summation in the numerator of the BC expression but using the subset of KOs (BC_subset) relevant to piARGs, and once with all the total KOs (BC_all). There is no change in denominator. Dividing BC_subset by BC_all then reports the fraction of beta diversity attributed to piARGs. For this purpose, we employed R's otuSummary package (S. Yang, 2018). To see if the Bray-Curtis contribution for piARGs was significant between days, we used Tukey Honest Significance Difference (HSD) test from R's Stats package.

1.3 Ensemble Quotient Optimisation

We next wanted to see if we can find a minimal subset of species that either remain stable or changes with respect to a continuous outcome of interest. For this purpose we incorporated the Ensemble Quotient Optimisation (EQO) approach of (Shan et al., 2022). The approach uses a relative abundance table, called community matrix $\boldsymbol{M}$ (P OTUs over n samples), where the goal is to obtain a vector $\boldsymbol{x} \in (0,1)^P$ where the ith position in the vector is either 0 or 1, i.e., a subset of species with values 1 belong to an ensemble which we are interested in recovering. This ensemble is recovered in the context of a phenotype/predictor variable $\boldsymbol{y}$ by optimizing an *Ensemble Quotient* $EQ = \frac{\boldsymbol{x}^T\boldsymbol{Qx}}{\boldsymbol{x}^T\boldsymbol{Px}}$, through a genetic algorithm (an optimization algorithm), where $\boldsymbol{P}$ and $\boldsymbol{Q}$ are algebraic transformations of the community matrix that capture the covariance between species, and the covariance between species and $\boldsymbol{y}$. The choice of $\boldsymbol{y}$ dictates what ensemble we recover, and can be used in two cases: a) If the interest lies in an ensemble of species *that remain stable* for a set of samples, then $\boldsymbol{y}$ is considered uniform i.e., consisting of 1s, with $\boldsymbol{Q} = \boldsymbol{M}^T\boldsymbol{1}\boldsymbol{1}^T\boldsymbol{M}$, and $\boldsymbol{P} = \boldsymbol{M}^T\boldsymbol{M} - \frac{2}{n}\boldsymbol{M}^T\boldsymbol{1}\boldsymbol{1}^T\boldsymbol{M} + \frac{1}{n^2}\boldsymbol{M}^T\boldsymbol{1}\boldsymbol{1}^T\boldsymbol{1}\boldsymbol{1}^T\boldsymbol{M}$; b) If the interest lies in an ensemble of species whose cumulative abundance correlates with a continuous physico-chemical parameter y, then we optimize the algorithm with $\boldsymbol{Q} = \boldsymbol{M}_0^T\boldsymbol{y}_0\boldsymbol{y}_0^T\boldsymbol{M}_0$, $\boldsymbol{P} = \boldsymbol{M}_0^T\boldsymbol{M}_0$ ($\boldsymbol{M}_0$ is the centered community matrix $\boldsymbol{M}$ whose column means are zero with $\boldsymbol{y}_0$ also a centered version of $\boldsymbol{y}$). Within the context of temporal data, we have used the case (a) to see which subset microbes do not change over the whole time span (quality of fit is returned as Coefficient of Variation CV), whilst, case (b) was used to see which subset of microbes have a relationship with the performance parameters (quality of fit is returned as a correlation coefficient between the continuous outcome and the cumulative abundance of the ensemble), or *Day*s, including mean *Body Weight* (BW_Mean), *Feed Conversation Ratio* (FCR), *Feed Intake* (FI), and *Weight Gain* (Gain), as used in the original publication (Ijaz et al., 2018). To optimize the EQ to obtain $\boldsymbol{x}$, we followed the genetic algorithm optimization located at https://github.com/Xiaoyu2425/Ensemble-Quotient-Optimization. In the genetic algorithm, we have used the following parameterizations: a population size of 100 solutions, maximum of 500 generations, and maximum 30 taxa to be returned as an ensemble.

1.4 Generalised Linear Latent Variable Model.

To find the relationship between individual microbial genera/genes and all the sources of variation [covariates such as Pens, diet type (Starter, Grower, and Finisher), Days, BW_Mean, FCR, FI, and Gain], we have used *Generalised Linear Latent Variable Model* (GLLVM) (Niku et al., 2019), which extends the basic generalized linear model that regresses the mean abundances μ_{ij} (for i-th sample and j-th genus/gene) against the covariates x_i as above by incorporating latent variables u_i as $g(\mu_{ij}) = \eta_{ij} = \alpha_i + \beta_{0j} + \boldsymbol{x}_i^T\boldsymbol{\beta}_j + \boldsymbol{u}_i^T\boldsymbol{\theta}_j$, where $\boldsymbol{\beta}_j$ are the genus specific

coefficients associated with individual covariate (a 95% confidence interval of these whether positive or negative, and not crossing 0 boundary gives directionality with the interpretation that an increase or decrease in that particular covariate causes an increase or decrease in the abundance of the genus/gene), and $\boldsymbol{\theta}_j$ are the corresponding coefficients associated with latent variable. β_{0j} are genus-specific intercepts, whilst α_i are optional sample effects which can either be chosen as fixed effects or random effects.

To model the distribution of individual genera or gene, we have used *Negative Binomial distribution*. Additionally, the approximation to the log-likelihood is done through *Variational Approximation* (VA) with final sets of parameters in glvmm() function being family = 'negative.binomial', and method="VA. For convergence of GLLVM algorithm, we have used control.start=list (n.init = 7, jitter.var = 0.1) for genera, and control.start=list (n.init = 5, jitter.var = 0.1) as well as starting.val="random" for genes. This, we did for top 100 most abundant genera in our datasets, and also on a subset of KEGG KOs recovered from PICRUSt2, particularly, those tagged as piARGs. Our additional interest in using the GLLVM algorithm is to obtain co-occurrence relationships between the respective genera and the genes, as the residual covariance matrix $\boldsymbol{\Sigma} = \boldsymbol{\Gamma}\boldsymbol{\Gamma}^T$ for the latent variables, where $\boldsymbol{\Gamma} = [\theta_1 \ldots \theta_m]$ for *m* latent variables, and gives the dependency/interactions between genera/genes after accounting for the covariates considered in the model.

1.5 CODA LASSO Model

Whilst the GLLVM uses a single genus or a gene as a focal point, we also wanted to see if we can use individual performance parameter/days as dependent variables and find the minimal set of genera or pathways that associate with them. The CODA LASSO (Susin et al., 2020) regression model is similar to GLLVM, and is of the form $y_i = \beta_0 + \beta_1 \log(x_{1i}) + \cdots + \beta_j \log(x_{ji}) + \epsilon_i$ (for i-th sample and j-th feature, with x_{ji} being the abundance of genera or a MetaCyc pathway returned from PICRUSt2), and where the outcome y_i can be taken as either *Days*, *BW_Mean*, *FCR*, *FI*, and *Gain*. The model uses two constraints: a) $\sum_{k\geq 1} \beta_k = 0$ (i.e., all β-coefficients sum up to 1) which makes the algorithm invariant by returning two disjoint sets of features in a log contrast fashion; and b) the optimization function incorporates a LASSO shrinkage term $\lambda \sum_{k\geq 1}|\beta_k|$ as $\sum_{i=1}^{n}(y_i - \beta_0 - \beta_1 \log(x_{1i}) - \cdots - \beta_j \log(x_{ji}))^2 + \lambda \sum_{k\geq 1}|\beta_k|$ subject to $\sum_{k\geq 1} \beta_k = 0$. Here, λ is the penalization parameter, and forces some of the $\boldsymbol{\beta}$-coefficients to go zero, particularly those that do not have a relationship with the genera/pathways and serves as a means to do variable selection.We have used coda_glmnet() function from R's coda4microbiome package (Calle & Susin, 2022). We have used the top 100 most abundant genera in the CODA-LASSO model whilst all the MetaCyc pathways returned from PICRUSt2.

1.6 Microbial Niche Breadth, Overlap, and Specificity

We next adopted a taxa-centric approach, where our emphasis was to highlight taxa that hold importance in a temporal (Days) or spatial (Pens) context. In ecology, one of the important method is to assess what niche species occupy, and whether there is a degree of overlap between them. For such an assessment, it is important to consider all possible sets of environments (dictated by biotic or abiotic variations), with the total number of environments as a parameter in the model. Since we have 12 pens, our assumption is that each pen by virtue of its location will preserve the environmental conditions, with the layout given in Supplementary Figure 1.

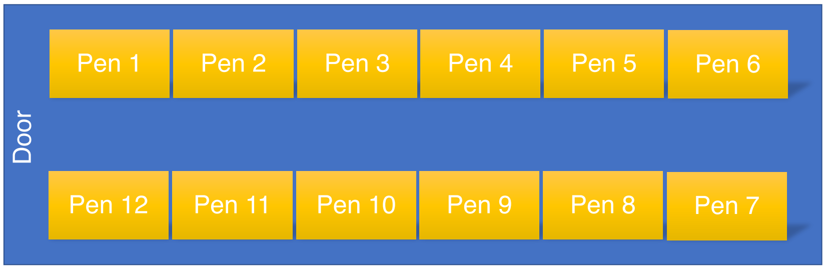

Supplementary Figure 1. Schematic layout of pens used in the study.

To identify the roles of microbes within the context of all these pens, we have used the R's MicroNiche package (Finn et al., 2020). The aim is to determine *generalist* (that should exist in majority of the pens) and *specialist* (that should exist in some pens) microbial species as well as pen-dependent positive/negative association of microbial species with covariates such as *Days*, *BW_Mean*, *FCR*, *FI*, and *Gain*.

Before, applying these approaches, we filtered out genera by using the limit of quantification (LOQ) approach as per author's instruction. Briefly, LOQ filters out microbes that fall below a "decision boundary", calculated from the distribution of microbes with 95% certainty that these microbes will fall within a null distribution where the mean microbial abundance is zero. To calculate the standard deviation of the null distribution, the lognormal rank distribution of the microbes with the dataset was fitted with $S(R) = S_0 e^{-a^2 R^2}$ where log abundance of microbe S at rank R is dependent on coefficient a and rank R calculated as $a = \sqrt{\frac{\ln S_0}{S_m}/R^2}$ where S_m is the lowest taxon abundance of S. To calculate LOQ, we fit the above log normal model to data, and LOQ is then determined as the overlap between the null hypothesis (i.e., a microbe's mean abundance is zero) and where the microbe falls within 1 standard deviation of the above model.

After filtering out the genera, we then calculated the niche breadth as Levins' $B_N = \frac{1}{R}\sum_{i=1} p_i^2$, where p_i is the proportional abundance of a genus in the i-th pen, with total number of pens (environments) being R (12 in this case). If B_N approaches 1 for a given genus, then it is considered as a "generalist", whilst if it approaches $1/R$, then it can be tagged as a "specialist". To derive the p-value for Levins' B_N i.e., if it we can call a genus a generalist or a specialist with great certainty, a null modelling approach is used, where a random normal distribution of 999 possible niche breadths were produced for a genus, and allows a p-value to be assigned depending on whether a genus's B_N is greater or lower than the mean of the null model. As per author's recommendation, after applying null modelling, the 5th Quantile and 95th Quantile were obtained to tag the genera as specialist if its B_N < 5th Quantile, and generalist, if its B_N > 95th Quantile. Those that fell in the inter-range were tagged as undecided.

In the second step, we then calculated the overlap of these undecided/specialist/generalists using Levins' Overlap formula $\text{LO}_{i,j} = \frac{\sum_{i,j=1}(p_{ir})(p_{jr})}{\sum_{i=1}(p_{ir}^2)}$, where p_i is the proportional abundance of genus i in the r-th pen, and p_j is the abundance of genus j in the r-th pen, where i and j were selected after tagging an individual genus as undecided, specialist or generalist.

In addition to Levins' B_N, we also calculated Hurlbert's $B_N = \frac{1}{\sum_{i=1}\frac{p_i^2}{r_i}}$, where we have an additional r_i proportional covariate data (*Days*, *BW_Mean*, *FCR*, *FI*, and *Gain*) in the formula. The model yields a value between 0 and 1 for each genus and corresponding covariate, indicating whether there is an inverse (~0) or a positive relationship (~1), with 0.5 indicating no relationship to the covariate. Similar to Levins' B_N approach, a null modelling procedure was considered by generating a random normal distribution of 999 possible niche breadth, and by tagging it as "negative" if its B_N < 5th Quantile, and "positive", if its B_N > 95th Quantile. To determine positive and negative relationship (potentially symbiosis and antagonism) between the genera, we have used Proportional Overlap formula $\text{PO}_{i,j} = 1 - \left(\frac{X \cap Y}{X \cup Y}\right)$, where *X* (for genus *i*) and *Y* (for genus *j*) are the Feinsinger's PS, calculated as $\text{PS} = 1 - 0.5\sum_{i=1}|p_i - r_i|$, and is similar to Hurlbert's B_N. The Proportional Overlap $\text{PO}_{i,j}$ is a Jaccard similarity coefficient which approaches 0 for genus pairs that are inversely related to each other and approaches 1 for genus pairs that are positively related to each other. Note that we have calculated the $\text{PO}_{i,j}$ for those genera that were identified as undecided/positive/negative after applying Hurlbert's B_N.

Next, we wanted to explore if certain genera exist within a narrow range of covariates considered in this study (*Days*, *BW_Mean*, *FCR*, *FI*, and *Gain*). This is particularly important on temporal basis, as the diet changed from Starter (Day 0 to 10) to Grower (Day 11 to 25) to Finisher (Day 26 onwards), and the diets themselves could potentially select for certain genera. For this purpose we have used R's Specificity Package (Darcy et al., 2022) that calculates Rao's Quadratic Entropy (RQE) as $RQE = \sum_{i=1}^{s-1}\sum_{j=i+1}^{s} D_{ij} p_i p_j$ where genus abundance $p_i p_j$ is the multiplication of the abundance of a specific genus in samples i and j, respectively, weighted by the difference in the covariate value (*Days*, *BW_Mean*, *FCR*, *FI*, and *Gain*) D_{ij}. A null modelling procedure is then applied (statistical effect size) where 999 random permutations were obtained for the abundance table, and RQE values were then obtained for these random permutations. Deviation of the original RQE from the average of RQEs of these random permutations then gives a "Spec" number, ranging from -1 to +1, with 0 as the null hypothesis that the genus weights are randomly ordered with regard to sample identity, with perfect *specificity* when Spec approaches -1 and perfect *cosmopolitanism* when spec approaches +1, and with the null modelling procedure providing additional p-values for significance. For visualization purposes, we have only plotted the lowest 25th quartile (i.e., those genera that were specific).

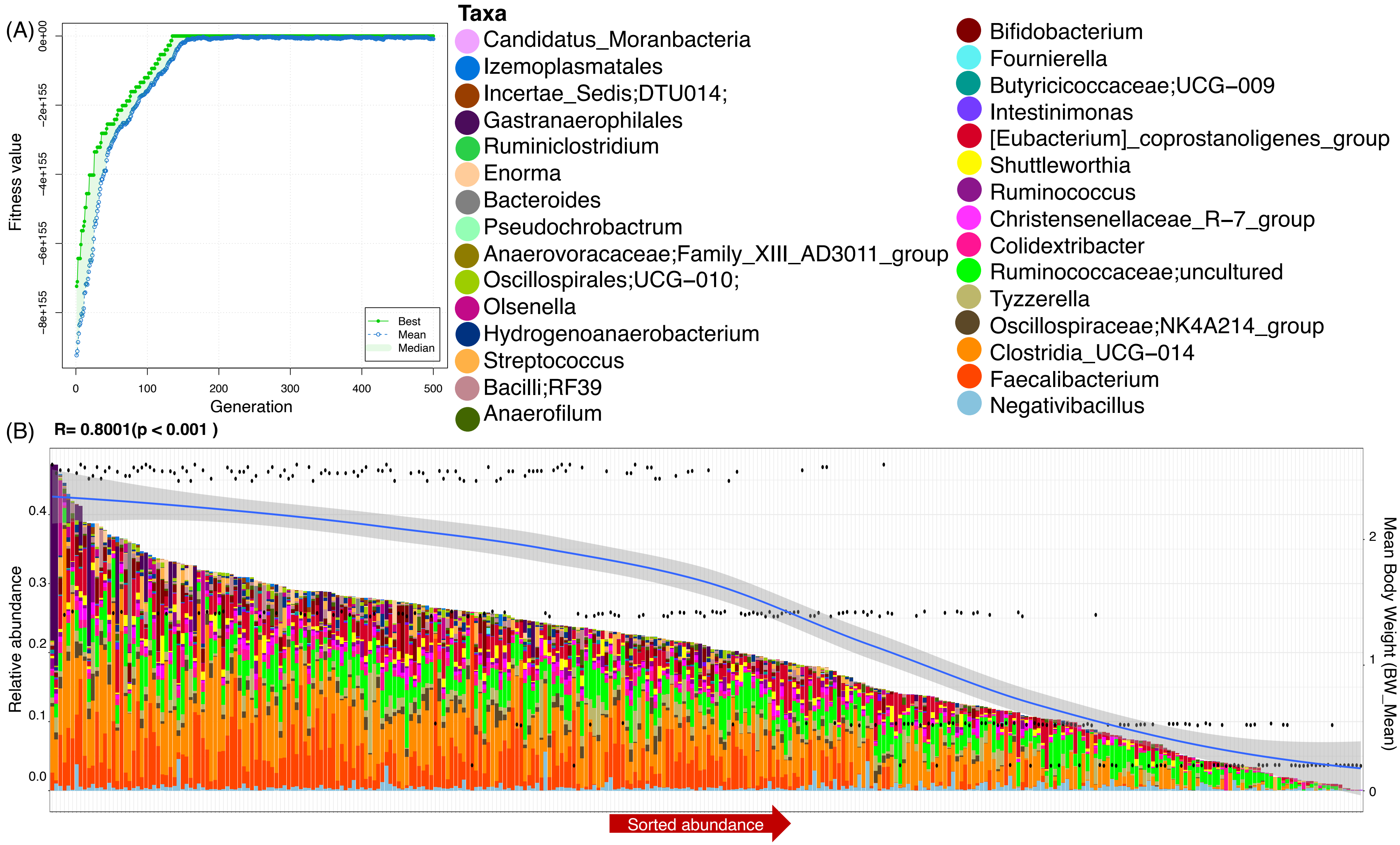

Supplementary Figure 2: Ensemble (minimal subset of genera) returned after applying Ensemble Quotient Optimisation (EQO) technique using BW_mean as a continuous predictor. (A) shows the fitness value evolution of the genetic algorithm in finding these ensembles highlighting the convergence to a steady state solution, and (B) shows the sorted abundance of the ensembles with biplot of BW_mean smooth values (right y-axis) and showing correlation value between the ensemble and BW_Mean just above the plot. This was performed for all the samples.

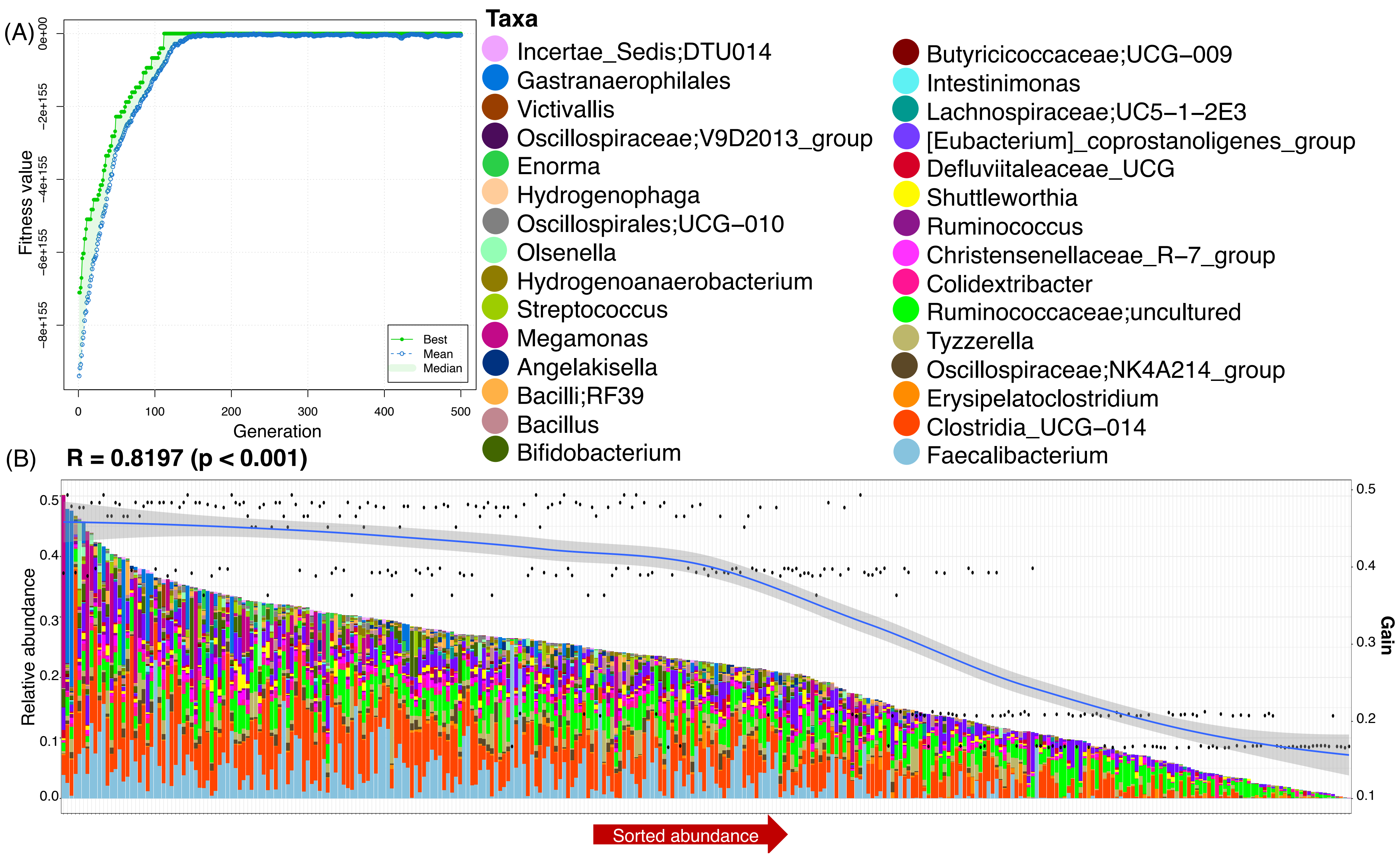

Supplementary Figure 3: Ensemble returned after applying EQO to all samples using Gain as a predictor with the description similar to what is provided in the legend of Supplementary Figure 2.

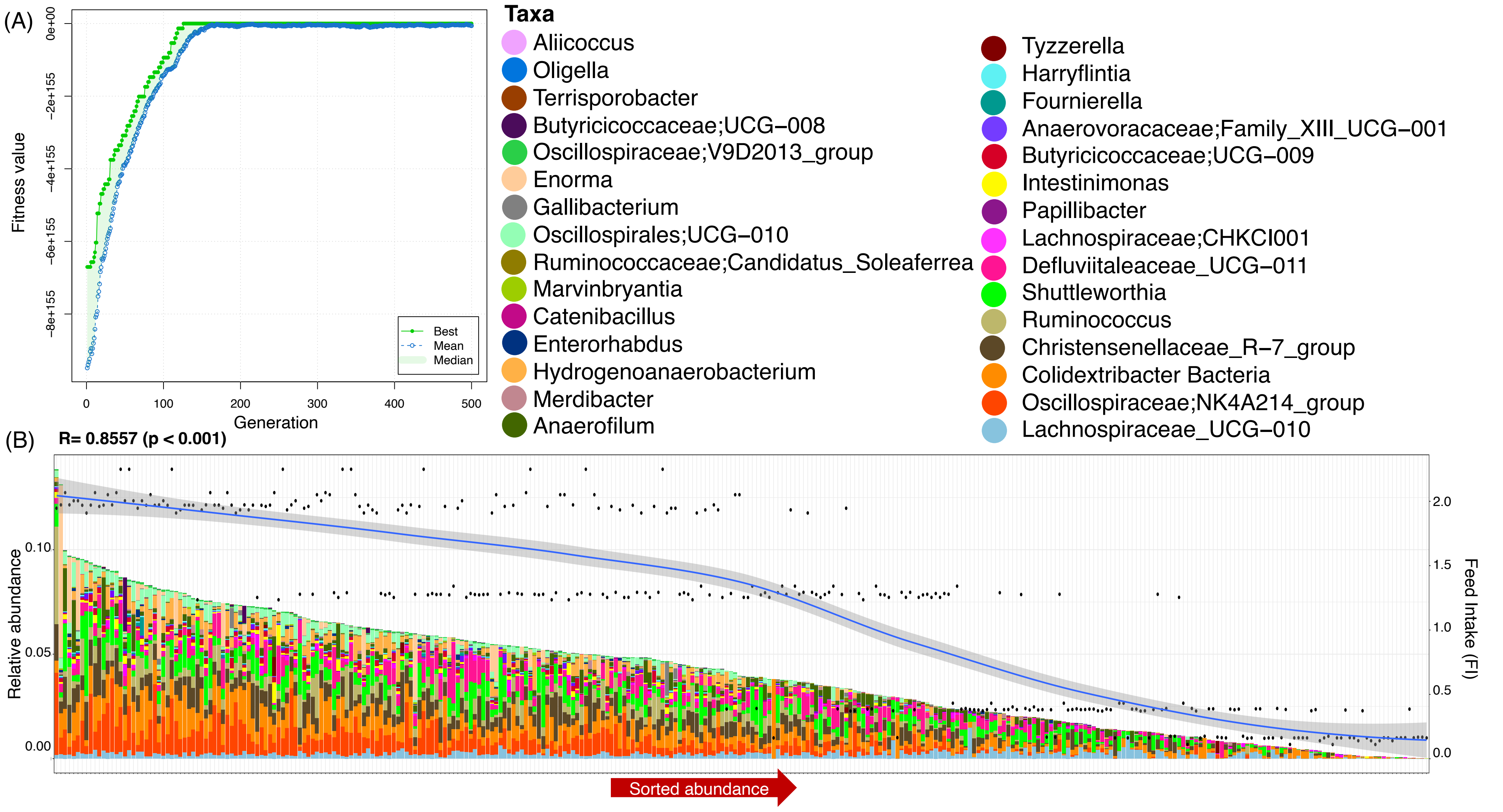

Supplementary Figure 4: Ensemble returned after applying EQO to all samples using Feed Intake (FI) as a predictor with the description similar to what is provided in the legend of Supplementary Figure 2.

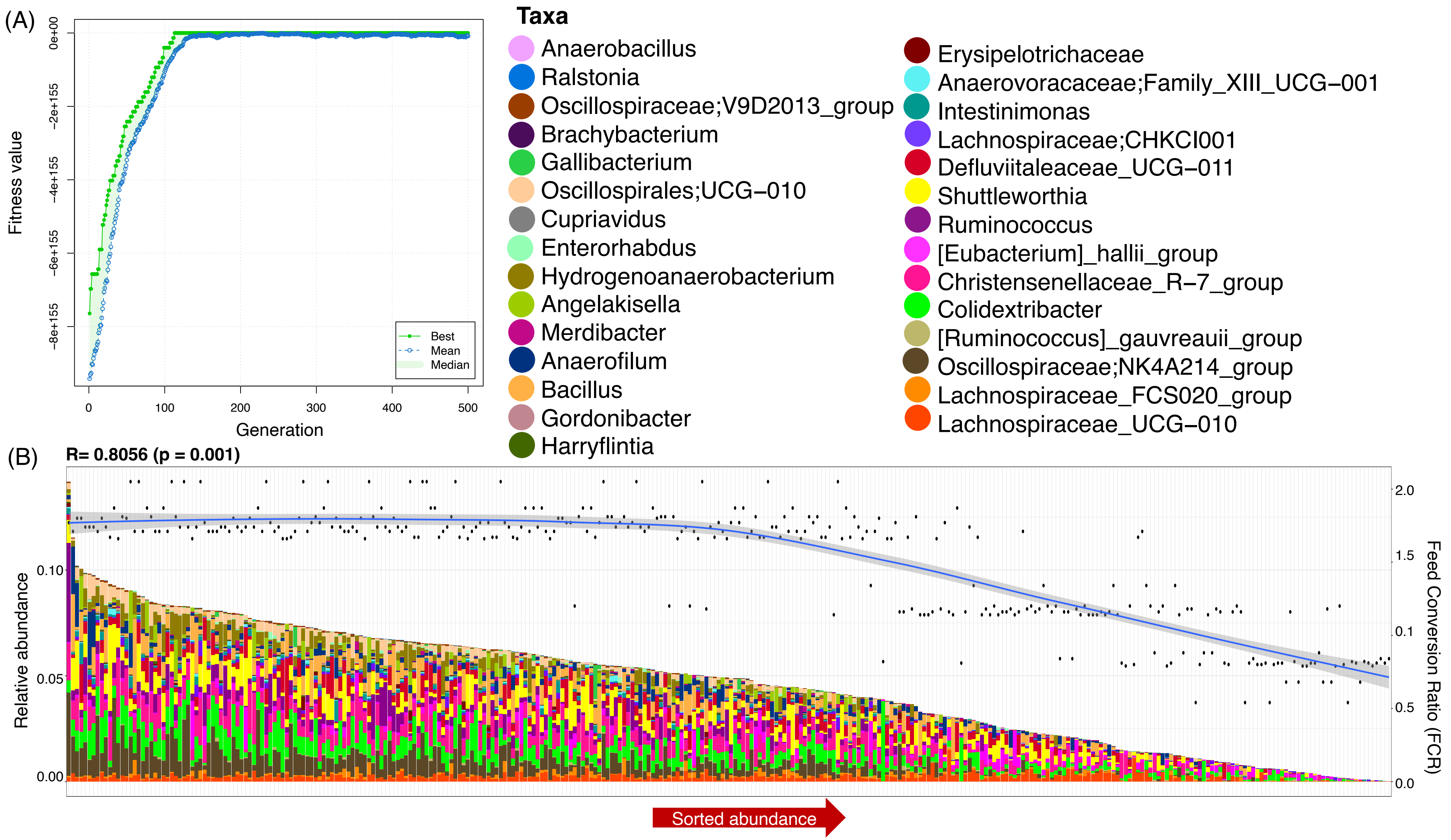

Supplementary Figure 5: Ensemble returned after applying EQO to all samples using Feed Conversion Ratio (FCR) as a predictor with the description similar to what is provided in the legend of Supplementary Figure 2.

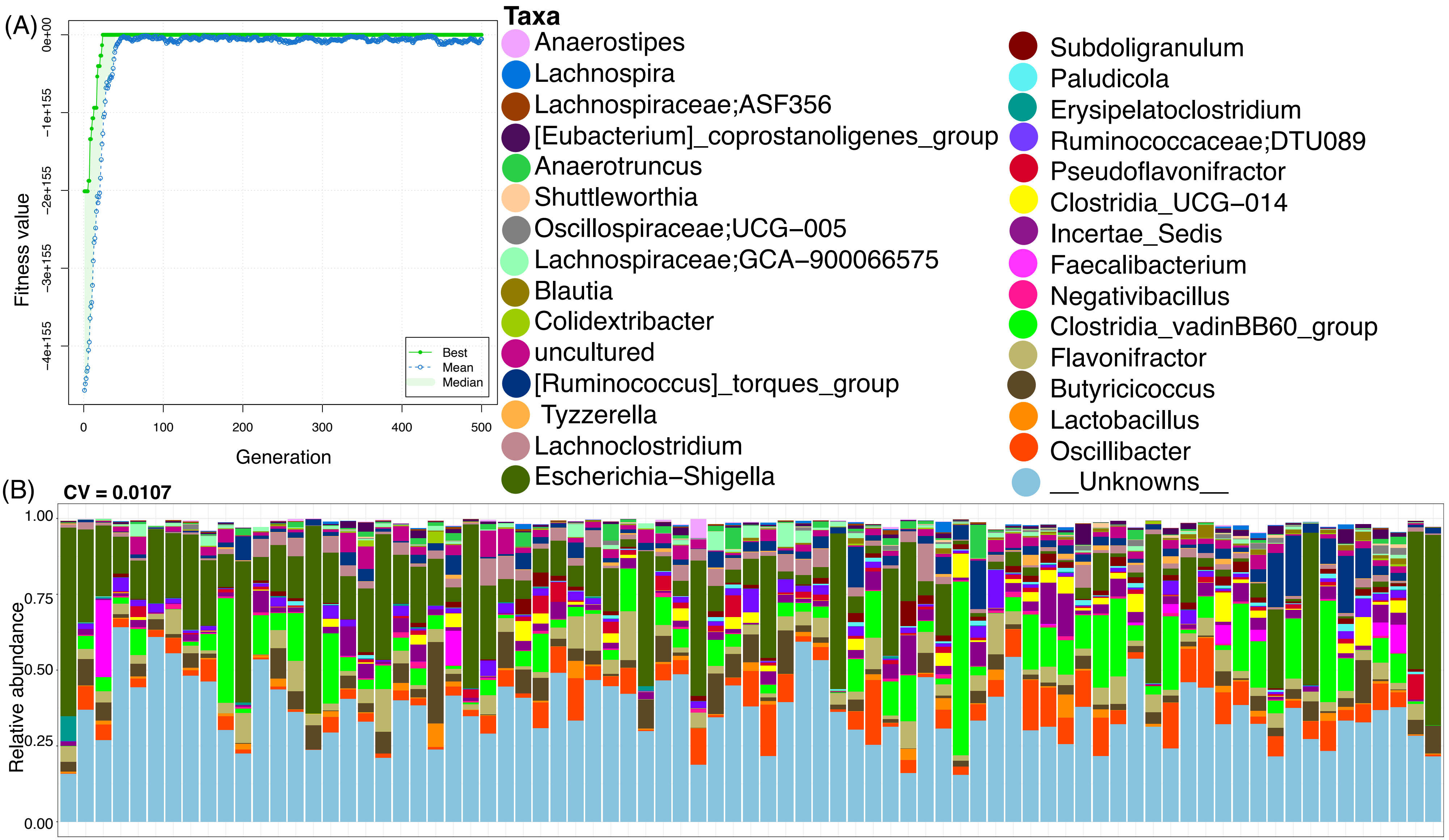

Supplementary Figure 6: Stable ensemble returned after running EQO algorithm in uniform phenotypic variable mode with (A) showing the fitness value evolution of the genetic algorithm in finding these ensembles highlighting the convergence to a steady state solution, and (B) showing the relative abundance profiles with *Coefficient of Variation* (CV) values given on the top of the plot. The lower CV value signifies higher stability. Here, we have only used the samples when the starter diet was given to the broiler

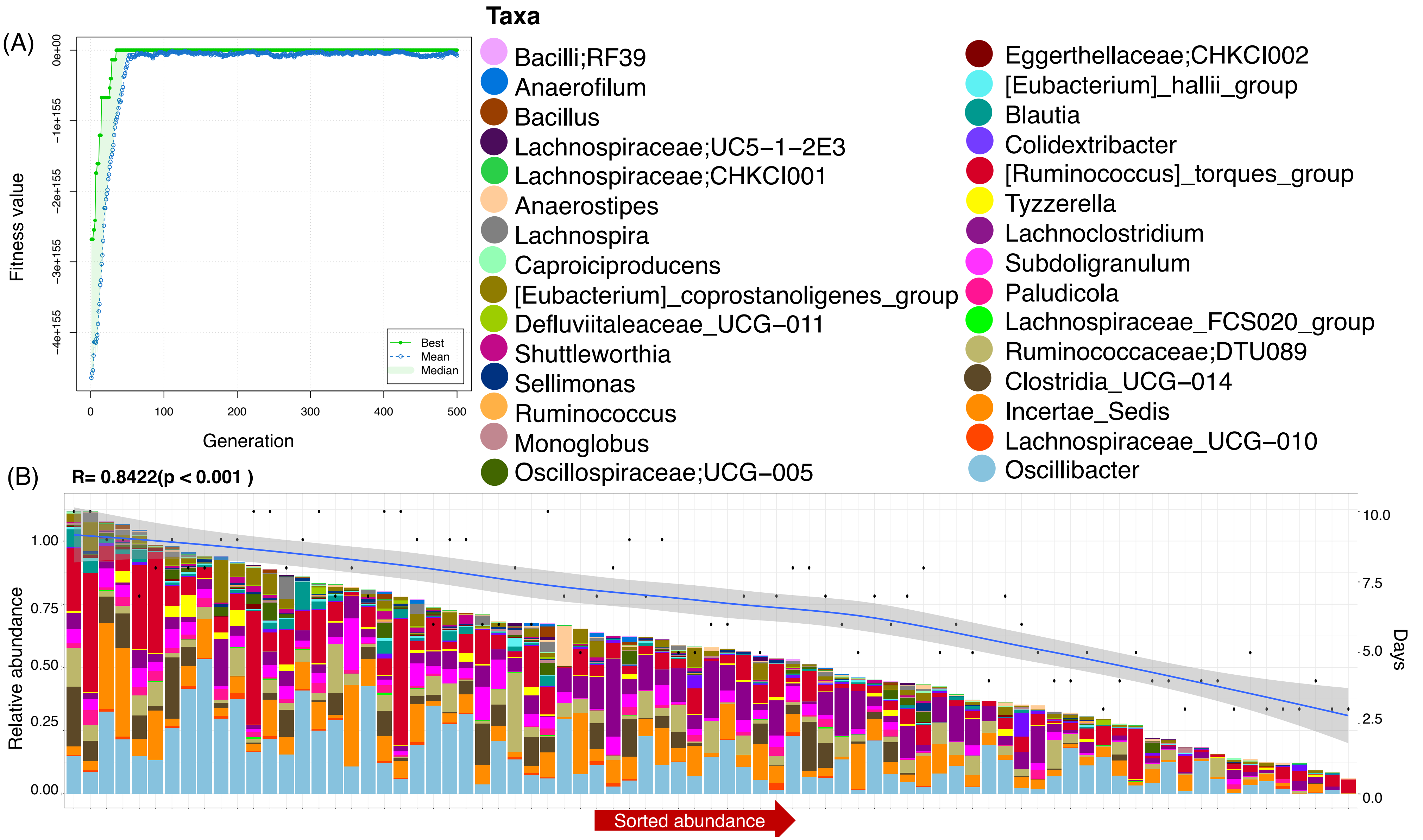

Supplementary Figure 7: Ensemble returned after applying EQO to all samples using *Day* as a predictor with the description similar to what is provided in the legend of Supplementary Figure 2. Here, we have only used the samples when the starter diet was given to the broiler

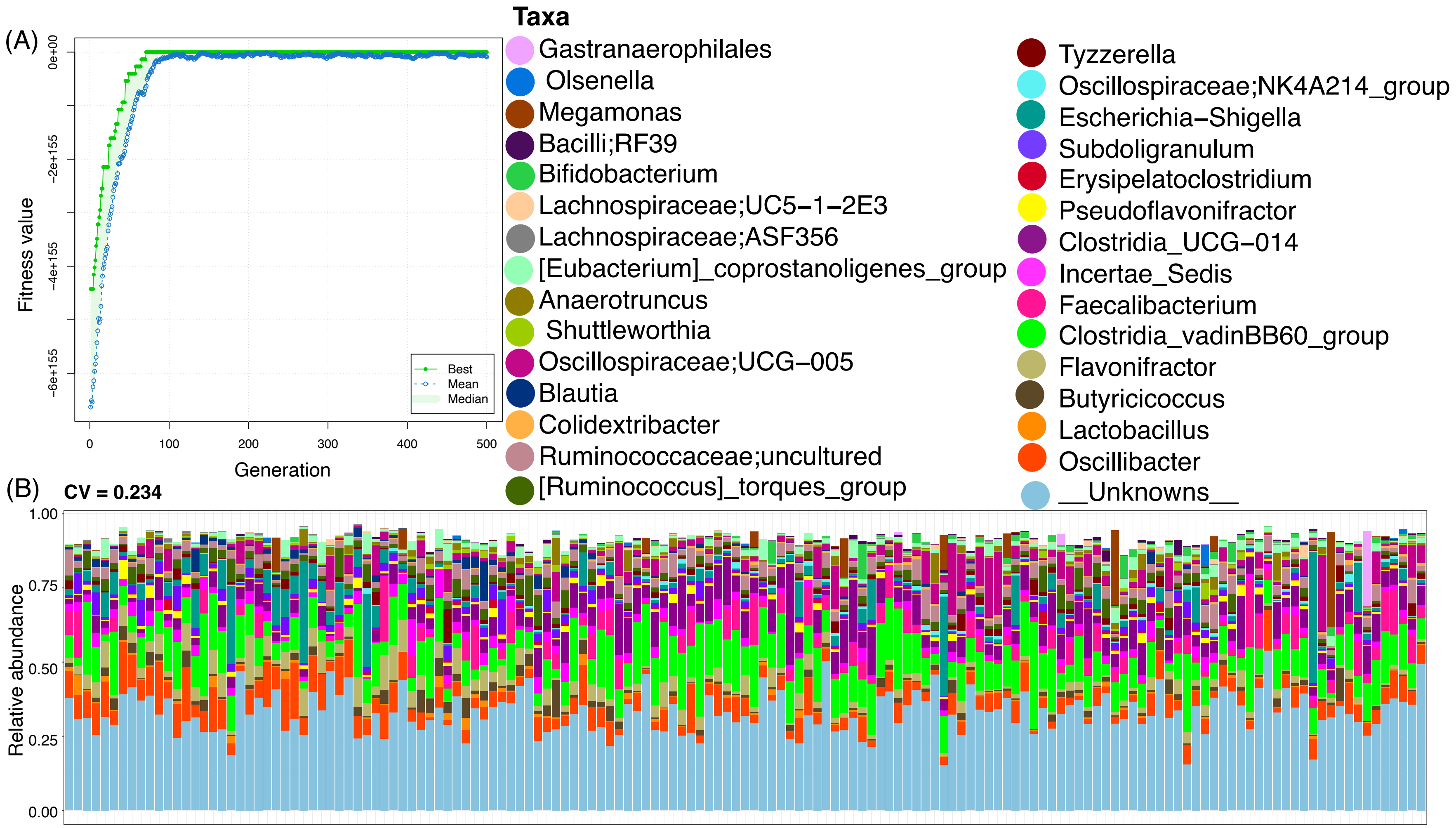

Supplementary Figure 8: Stable ensemble returned after applying EQO to the samples when the grower diet was given with the description similar to what is provided in the legend of Supplementary Figure 6.

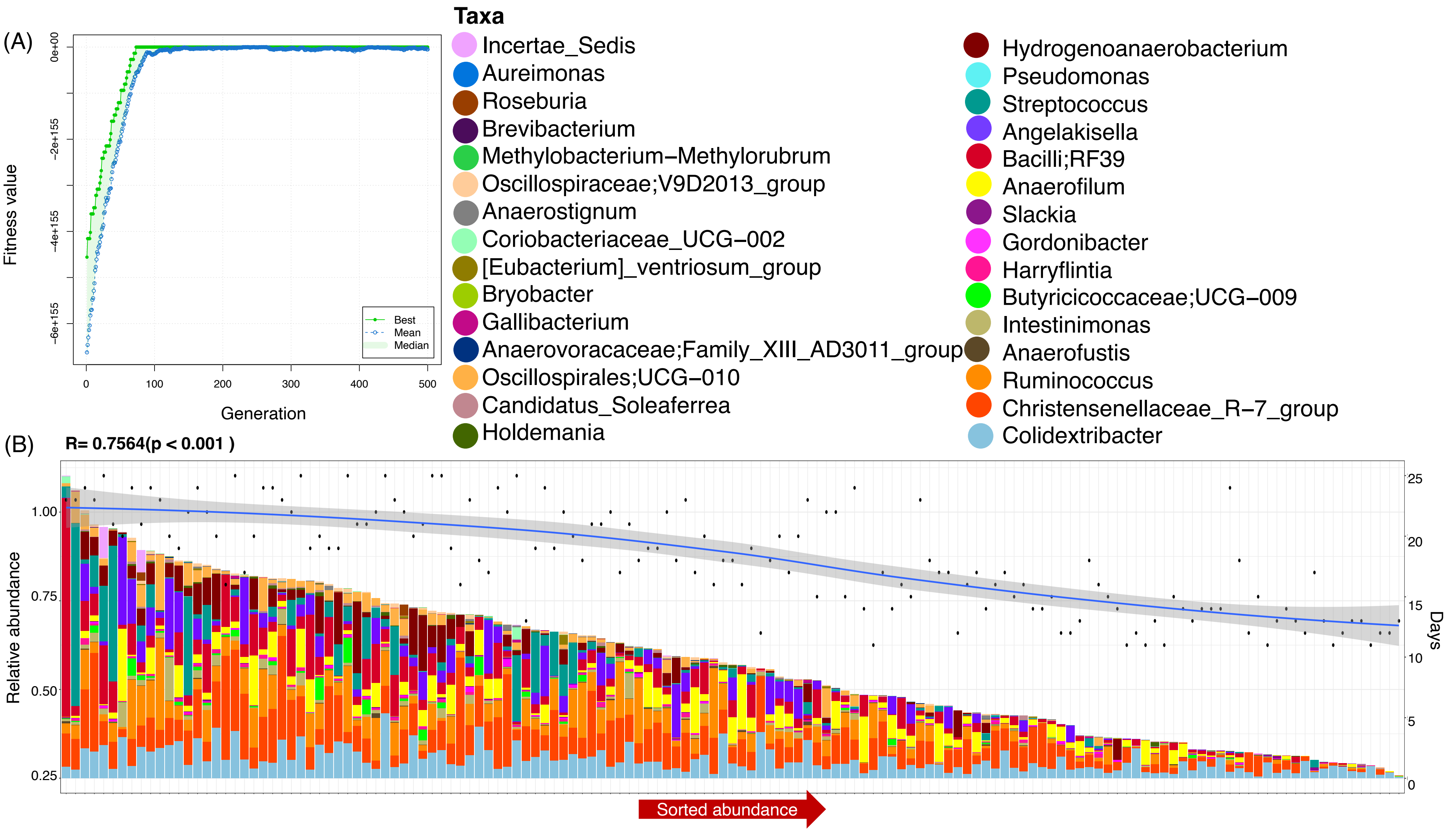

Supplementary Figure 9: Ensemble returned after applying EQO to all samples using *Day* as a predictor with the description similar to what is provided in the legend of Supplementary Figure 2. Here, we have only used the samples when the grower diet was given to the broiler

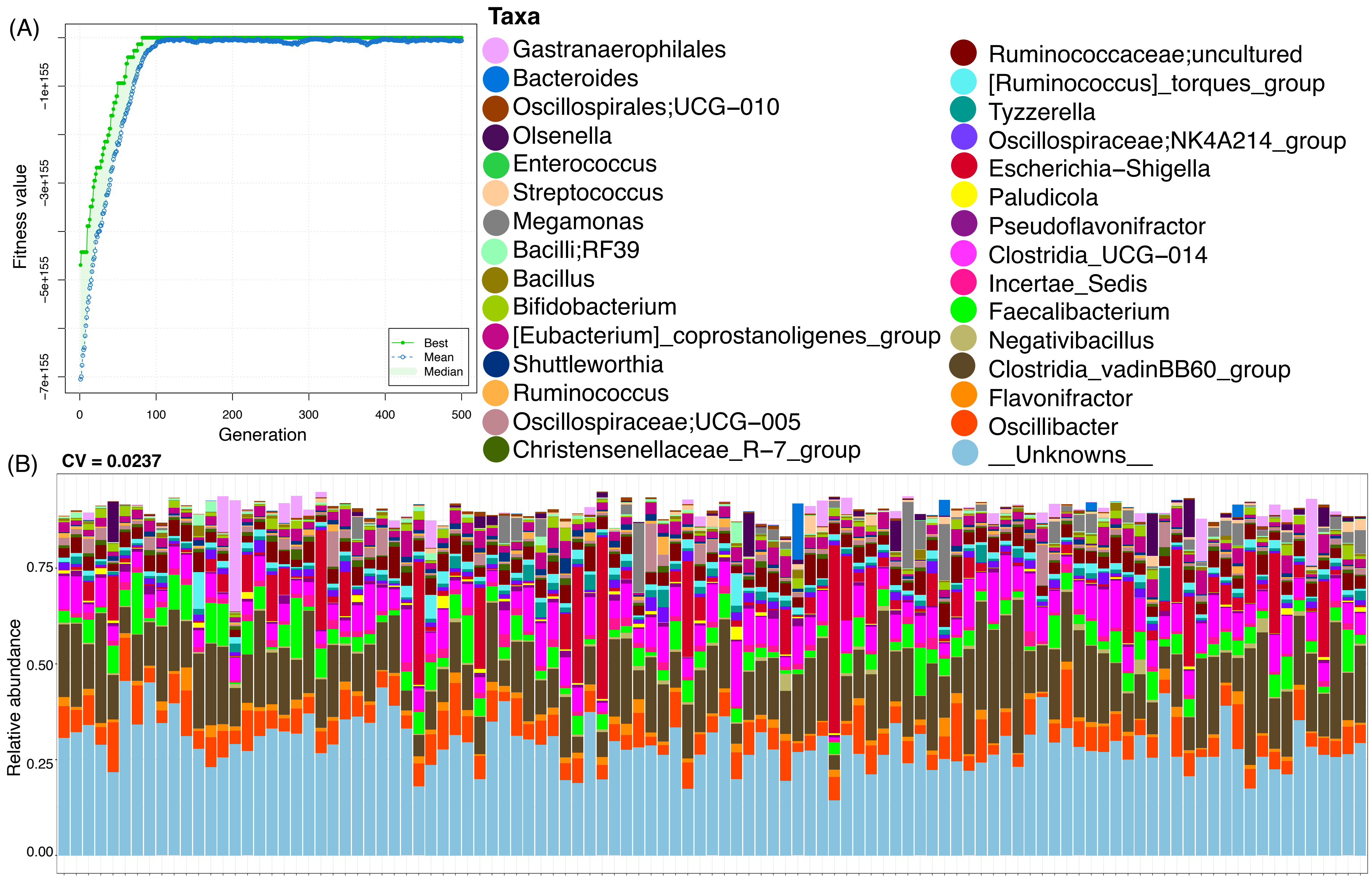

Supplementary Figure 10: Stable ensemble returned after applying EQO to the samples when the finisher diet was given with the description similar to what is provided in the legend of Supplementary Figure 6.

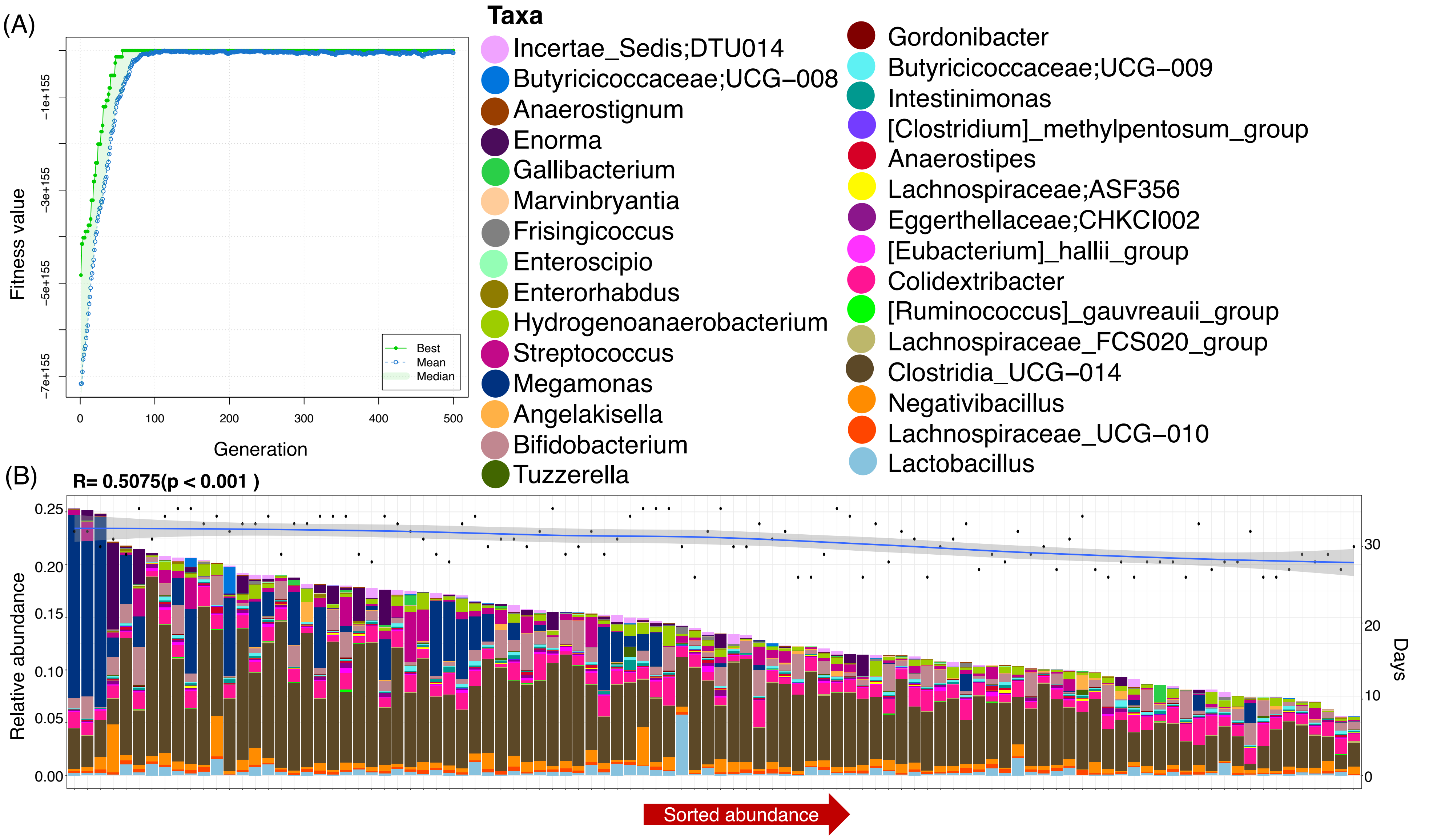

Supplementary Figure 11: Ensemble returned after applying EQO to all samples using *Day* as a predictor with the description similar to what is provided in the legend of Supplementary Figure 2. Here, we have only used the samples when the finisher diet was given to the broiler

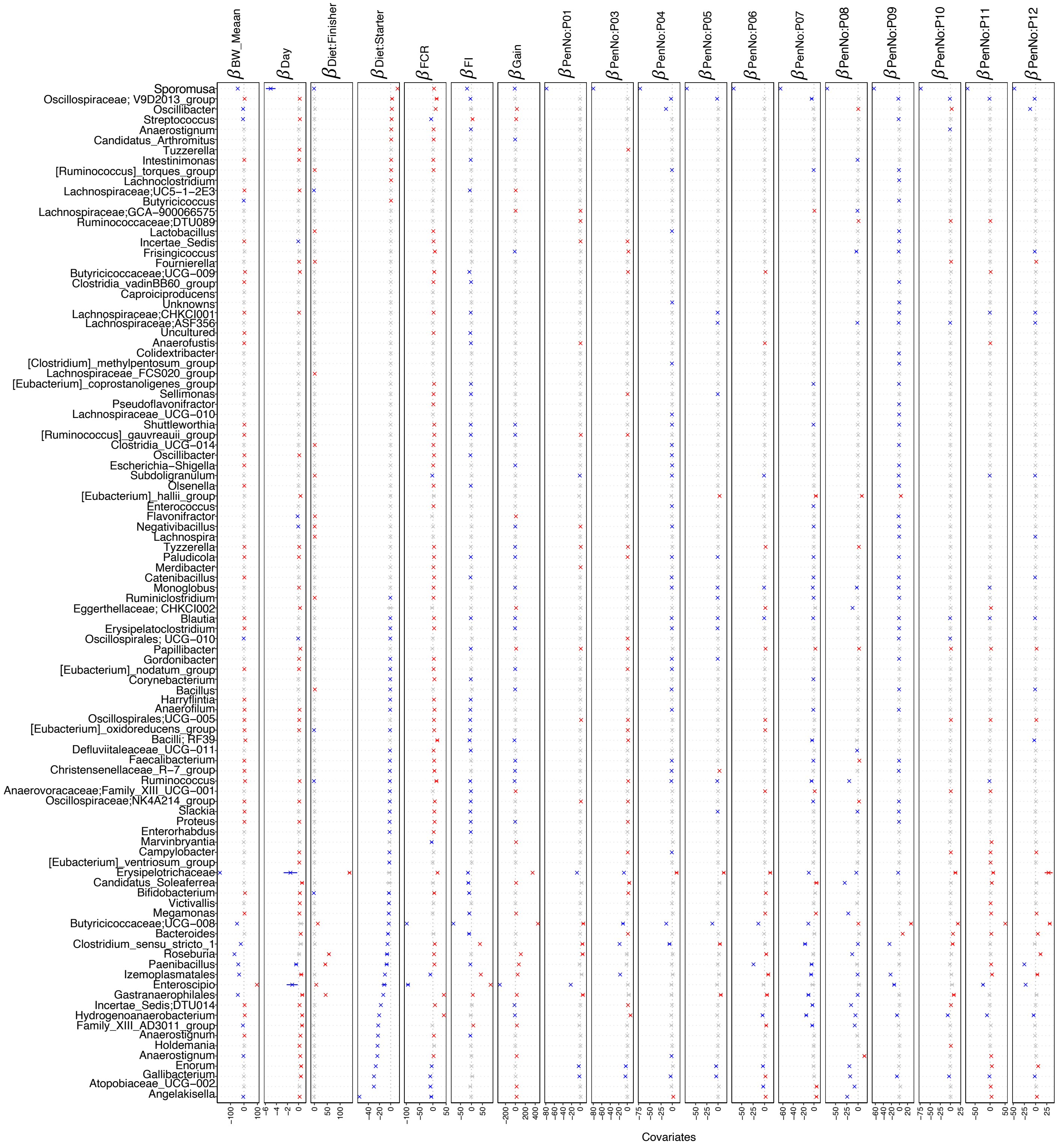

Supplementary Figure 12: β −coefficients returned from GLLVM procedure in Figure 4B with Diet:Grower and PenNo:P02 as references.

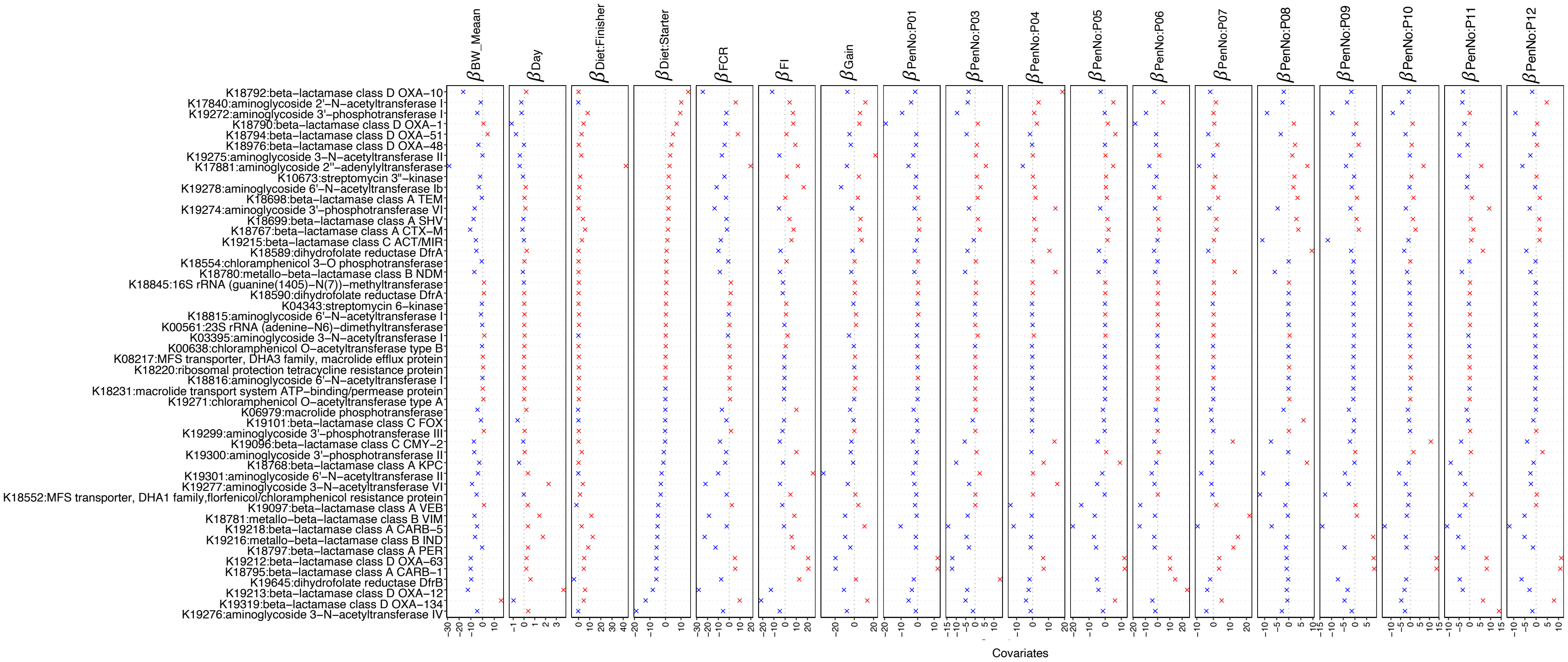

Supplementary Figure 13: β −coefficients returned from GLLVM procedure in Figure 5B with Diet:Grower and PenNo:P02 as references.

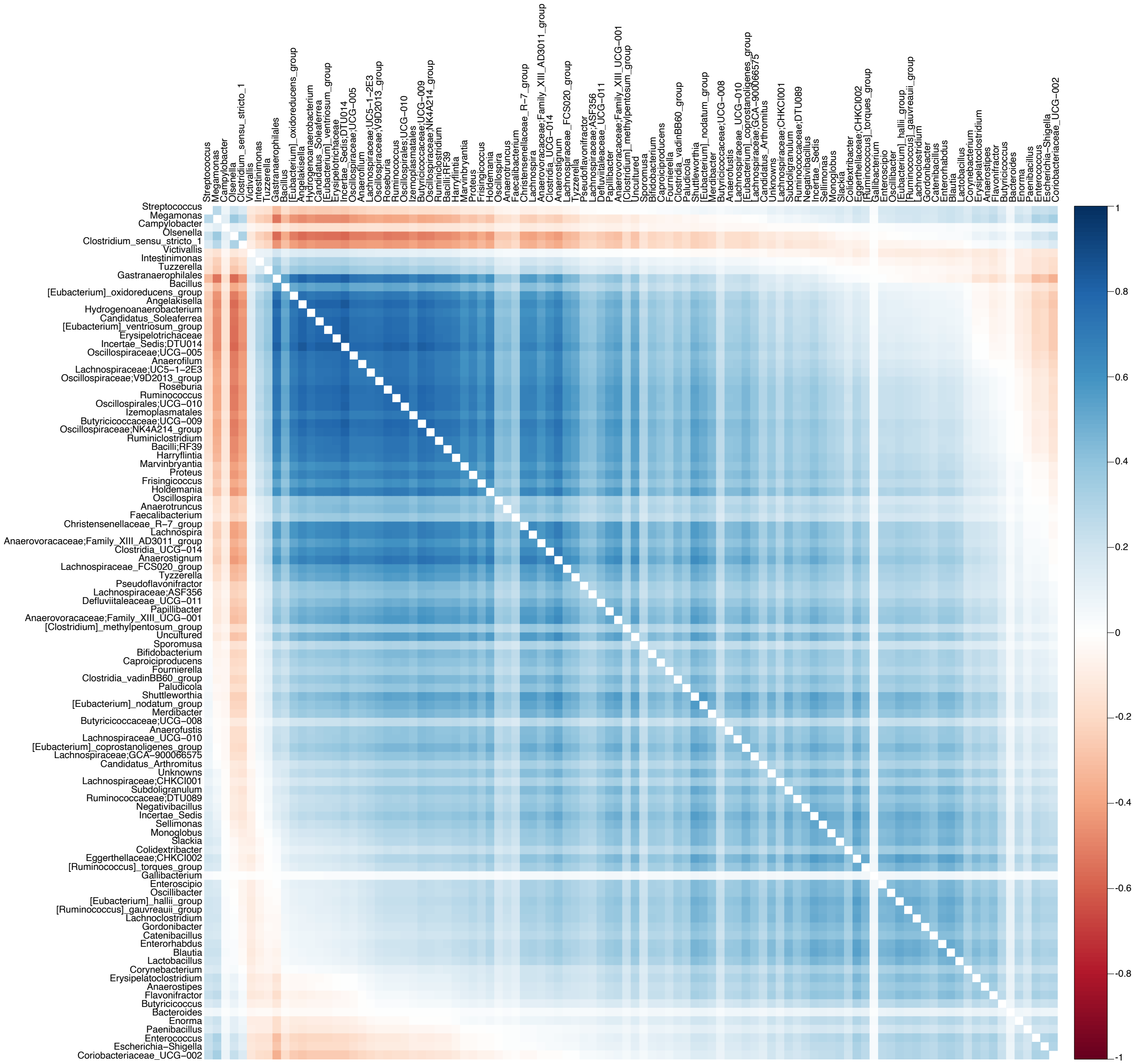

Supplementary Figure 14: Co-occurrence relationship between genera recovered from the residual covariance matrix ***Σ*** that are not explained by the observed covariates in the GLLVM model in Figure 4A with Diet:Starter and PenNo:P01 as references. Here, blue represent the positive correlation (taxa 1 increasing in abundance leads to taxa 2 increasing in abundance), and red represent the negative relationship (taxa 1 increasing in abundance leads to taxa 2 decreasing in abundance).

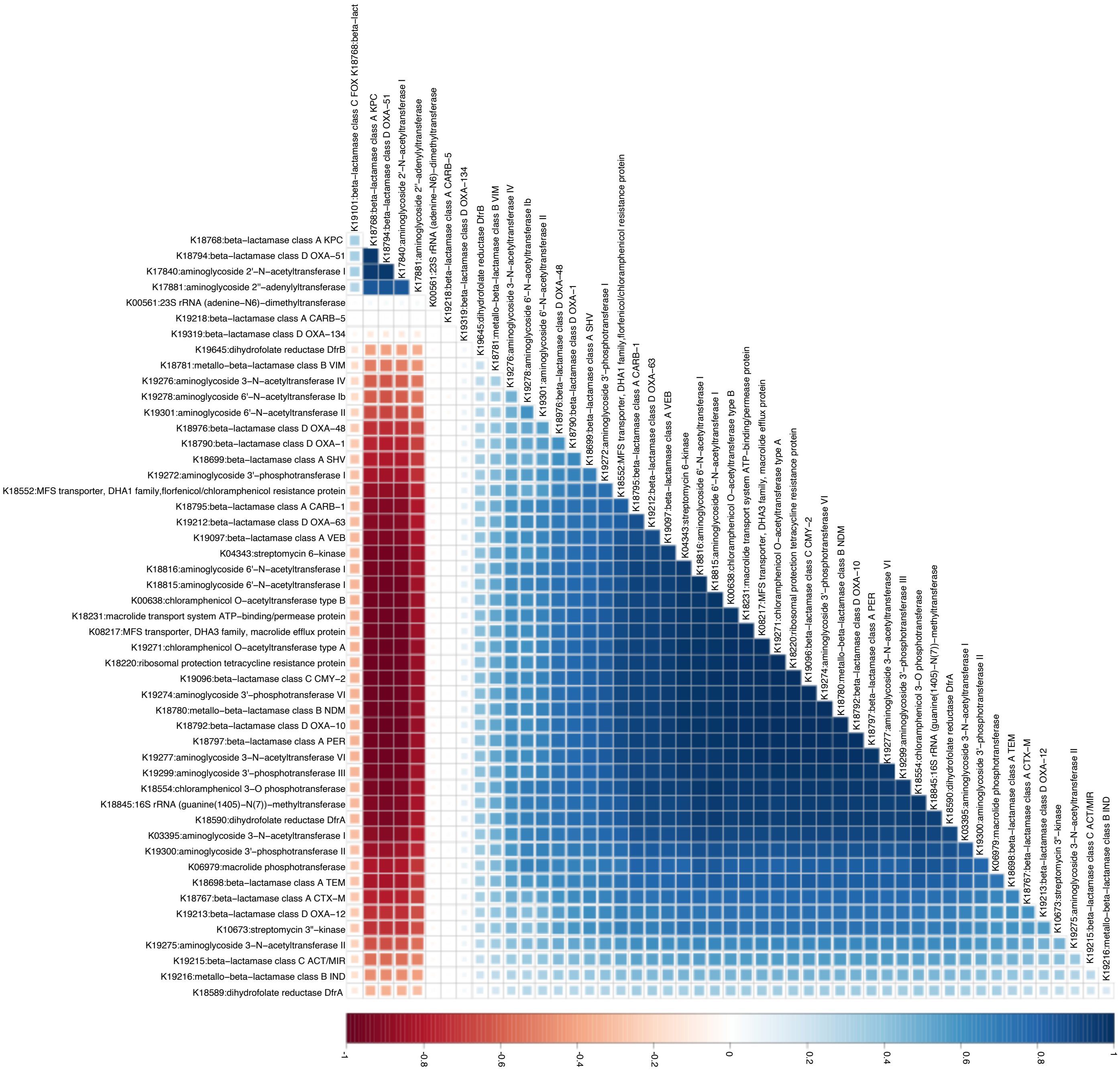

Supplementary Figure 15: Co-occurrence relationship between piARGs recovered from the residual covariance matrix ***Σ*** that are not explained by the observed covariates in the GLLVM model in Figure 5A with Diet:Starter and PenNo:P01 as references. Here, blue represent the positive correlation (taxa 1 increasing in abundance leads to taxa 2 increasing in abundance), and red represent the negative relationship (taxa 1 increasing in abundance leads to taxa 2 decreasing in abundance).

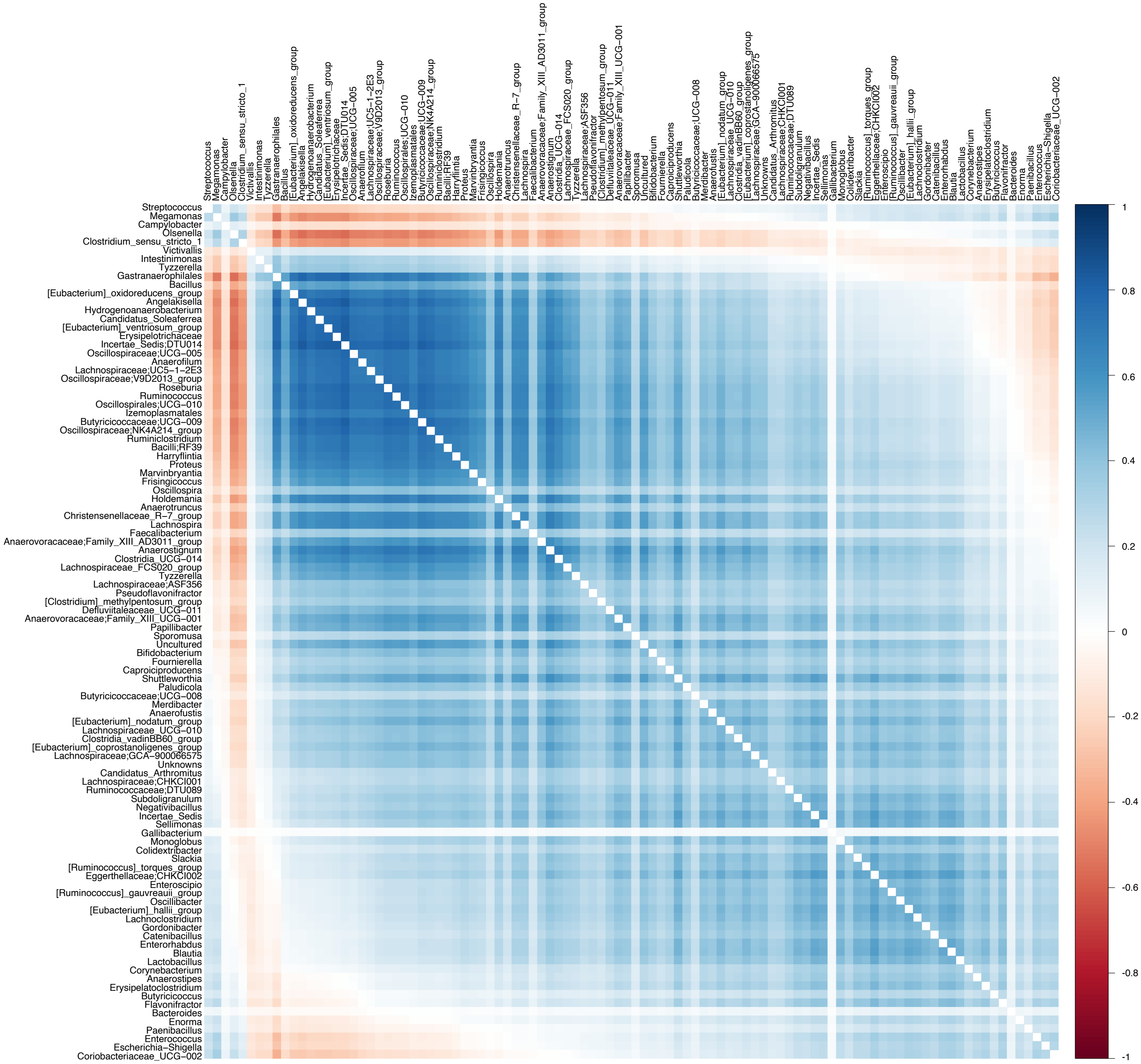

Supplementary Figure 16: Co-occurrence relationship between genera recovered from the residual covariance matrix $\boldsymbol{\Sigma}$ that are not explained by the observed covariates in the GLLVM model in Figure 4B with Diet:Grower and PenNo:P02 as references. Here, blue represent the positive correlation (taxa 1 increasing in abundance leads to taxa 2 increasing in abundance), and red represent the negative relationship (taxa 1 increasing in abundance leads to taxa 2 decreasing in abundance)

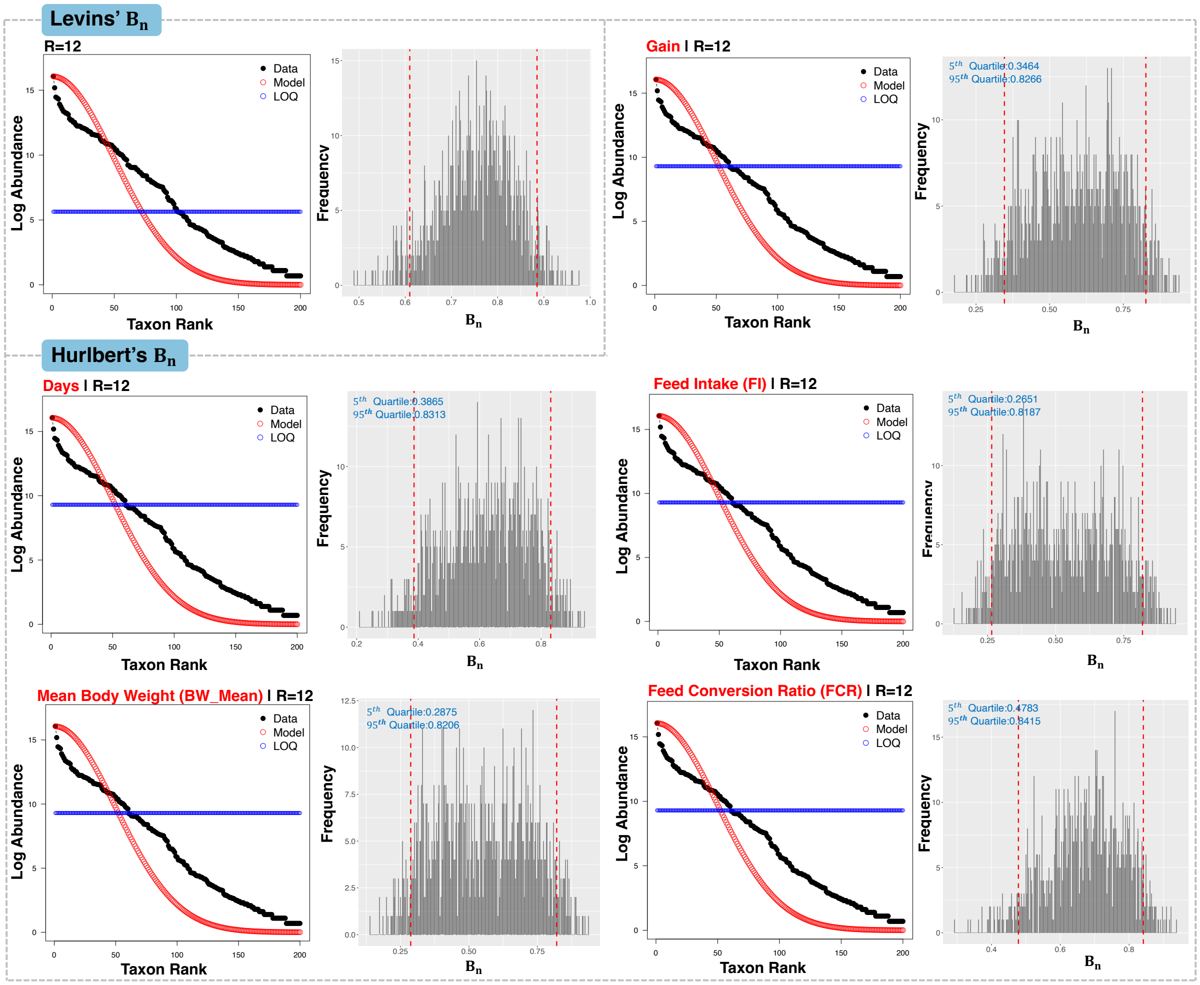

Supplementary Figure 17: Further statistics for results shown in Figure 7. For each analysis, the left figures represent the rank distribution of the taxa observed in the dataset represented as black. The lognormal rank distribution model is then shown as red circles. The limit of quantification threshold, are then shown as blue circles, is 1 standard deviations from zero. Any taxa that fall below the limit of quantification were excluded from the analyses. The right figure represents the null model distributions generated from applying Levin's B_N (top left) or Hurlbert's B_N (other set of figures) calculated from 999 randomly generated taxon distributions. Red dotted lines indicate the fifth and 95th quantiles. Taxa that are high when an environmental property (*Mean Body Weight*, *Feed Intake*, *Feed Conversion Ratio*, *Gain*, and *Days*) is low have a Hurlbert's B_N below the 5th quantile, and conversely, taxa that are high when the environmental property is high have a Hurlbert's B_N above the 95th quantile of those null models.

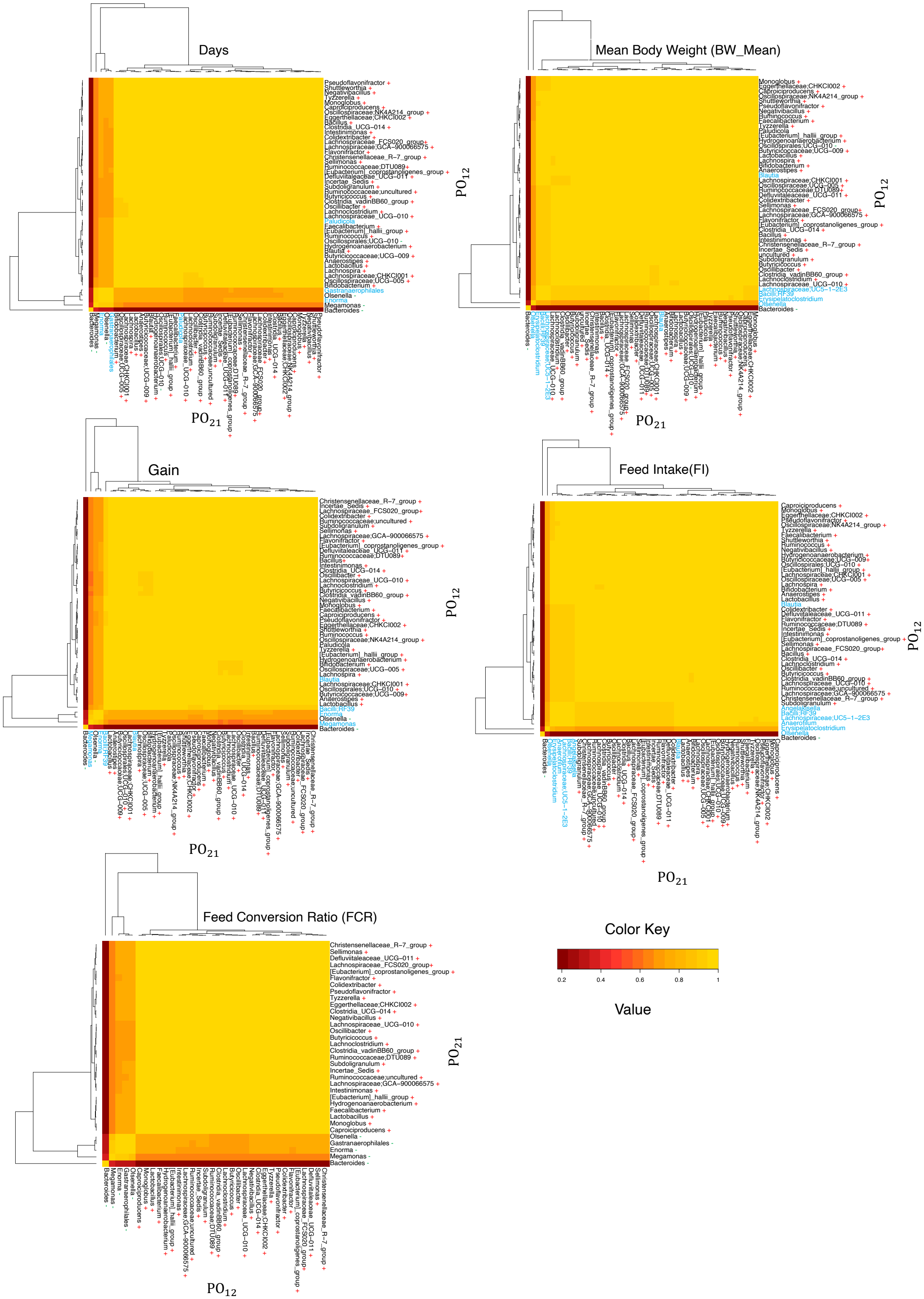

Supplementary Figure 18: The Proportional Overlap $PO_{i,j}$ which approaches 0 for genus pairs that are inversely related to each other, and approaches 1 for genus pairs that are positively related to each other. For each of the heatmap, we considered the genera identified through Hurlbert's B_N in Figure 7 for the considered environmental property (+ for positive,– for negative relationship, and light blue color where the association is “undecided”).

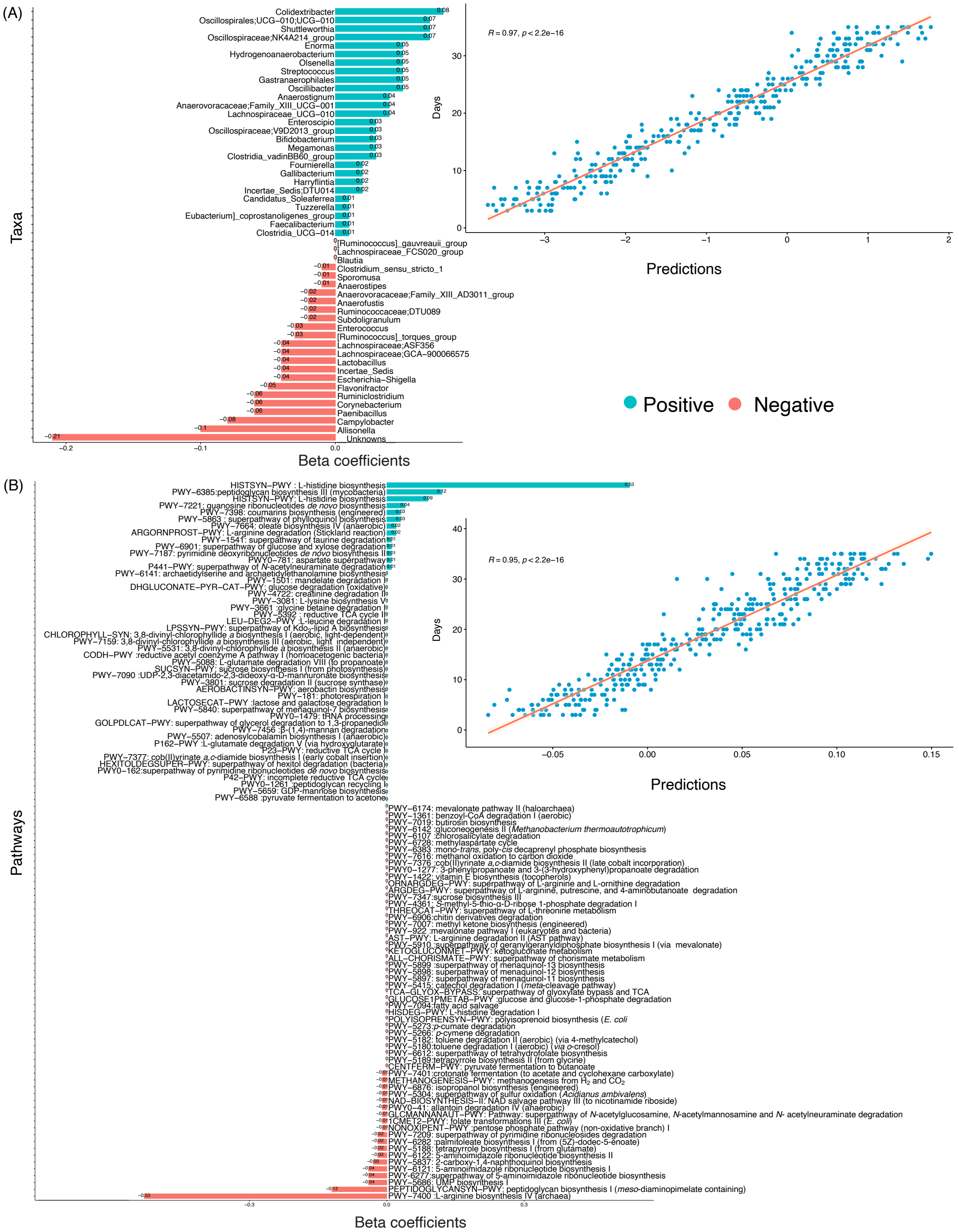

Supplementary Figure 19: Two disjoint sets of β −coefficients (those that are positively associated are shown in green, whereas those that are negatively associated are shown in red) returned from CODA-LASSO procedure using *Days* as a continuous outcome for (A) genera, and (B) MetaCyc pathways. The insets show the prediction accuracy with R value as a quality of fit criteria and shows good agreement.

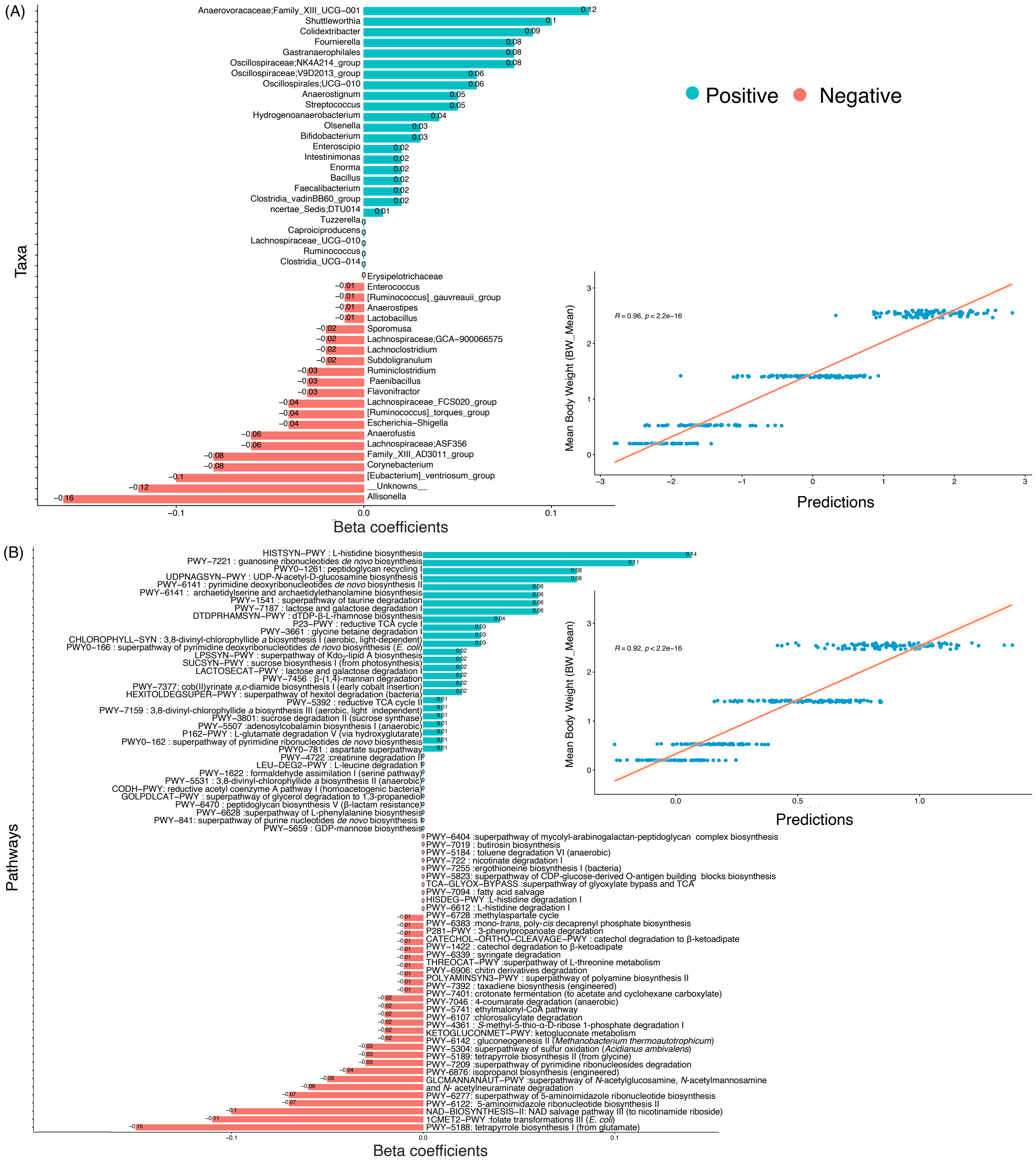

Supplementary Figure 20: Similar to Supplementary Figure 19, β −coefficients returned from CODA-LASSO procedure using *Mean Body Weight* (BW_Mean) as outcome.

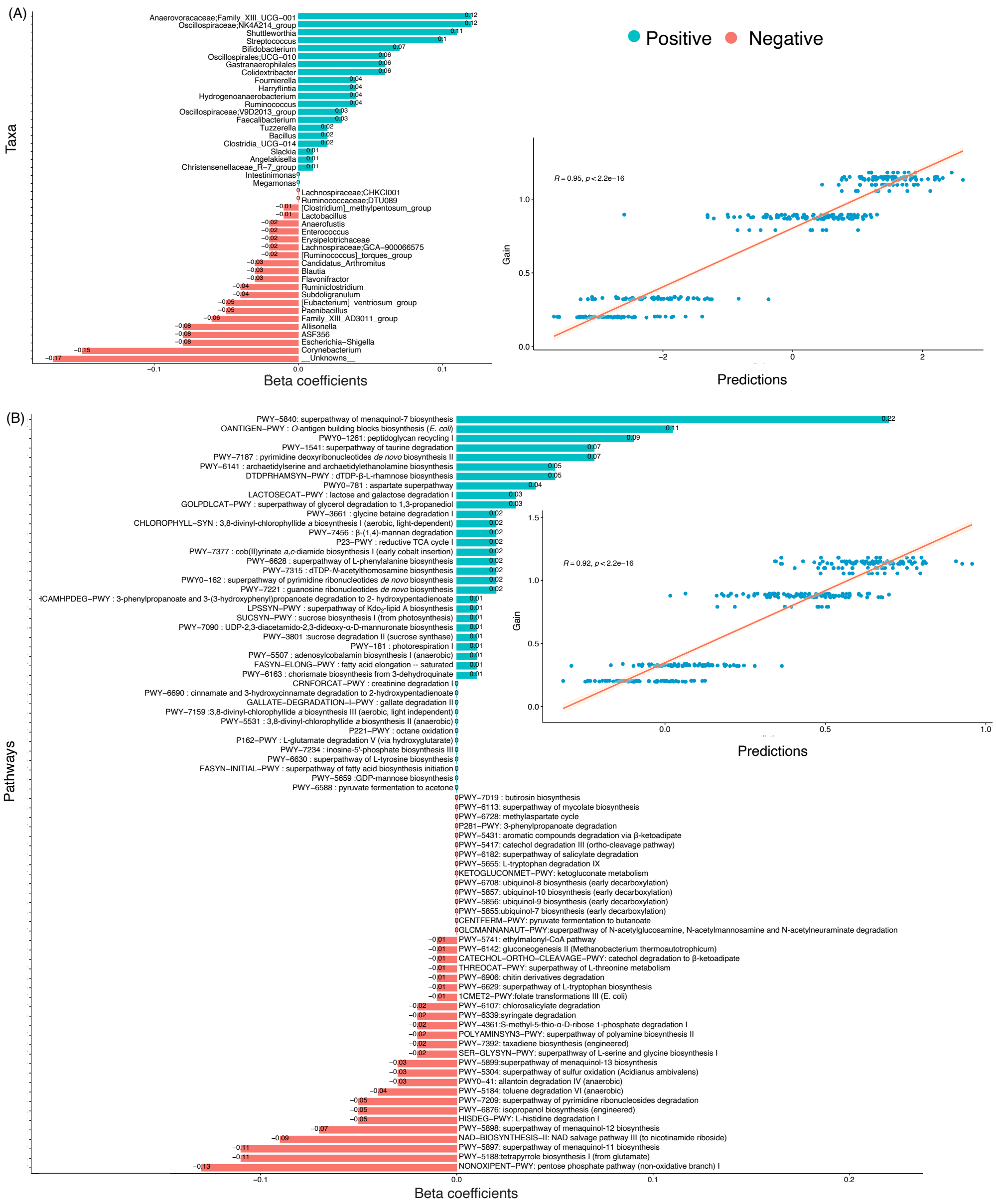

Supplementary Figure 21: Similar to Supplementary Figure 19, β −coefficients returned from CODA-LASSO procedure using *Gain* as outcome.

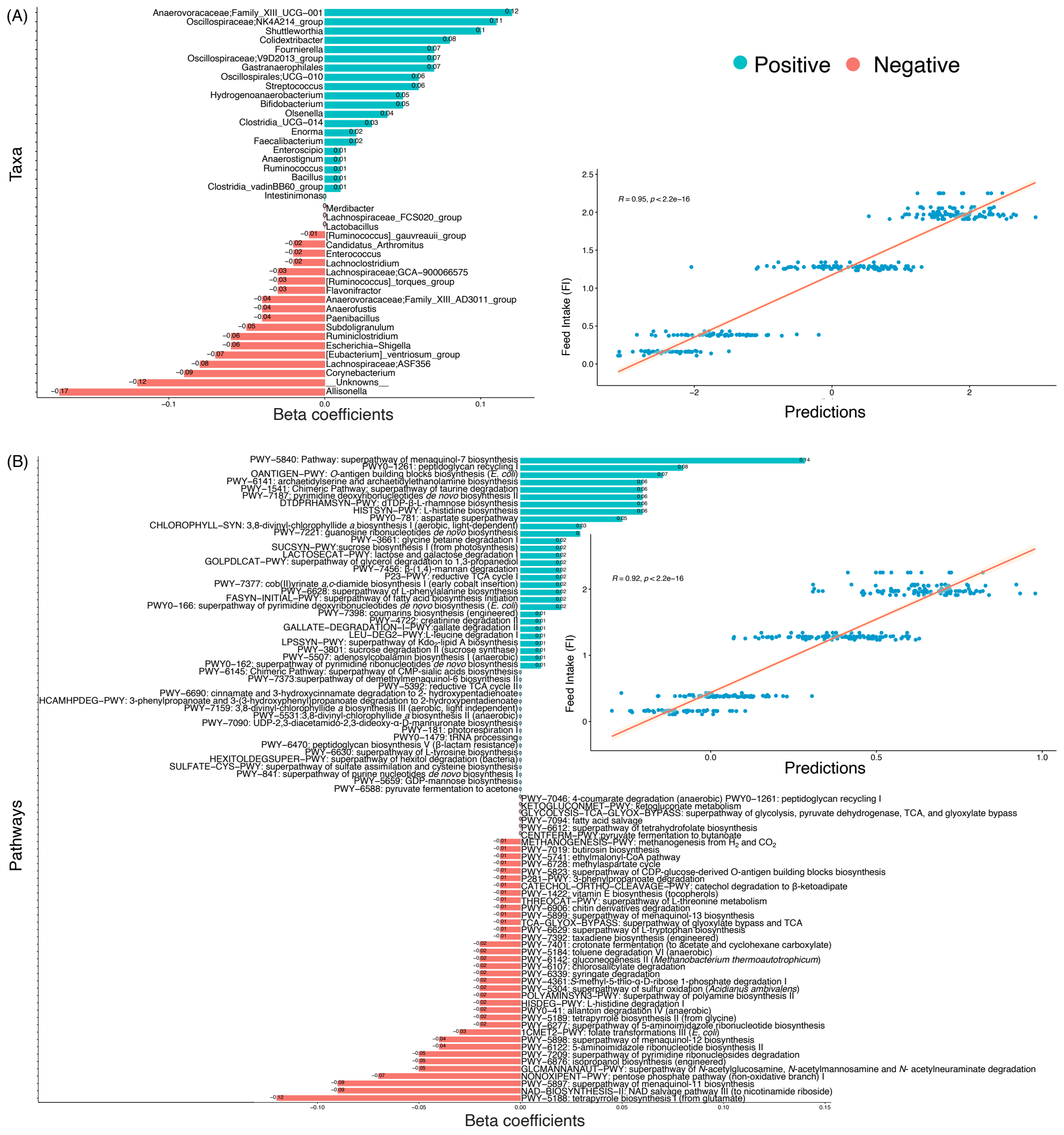

Supplementary Figure 22: Similar to Supplementary Figure 19, β −coefficients returned from CODA-LASSO procedure using *Feed Intake* (FI) as outcome.

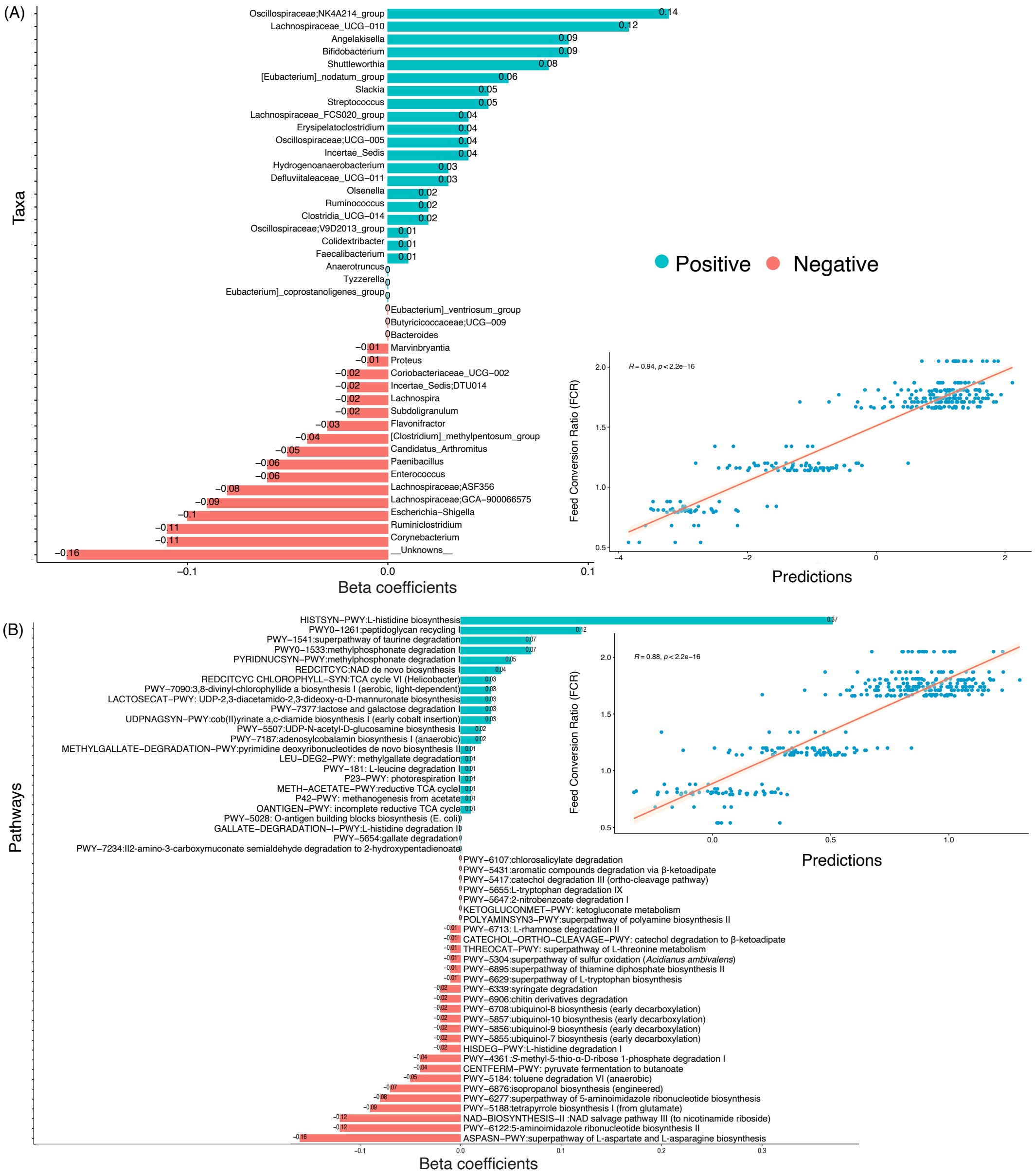

Supplementary Figure 23: Similar to Supplementary Figure 19, β −coefficients returned from CODA-LASSO procedure using *Feed Conversion Rate* (FCR) as outcome.

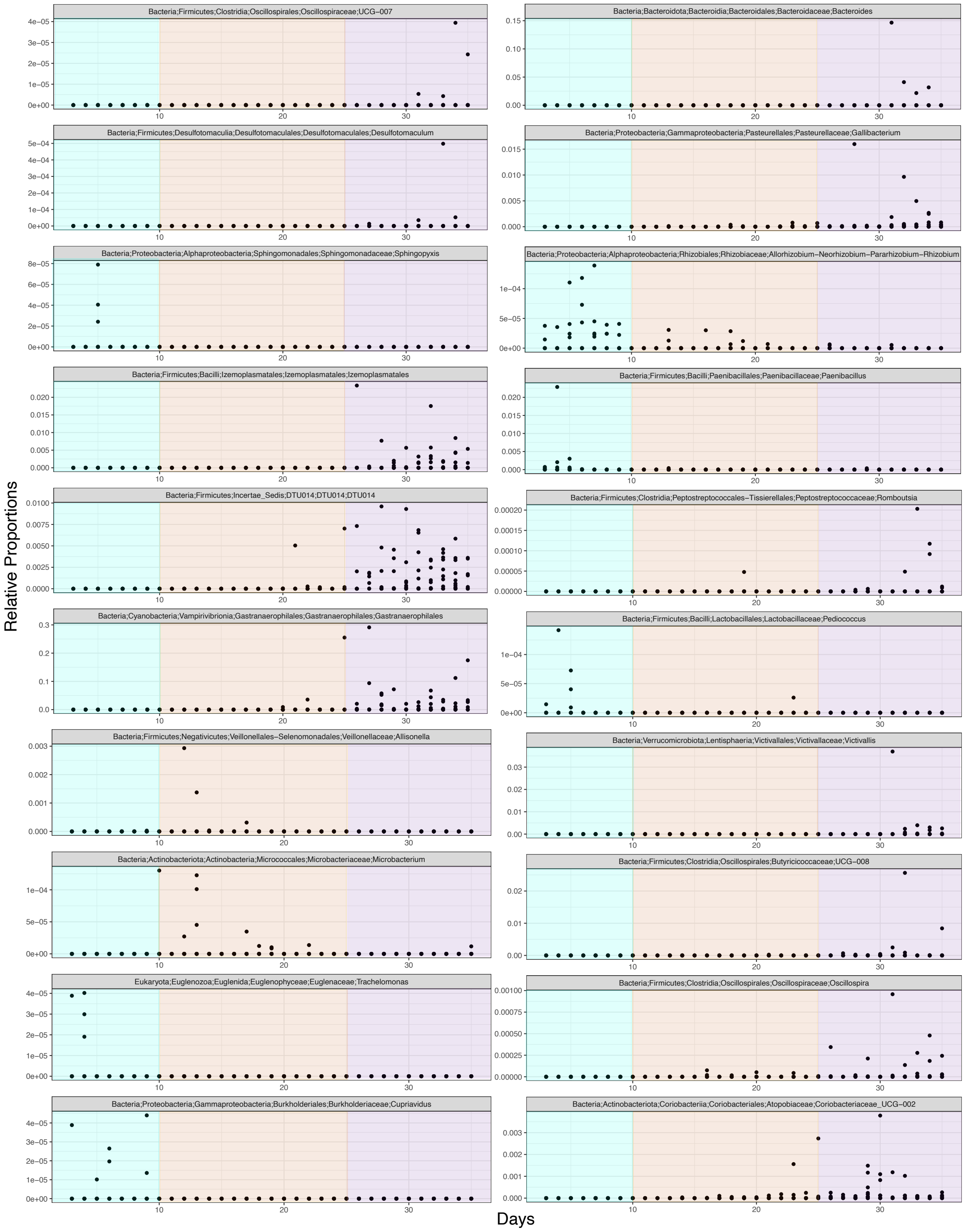

Supplementary Figure 24: For the genera identified as "Specific" in Figure 8A (darker) for *Days*, the temporal profile of the genera in lowest 25th quartile of *Spec* values are shown (remaining are shown in Supplementary Figure 25) with the backgrounds corresponding to the days when different diets were administered (i.e., *Starter*, *Grower*, and *Finisher*).

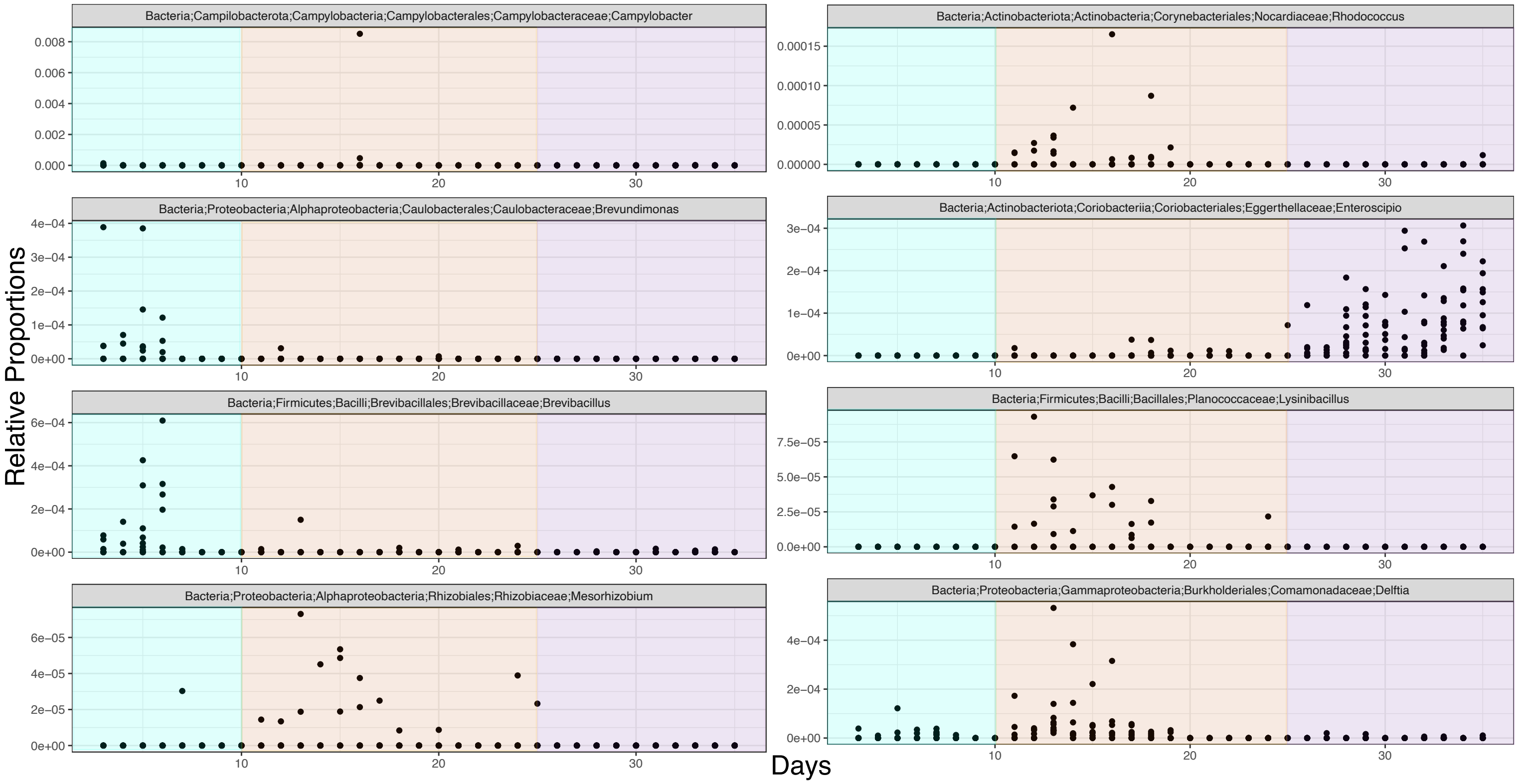

Supplementary Figure 25: Continuation of temporal profiles of genera specific to *Days* not shown in Supplementary Figure 24.

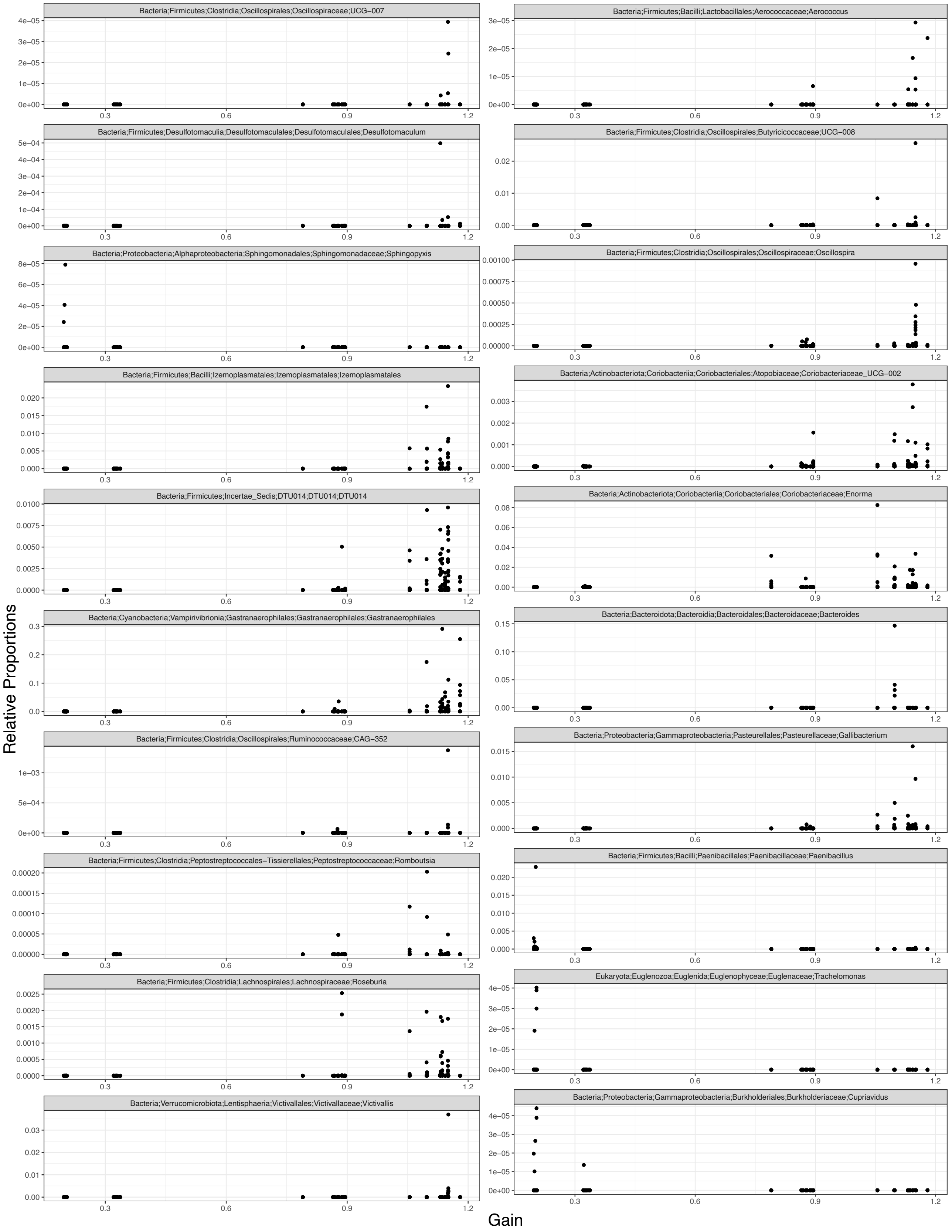

Supplementary Figure 26: For the genera identified as "Specific" in Figure 8A (darker) for *Gain*, the genera in lowest 25th quartile of *Spec* values are shown with their abundances in the whole range of *Gain* values (remaining are shown in Supplementary Figure 27).

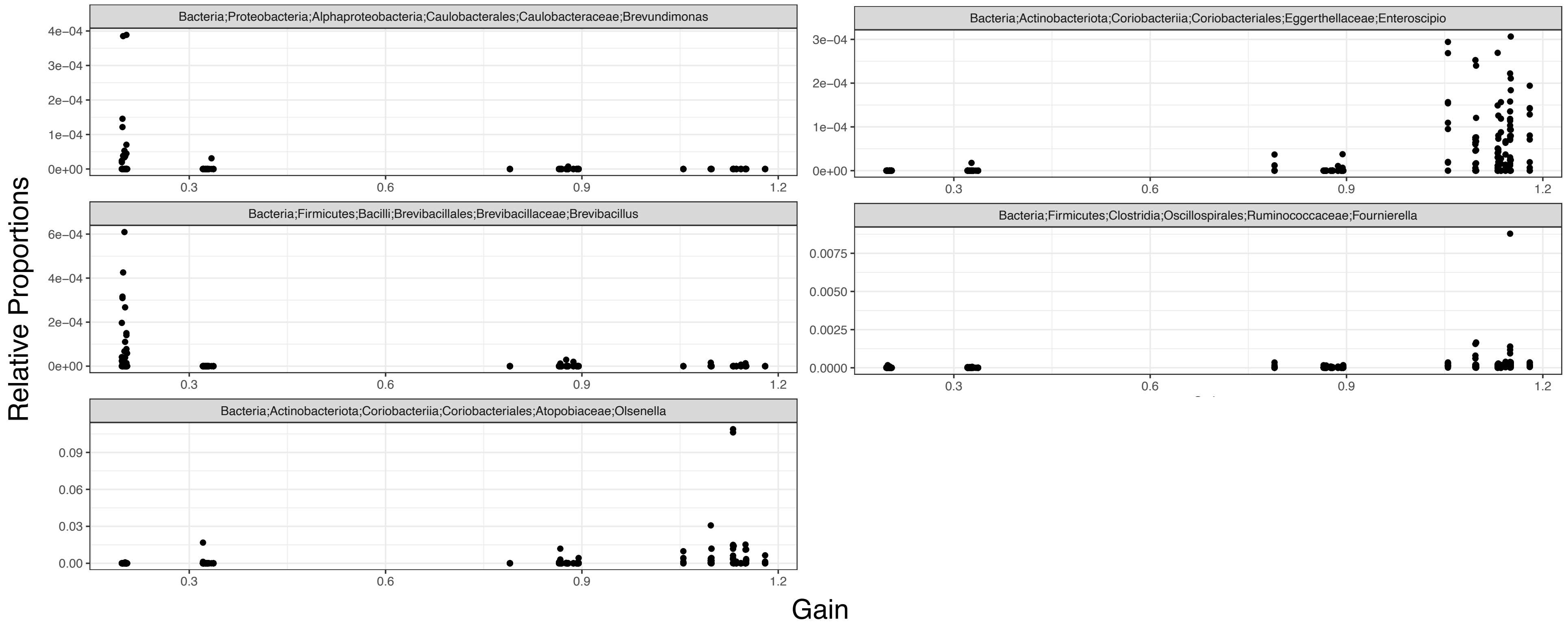

Supplementary Figure 27: Continuation of profiles of genera specific to *Gain* not shown in Supplementary Figure 26.

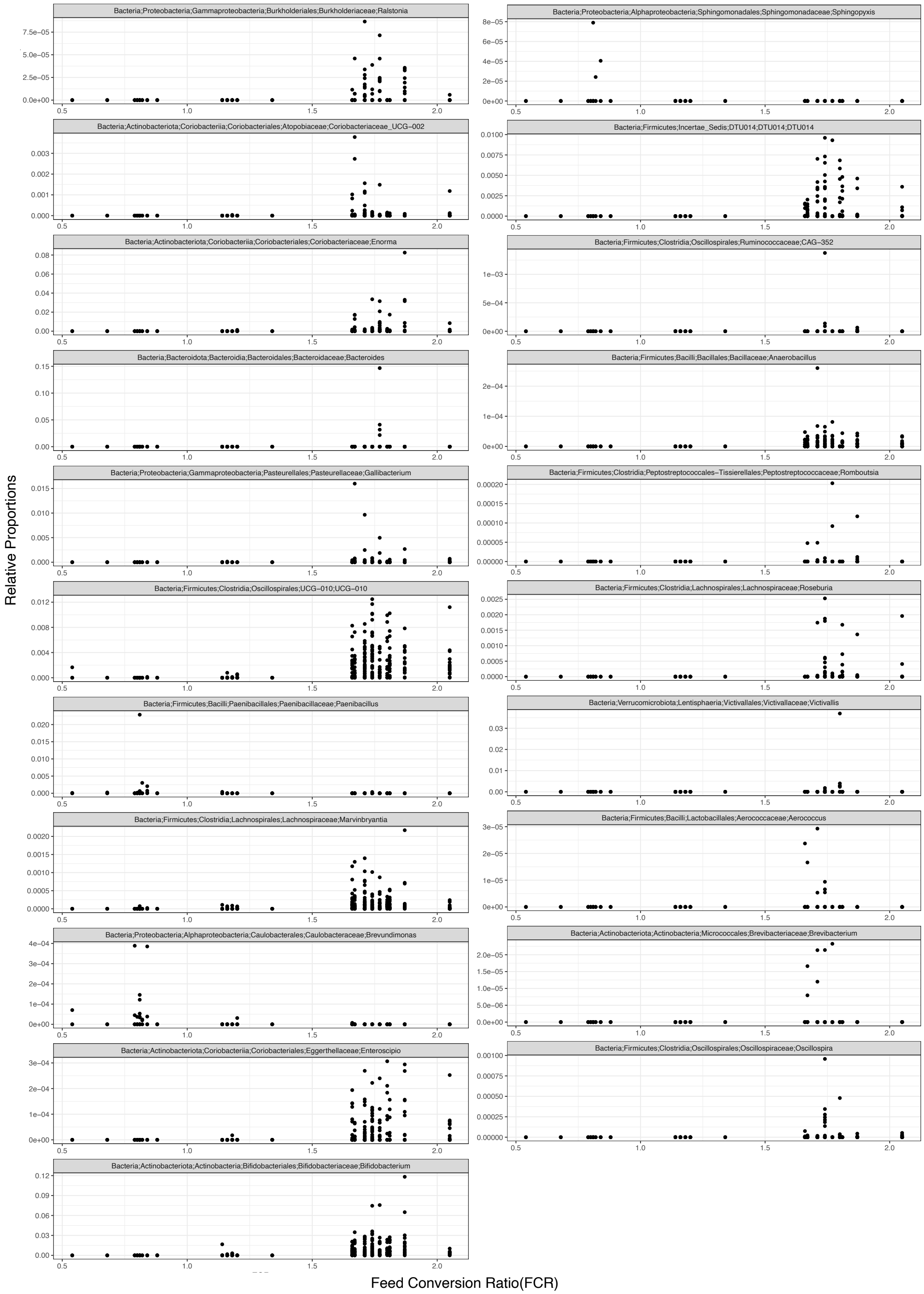

Supplementary Figure 28: For the genera identified as "Specific" in Figure 8A (darker) for *Feed Conversion Ratio* (FCR), the genera in lowest 25th quartile of *Spec* values are shown with their abundances in the whole range of *FCR* values.

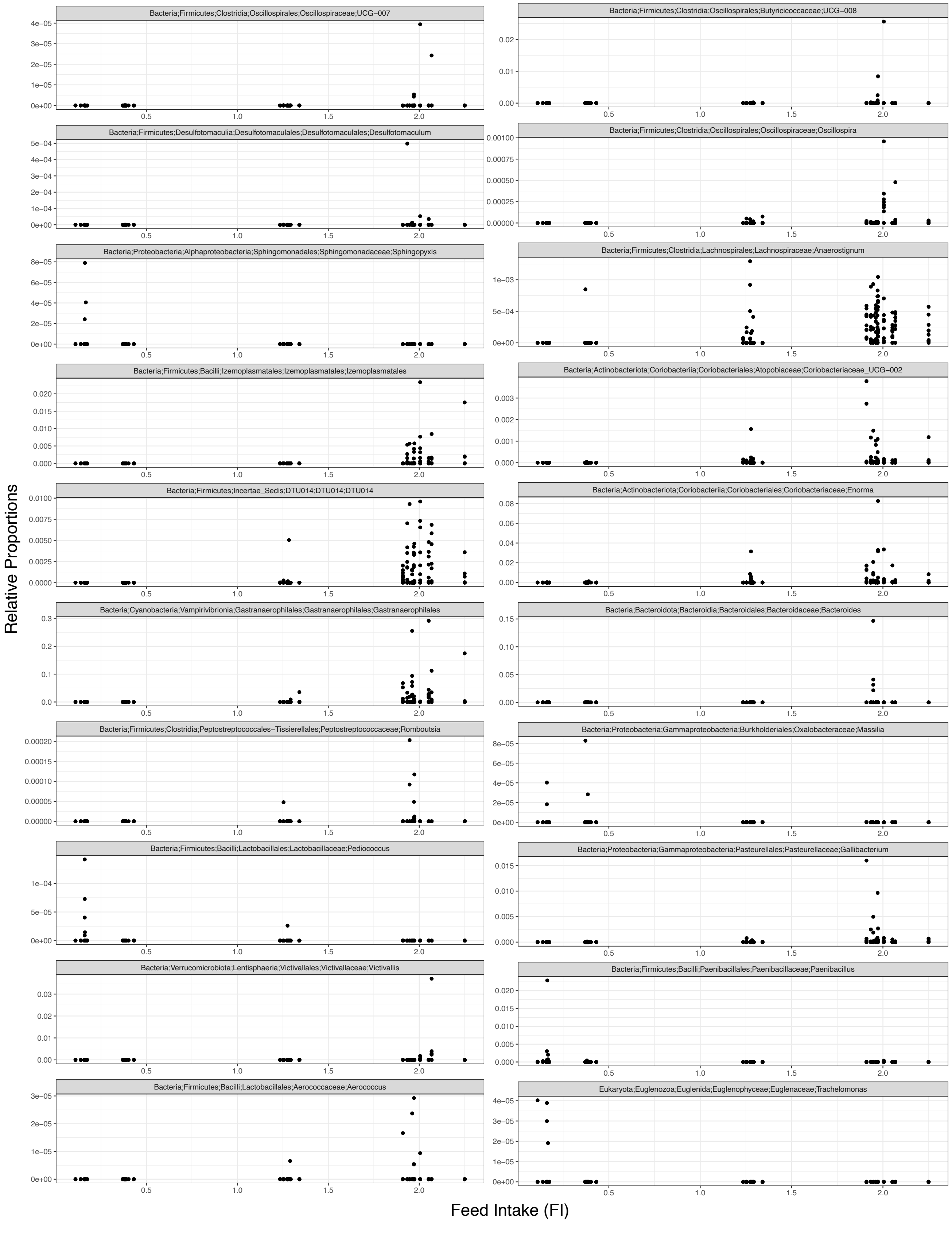

Supplementary Figure 29: For the genera identified as "Specific" in Figure 8A (darker) for *Feed Intake* (FI), the genera in lowest 25th quartile of *Spec* values are shown with their abundances in the whole range of *FI* values (remaining are shown in Supplementary Figure 30).

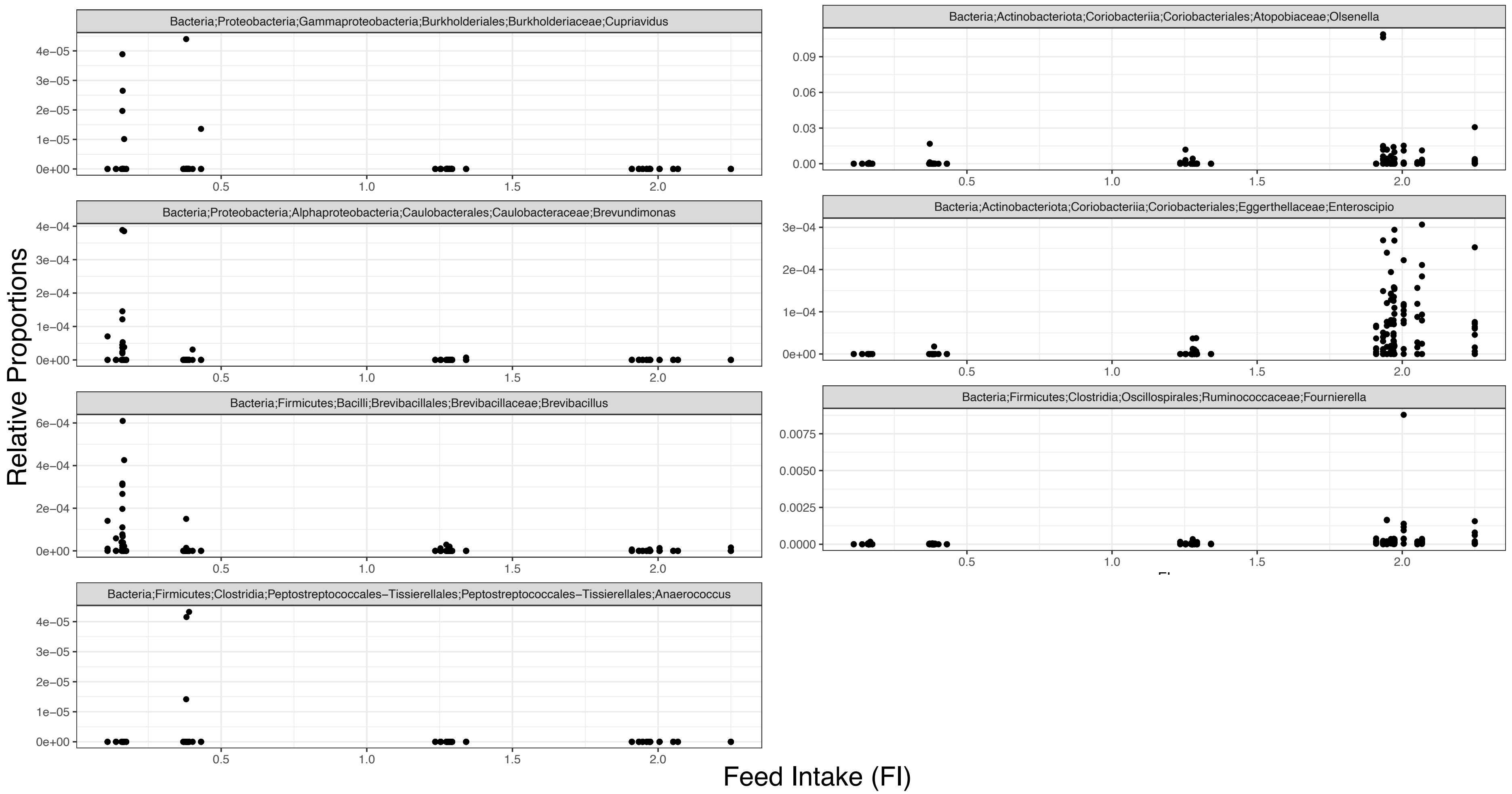

Supplementary Figure 30: Continuation of profiles of genera specific to *Feed Intake* (FI) not shown in Supplementary Figure 29.

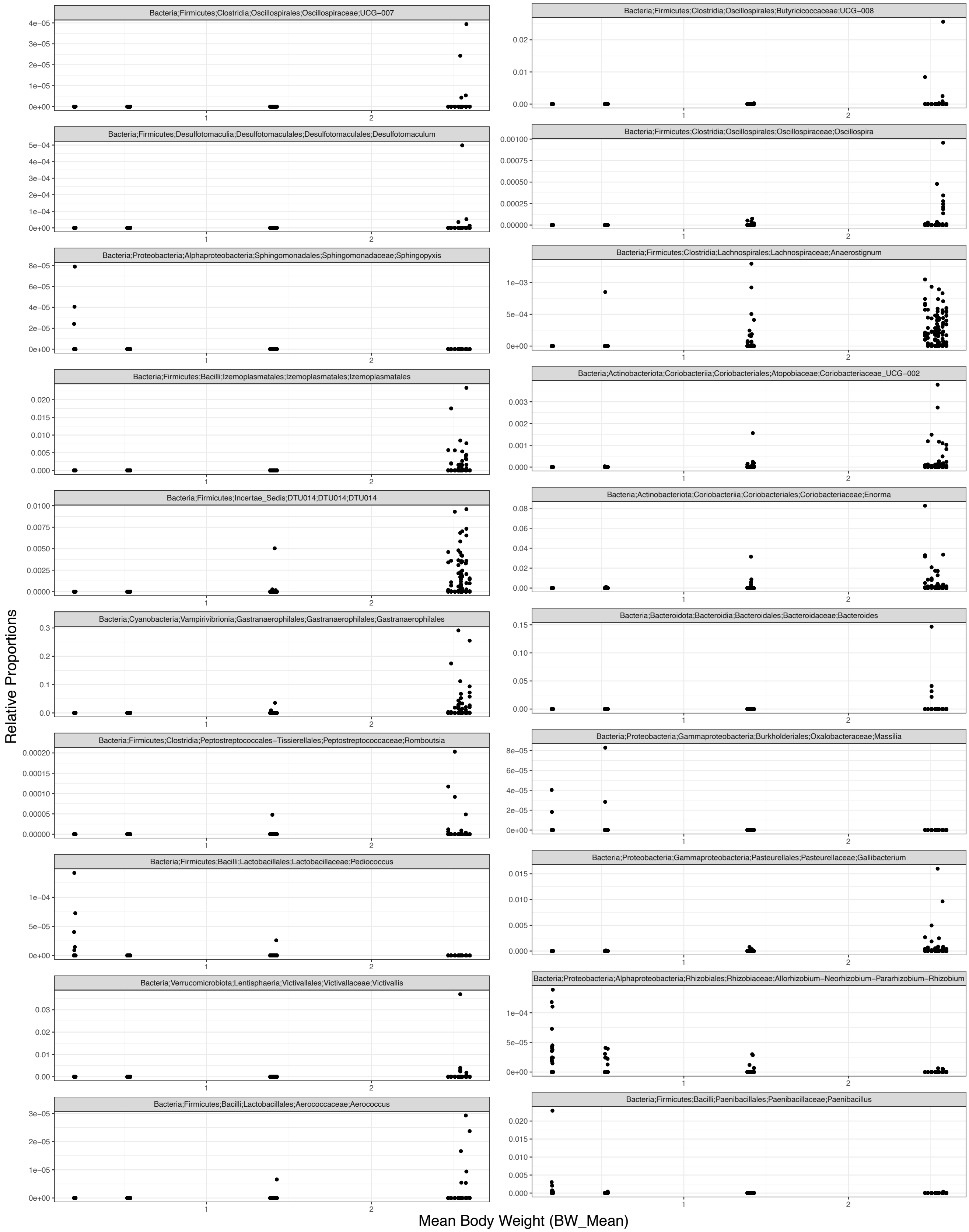

Supplementary Figure 31: For the genera identified as "Specific" in Figure 8A (darker) for *Mean Body Weight* (BW_Mean), the genera in lowest 25th quartile of *Spec* values are shown with their abundances in the whole range of *BW_Mean* values (remaining are shown in Supplementary Figure 32).

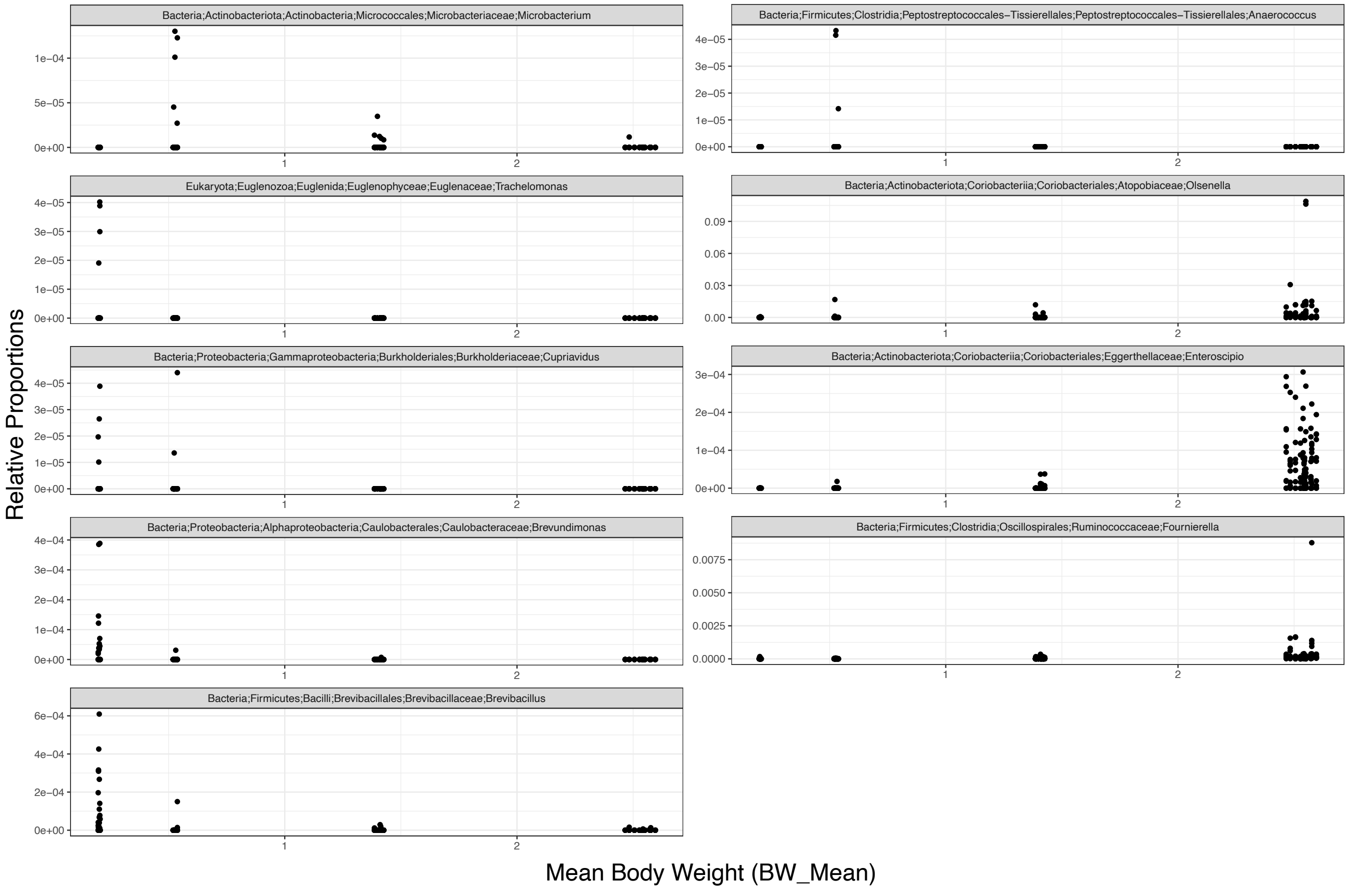

Supplementary Figure 32: Continuation of profiles of genera specific to *BW_Mean* not shown in Supplementary Figure 31.

Supplementary Table 1: Literature survey of significant genera implicated in GLLVM (Figures 2; Supplementary figures 12,14, and 16) and CODA-LASSO (Supplementary Figures 19-23) analyses.

No.	Significant Genera	Positively Associated Covariates	Negatively Associated Covariates	Implication in Literature	Reference
1.	Paenibacillus	BW_Mean, Diet_Grower Diet_Finisher, , P02, P03, P04, P05, P06, P07, P08, P09, P10	Day, FCR, Gain, Diet_Starter, P01, P12	Aerobic, gram-positive bacilli, associated with improved growth performance in chickens	(Ash et al., 1993; Ekim et al., 2020; H. Han et al., 2021)
2.	Victivallis	Gain, P01, P08, P09, P10, P11, P12	BW_Mean, FCR, FI, Diet_Grower, Diet_Finisher, P02, P03, P04, P05, P06, P07	Sugar fermenting anaerobe cocci, not well studied Found high in colorectal cancer	(Ferrario et al., 2017; Ocejo et al., 2019; Sánchez-Alcoholado et al., 2020; Zoetendal et al., 2003)
3.	Campylobacter	FCR, Gain, Diet_Finisher, P04, P05, P06, P10, P11, P12	BW_Mean, Day, P01, P02, P03, P07, P09	Gram negative rod, associated with decreased body weight, and feed efficiency in chickens	(Awad et al., 2015; Stahl & Vallance, 2019)
4.	Oscillospiraceae;V9D2013_group, Oscillospirales;UCG−010, NK4A214_group, UCG−005	BW_Mean, FCR, Day, FI, Gain, Diet_Starter, P01, P06	Diet_Finisher, Diet_Grower, P02, P03, P04, P05, P07, P10	Obligate anaerobe, early colonizer, found in 3-4 day old broiler and layer, colonize chicken gut after *Lachnospiraceae* in early hatch periods, elevated abundance noticed in laying hens fed on diet supplemented with coated sodium butyrate	(Miao et al., 2022; Ocejo et al., 2019; Segura-Wang et al., 2021; Zenner et al., 2021)
5.	Sporomusa	Diet_Grower, P02	BW_Mean, Day, FCR, P01, P03, P04, P05, P06, P07, P08, P09, P10, P11	Anaerobic, gram-negative bacteria, abundant in chicken cecal microbiome, not well understood, play role in fermentation processes	(Möller et al., 1984; Porcheron et al., 2012; X. Y. Zhu et al., 2002)
6.	Bacteroides	Gain, Diet_Finisher, P01, P12	BW_Mean, P02, P03, P04, P05, P08, P09, P10, P11	Gram negative, obligate anaerobe, plays an important role in breaking down complex molecules to simpler compounds	(Könönen et al., 2015; Y. Xiao et al., 2017)
7.	Izemoplasmatales	Day, FCR, FI, Gain, Diet_Finisher, P01	BW_Mean, P02, P04, P07, P09, P11, P12	An exceptional *Purine* degrader, encode multiple nucleases, found in gut of deep-sea invertebrates	(Durre & Andreesen, 1983; Hartwich et al., 2012; Wasmund et al., 2021; F. C. Zhu et al., 2020)
8.	Enorma	Day, Diet_Finisher, Diet_Grower, P02, P04, P10, P11, P12	FCR, Diet_Starter, P01, P08	Positively correlate with Haugh Unit in laying hens, produce organic acids and vitamins	(Gong et al., 2021; Khan & Chousalkar, 2020)
9.	Butyricicoccaceae;UCG−008	FI, P01	BW_Mean, Diet_Grower, P02, P03, P04, P06, P07, P09, P11, P12	Significant probiotic, lowered the abundance of *Campylobacter*, *Enterococcus* and *Escherichia/ Shigella* spp. in the chicken gut at day 40	(Eeckhaut et al., 2016)
10.	Roseburia	Day, FI, Gain, P01, P06, P11	BW_Mean, FCR, P03, P04, P07, P09	Butyrate producing bacteria, promote gut health	(Mesa et al., 2017)
11.	Gallibacterium	Day, Diet_Grower, P02	FCR, Diet_Starter, P01, P03, P05, P06, P08, P09	Gamma proteobacteria, an opportunistic pathogen, associated with wide range of avian host species	(El-Adawy et al., 2018)
12.	Holdemania	BW, FCR, P03	FI, Gain, P04, P05, P07, P08, P11, P12	Maintains gut health through lowering luminal pH and digestion of complex polysaccharides	(Khan & Chousalkar, 2020)
13.	Shuttleworthia	BW_Mean, Day, FCR, FI, Gain, P01	P02, P06, P10, P11	Butyrate producing probiotic, linked to improved broiler growth performance, decreased abundance noticed in broilers challenged with *Eimeria tenella*	(H. L. Chen et al., 2020; Y. Yang et al., 2019)
14.	Eubacterium_ventriosum_group, nodatum_group, hallii_group	Day, FCR	BW_Mean, FI, Gain, P06, P08, P09, P12	SCFA producing, gram positive cocci, found in chicken ceca	(Barnes, 1979; Harry et al., 1972; Wei et al., 2018; X. Y. Zhu et al., 2002)

15.	Enteroscipio	BW_Mean, FCR, Diet_Grower, P03	Gain, Diet_Starter, P08, P11	Gram positive, anaerobic rods, found in biogas reactor operated with food wastes and sewage sludge	(Danylec et al., 2018; Singh et al., 2021)
16.	Olsenella	Day, P05, P07, P08, P09, P10, P11, P12	--	Strictly anaerobic gram-positive non-motile bacilli, reported in the chicken microbiome	(Dewhirst et al., 2001; Ferrario et al., 2017)
17.	Oscillospira	FCR, Gain, Diet_Starter, P08, P10	Diet_Grower, P04, P12	Under studied anaerobe, positively correlate with the diversity, predictor of low FCR, considered as probiotic candidate	(Y. ran Chen et al., 2020; Kaplan et al., 2019; Y. Liu et al., 2021; Tkach et al., 2022; J. Yang et al., 2021)
18.	Anaerostignum	BW_Mean, Gain, Diet_Finisher, Diet_Grower, P08, P11, P12	Diet_Starter	Relative abundance reported higher in prediabetes group	(Jayanama et al., 2022)
19.	Streptococcus	BW_Mean, Day, Gain FI, Diet_Starter	FCR, Diet_Grower	Initial chicken gut colonizers, found high in low salmonella shedder chicks	(Kempf et al., 2020; Stanley et al., 2014)
20.	Bifidobacterium	BW_Mean, FCR, Gain, P07, P12	FI, P08	Single strain probiotic, improve broiler performance	(Krysiak et al., 2021)
21.	Megamonas	Day, P03, P09, P10, P11, P12	FI	Gram negative, non-spore forming pleomorphic bacteria, expressed enzymes for propionate production and efficiently colonizes in early hatch periods	(Polansky et al., 2016)
22.	Clostridium_sensu_stricto_1	Gain, Diet_Finisher, P11	BW_Mean, Day, P06, P12	Associated with necrotic enteritis	(Y. Yang et al., 2019)
23.	Angelakisella	FCR, Gain, Diet_Finisher, Diet_Grower, P07	Diet_Starter, P08	Associated with increased body weight, suppressed growth of SCFA bacteria	(Mailhe et al., 2017)
24.	Coriobacteriaceae_UCG−002	Gain, Diet_Finisher, Diet_Grower, P07	FCR, Diet_Starter, P06	Gram negative, known for lactate fermentation, negatively correlate with chicken weight Abundance increased with Angiogenin 4	(Farkas et al., 2022; Sultana et al., 2022)
25.	Frisingicoccus	FCR	Gain, P04, P05, P09, P12	Abundance declined in Eimeria infected group	(Memon et al., 2022)
26.	Hydrogenoanaerobacterium	Day, FI, Diet_Finisher, Diet_Grower	Diet_Starter, P12	Correlated with grower diet and bird's age	(Clavijo et al., 2022)
27.	Lachnospiraceae;GCA−900066575, ASF356	--	BW_Mean, FCR, FI, Gain, P03, P06	Anaerobes, correlate with high feed efficiency, predominantly found in 21 day old chickens, expressed glycolysis enzymes in chickens fed on wheat based diet	(Kempf et al., 2020; Y. Liu et al., 2021; Zou et al., 2022)
28.	Anaerovoracaceae;Family_XIII_UCG−001	BW_Mean, FI, Gain, Diet_Grower	Diet_Starter	Changed abundance was noticed in pups when pregnant mice were inoculated with LB	(Yu et al., 2020)
29.	Eubacterium_oxidoreducens_group	BW_Mean, FCR	FI, Gain, P12	Mesophilic anaerobe, showed different abundance profiles in *Salmonella* shedding categories	(Kempf et al., 2020)
30.	Ruminiclostridium	--	Day, FCR, FI, Gain, P08	Cellulose degrading bacteria, found as chicken gut core microbiome, negatively correlated with body weight, relative abundance increased in poultry birds due to heat stress	(Emami et al., 2022; Farkas et al., 2022; McKenna et al., n.d.)
31.	Fournierella	BW_Mean, FI, Gain, P10, P12	--	Identified in metagenome assembled genome of chicken GIT samples, correlated with CAZymes	(Segura-Wang et al., 2021)
32.	Colidextribacter	BW_Mean, Day, FI, Gain	--	Linked to improved intestinal health, positively correlated with inosine, guanosine and uridine levels in mice.	(Y. Liu et al., 2022; X. Zhang et al., 2022a)
33.	Allisonella	--	BW_Mean, Day, FI, Gain	Histamine producing novel firmicute, found abundant in in 6-week-old broiler chickens, negatively correlated with *Bifidobacterium*	(Garner et al., 2002; Lopera et al., 2021; Oakley & Kogut, 2016)
34.	Lachnospiraceae;UC5−1−2E3, FCS020_group	FCR	BW_Mean , FI, Diet_Finisher	Linked to improved feed efficiency by producing SCFAs, found abundant in good feed efficiency chicken group	(Broom, 2018; Z. Li et al., 2018a; Y. Liu et al., 2021; Stanley et al., 2016)
35.	Gastranaerophilales	BW_Mean, Day, FCR, P03	--	Positively correlated with Bacteroides	(S. S. Xiao et al., 2021)
36.	Blautia	--	Gain, P05, P06, P11	Expressed enzymes for propionate production pathways, *B hominis* and *B F42* found in low and high residual feed intake broiler group	(J. Liu et al., 2021; Polansky et al., 2016)

37.	Lachnoclostridium	Diet_Starter	Diet_Finisher, Diet_Grower	Among the top most abundant genera, found abundant in high welfare diet group (chicken), linked with feed protein content	(McKenna et al., 2020; Wu et al., 2022; Zou et al., 2018)
38.	Arthromitus	Diet_Starter	Diet_Grower, P10	Segmented filamentous, predominantly found in chicken GIT, at day 10 and replaced by *Lactobacillius* at day 35, reduced abundance observed in broiler ceca challenged with *Eimeria*	(Antonissen et al., 2016; L. Wang et al., 2016)
39.	Erysipelotrichaceae	BW_Mean, P06	FI	Found abundant in chickens fed on corn diet at day 24 and 40	(Zou et al., 2022)
40.	Erysipelatoclostridium	FCR	Day, P10	Quite unknown genus, found as differentially abundant group in 0 day post hatch chicks	(Richards-Rios et al., 2020)
41.	Lachnospira	P01	Gain, P02	Anaerobic, increased with age, maintains gut microbial balance	(S. S. Xiao et al., 2021)
42.	Lachnospiraceae_UCG−010	FCR	Gain, P11	SCFAs producer, positively correlated with broiler growth performance, decreased relative abundance reported in broilers exposed to ammonia	(Biddle et al., 2013; Carrasco et al., 2019; H. Han et al., 2021)
43.	Anaerotruncus	Diet_Starter	Diet_Grower	Positively correlated with broiler diet containing fructoligosaccharides	(Kumar et al., 2019)
44.	Intestinimonas	Diet_Starter	Diet_Grower	Butyrate producing, one of the abundant chicken gut core microbiome representatives, increased relative abundance noticed in chickens fed on soy protein diet, positive effects on chicken body weight in initial days	(Bui et al., 2015; Clavijo et al., 2022; Kläring et al., 2013; Q. Zhang et al., 2022)
45.	Butyricicoccus	Diet_Starter	Diet_Grower	Found abundant in high feed efficiency chickens	(J. Liu et al., 2021)
46.	Enterococcus	--	Day, FCR	Found high in low salmonella shedder chicks, negatively correlated with poultry growth	(Kempf et al., 2020; Z. Li et al., 2018a)
47.	Anaerofustis	--	BW_Mean, FI	Gram positive, anaerobic, SCFA producing rods, positively correlated with chicken's abdominal fat	(*Anaerofustis*, n.d.; H. L. Chen et al., 2020)
48.	Butyricicoccaceae;UCG−009	BW_Mean	FI	Found abundant in high feed efficiency chickens	(J. Liu et al., 2021)
49.	Anaerovoracaceae;Family_XIII_AD3011_group	--	BW_Mean, Gain	Found abundant in dysbiotic gut of diabetic individuals with damaged retina	(Huang et al., 2021)
50.	Faecalibacterium	P05	Gain	Dominant in cecal microbiota in high residual feed intake group	(J. Liu et al., 2021)
51.	Subdoligranulum	--	FI, Gain	SCFA producing probiotic candidate, identified as core chicken gut microbiome	(Ijaz et al., 2018; Lund et al., 2010; Van Hul et al., 2020)
52.	Paludicola	P01	P10	Novel genus of unknown function, increased abundance noticed in ducks raise on corn based diet containing ferulic acid	(Y. Li et al., 2017; Y. Liu et al., 2021)
53.	Catenibacillus	--	Gain	Abundance reduced in chickens fed on diet supplemented with fulvic acid	(Feng et al., 2022)
54.	Tyzzerella	P03	--	Relative abundance increased in poultry birds due to heat stress	(Emami et al., 2022)
55.	Clostridia_UCG−014, vadinBB60_group	--	P10	Highly polyphyletic, found high in poor performing birds	(Stanley et al., 2016)
56.	Oscillibacter	Day	--	SCFA producer, found as common genera in litter, carcass and fecal samples, found abundant in high efficiency broiler chicken group	(Y. Liu et al., 2021; Oakley et al., 2014)
57.	Proteus	P11	--	Opportunistic pathogen, cause cellulitis in chickens	(Sanches et al., 2020)
58.	Defluviitaleaceae_UCG−011	P08	--	Negatively correlated with body weight in broiler chickens, found abundant in cows fed on wheat based diet	(Farkas et al., 2022; Savin et al., 2022)
59.	Lactobacillus	--	Day	Probiotics, linked to improved poultry growth and performance by producing acids and lowering gut pH	(Chateau et al., 1993; Z. Li et al., 2018b)
60.	Harryflintia	Gain	--	Under studied, gram negative rods, isolated from chicken ceca	(Petzoldt et al., 2016)
61.	Slackia	FCR	--	Found abundant in low weight chickens, negatively correlated with fat metabolism	(X. Zhang et al., 2022b)

62.	[Clostridium]_methylpentosum_group	--	FCR	Mesophilic anaerobe, involve in fermentation of pentoses and methylepentoses	(Reimer et al., 2022)
63.	Clostridiaceae;Candidatus_Arthromitus	--	FCR	Segmented, non-pathogenic filamentous bacteria, high abundance reported in 7 to 14 day old chickens,	(Richards-Rios et al., 2020; Snel et al., 1995)

Supplementary Table 2: Literature survey of significant piARGs implicated in GLLVM (Figures 3; Supplementary figures 13, and 15) analyses. The details of the threat levels of the piARGs are shown beneath the table.

No.	Drug Group	Predictive Antimicrobial Resistance Gene	Threat Level	Positively Associated Covariates	Negatively Associated Covariates	Significance	References
1	Aminoglycoside (DG01447)	K17840:aminoglycoside 2'−N−acetyltransferase I	B6 B12	BW_Mean, FI, Gain, P04, P05, P06, P07	Day, Diet_Finisher, Diet_Grower, P02, P03, P08, P09, P10, P11, P12	Neomycin, apramycin showed positive effect on growth<br><br>Antimicrobial growth promoters (15 different classes of antimicrobials) have no impact on the gut bacterial community structure (alpha or beta diversity) so effect on performance is not significant.	(Allende et al., 2021)<br><br>(Paul et al., 2022)
		K19274:aminoglycoside 3'−phosphotransferase VI	B1	Day, P04, P11	FCR, FI, P03,P05, P06, P07, P09, P12		
		K17881:aminoglycoside 2''−adenylyltransferase	B1	Diet_Finisher, FCR, P03, P05, P08, P10, P11	BW_Mean, Gain		
		K19272:aminoglycoside 3'−phosphotransferase	B1, B7	Diet_Starter, Diet_Finisher, Gain, P02, P11	BW_Mean		
		K19277:aminoglycoside 3−N−acetyltransferase VI	B7	Day, Diet_Finisher	FCR		
		K19300:aminoglycoside 3'−phosphotransferase II	B6	P06, P10, P12	--		
		K19301:aminoglycoside 6'−N−acetyltransferase II	B1, B6	P02 , P03	P08		
		K19276:aminoglycoside 3−N−acetyltransferase IV	B6	Diet_Grower, P11	Diet_Starter		
		K19275:aminoglycoside 3−N−acetyltransferase II	B1, B7	Gain, P06	--		
		K19278:aminoglycoside 6'−N−acetyltransferase Ib	B1, B6	P03	--		
		K18815:aminoglycoside 6'−N−acetyltransferase I	B1, B6, B7	P01	--		
		K19299:aminoglycoside 3'−phosphotransferase III	B11	BW_Mean	--		
2	Carbapenem (DG01458)	K18794:beta−lactamase class D OXA−51	B1	Diet_Starter, BW_Mean, FCR, P05	Day, Diet Finisher, Diet Grower, P02, P04, P06, P08, P10, P12	Promoted weight gain upto 9.5 % in broiler<br><br>Novel carbapenemase (SYN-006), formulated for oral administration in porcine model of antibiotic-mediated gut dysbiosis. It was recorded that it protects the gut microbiome from all classes of beta-lactam antibiotics and reduce emergence of carbapenem-resistant pathogens.	(Salaheen et al., 2017)<br><br>(Connelly et al., 2019)
		K18780:metallo−beta−lactamase class B NDM	A2, B1	P04, P07	Day, FI, P03, P05, P06, P09, P11, P12, P12		
		K19319:beta−lactamase class D OXA−134	B1	BW_Mean, Diet_Grower, FCR, P05, P12	Diet_Starter, FI, P04, P10		
		K18768:beta−lactamase class A KPC	A2, B1, B6	P05 , P08	Diet_Grower		
		K18976:beta−lactamase class D OXA−48	A2	P09	Diet_Finisher		
		K19216:metallo−beta−lactamase class B IND	U	P07 , P09	--		
3	Extended-spectrum cephalosporin (DG01776, DG01777),<br><br>Extended spectrum penicillin (DG01780)	K18790:beta−lactamase class D OXA−1	B4, B6, B7, B9	Diet_Starter, FI, Gain, P02, P03, P08, P09, P10, P12	BW_Mean, Day, Diet_Finisher, P01	In an experiment on mice model during gestation period mother mice administered with penicillin for specific days before she given birth and also all along the weaning, it was recorded that a significant increase in fat mass in new born and over all mass that	(Vallianou et al., 2021)

						persisted during adulthood.	
**4**	Second-generation cephalosporin (DG01775)<br><br>Third-generation cephalosporin (DG01776)	K19096:beta−lactamase class C CMY−2	B4, B7	P04, P07, P10	Day, FCR, FI, P02, P03, P04, P05, P06 , P07, P09, P10, P11	In a study it is clearly recorded that cephalosporins, macrolides, quinolones, and sulfonamides were not associated with BMI change over time.	(Furlong et al., 2019)
		K19097:beta−lactamase class A VEB	B1 B4 B6	BW_Mean, P02, P09, P10, P12	Diet_Finisher, P04, P05		
		K18792:beta−lactamase class D OXA−10	B1 B4 B6	Diet_Starter, Day, P04	FCR, FI, Gain, P05, P06, P07		
**5**	Extended-spectrum cephalosporin (DG01776, DG01777),<br><br>Monobactam (DG01454)	K18797:beta−lactamase class A PER	B1 B4 B6 B7 B8	P07	Diet_Grower, FCR, P12	Monobactams are antibiotics which are beta lactams and are active against gram negative bacteria than gram positive bacteria. No effect on performance parameters.	(Wetzel et al., 2010)
		K18797:beta−lactamase class A PER	B1 B4 B6 B7 B8	--	Day, Diet_Finisher		
		K18699:beta−lactamase class A SHV	B1 B4 B5	--	P01		
		K18767:beta−lactamase class A CTX−M	B1 B4 B7 B8 B9	--	P01		
**6**	Extended-spectrum cephalosporin (DG01776, DG01777),<br><br>Carbapenem (DG01458)	K18781:metallo−beta−lactamase class B VIM	B11	P09	--	Has significant role in weight gain.	(Del Fiol et al., 2018)
**7**	Narrow-spectrum penicillin (DG01779)	K19212:beta−lactamase class D OXA−63	U	FCR, FI	Gain, P02, P04, P07, P09, P11	Different antibiotics use in poultry including penicillin has significant effect on weight gain and egg laying.	(Costa et al., 2017)
		K19213:beta−lactamase class D OXA−12	U	FCR, FI, P01	P02, P04, P07, P09, P11		
**8**	Carbenicillin (DG00519)	K18795:beta−lactamase class A CARB−1	B6, B7	Diet_Grower, FCR, FI, P01	Diet_Starter, Gain, P02, P03, P04, P07, P11	Marked affects as growth promoter.	(Feighner & Dashkevicz, 1987),<br><br>(Chattopadhyay, 2014)
		K19218:beta−lactamase class A CARB−5	B1	Diet_Finisher, Diet_Grower, Gain, P02	BW_Mean, P01, P10		
**9**	Trimethoprim (DG01581)	K19645:dihydrofolate reductase DfrB	B6, B7	FI, P03, P06	Diet_Starter, BW_Mean, Gain, P07	Showed negative effects on water intake, feed intake and also on body weight gain	(Piercy et al., 1984)
		K18589:dihydrofolate reductase DfrA	B1, B6, B7	P08, P11	--		
**10**	Extended-spectrum penicillin (DG01780),<br><br>Second-generation cephalosporin (DG01775)	K19215:beta−lactamase class C ACT/MIR	U	--	P08	The third generation cephalosporin, polypeptide and streptogramin improve food conversion and body-weight gain by preventing and controlling the respiratory and Gastrointestinal infections.	(Diarra & Malouin, 2014)<br><br>(Oelschlaeger, 2021)
**11**	Phenicol (DG01576)	K18552:MFS transporter, DHA1 family,florfenicol/chloramphenicol resistance protein	B6 B7	--	P08	Have positive effect on the growth parameters	(Shalaby et al., 2006)
**12**	Cephalosporins (No DG number)	K19101:beta−lactamase class C FOX	U	P08	--	Ceftiofur (a third generation	(Diarra & Malouin, 2014)

						cephalosporin), bacitracin (polypeptide) and virginiamycin (streptogramin) commonly used antibiotics in poultry production to prevent and control infections (respiratory diseases and necrotic enteritis) and to improve feed conversion and body-weight gain.	(Chika et al., 2018)

Threat level by CDC

A. Urgent threats
A1. Clostridium difficile
A2. Carbapenem-resistant Enterobacteriaceae (CRE)
A3. Drug-resistant Neisseria gonorrhoeae

B. Serious threats
B1. Multidrug-resistant Acinetobacter
B2. Drug-resistant Campylobacter
B3. Fluconazole-resistant Candida (a fungus)
B4. Extended spectrum beta-lactamase producing Enterobacteriaceae (ESBLs)
B5. Vancomycin-resistant Enterococcus (VRE)
B6. Multidrug-resistant Pseudomonas aeruginosa
B7. Drug-resistant non-typhoidal Salmonella
B8. Drug-resistant Salmonella enterica serovar Typhi
B9. Drug-resistant Shigella
B10. Methicillin-resistant Staphylococcus aureus (MRSA)
B11. Drug-resistant Streptococcus pneumoniae
B12. Drug-resistant tuberculosis
C. Concerning threats
C1. Vancomycin-resistant Staphylococcus aureus (VRSA)
C2. Erythromycin-resistant group A Streptococcus
C3. Clindamycin-resistant group B Streptococcus

The above are according to: Antibiotic Resistance Threats in the United States, 2013

Supplementary Table 3: Literature survey of significant MetaCyc pathways implicated in CODA-LASSO (Supplementary Figures 19-23) analyses that were positively associated with covariates.

No.	Pathways ID	Pathways Description	Covariate with Positive Association	Expected Taxonomic Range	Significance	References
1.	HISTSYN−PWY	L-histidine biosynthesis	BW_Mean, Day, FCR, FI	*Escherichia coli, Salmonella enterica enterica serovar Typhimurium*	Basic regulatory metabolic pathway in *E. coli* and *S. typhimurium.* L-Histidine being an essential amino acid provide relieve from heat stress in poultry.	(Chowdhury et al., 2021; Kulis-Horn et al., 2014; Malykh et al., 2018)
2.	PWY-7221	Guanosine ribonucleotides *de novo* biosynthesis	BW_Mean, Day, FI	*Escherichia coli, Salmonella enterica enterica serovar Typhimurium*	Guanine biosynthesis pathway of nucleotides generation for DNA replication and protein synthesis	(Kofoed et al., 2016)
3.	PWY0−1261	Peptidoglycan recycling-I	BW_Mean, FCR, FI, Gain	*Escherichia coli*	Peptidoglycan is a complex macromolecule, provides stability and protection by maintaining shape of cell wall. It can be broken down by peptidoglycan-cleaving enzymes during cell growth but recoverable in *E. coli* (cell wall recycling).	(Reith & Mayer, 2011)
4.	UDPNAGSYN−PWY	UDP-*N*-acetyl-D-glucosamine biosynthesis I	BW_Mean, FCR	*Escherichia coli*	Enzymes of this pathway are recently considered in advance and innovative antimicrobial preparations, by reactivating peptidoglycan biosynthesis resulting in increased sensitivity to antibiotics.	(M. Wang et al., 2020)
5.	PWY−6141	Archaetidylserine and archaetidylethanolamine biosynthesis	BW_Mean, FI, Gain	*Methanothermobacter thermautotrophicus, Methanothermobacter thermautotrophicus (Delta H)*	Inositol lipids are present in *Methanothermobacter thermautotrophicus*. In anaerobic receptacles using this *M. thermautotrophicus* (Delta H) methane can be produced from CO_2, CO and H_2.	(Chellapandi & Prathiviraj, 2020; Koga et al., 1998)
6.	ARGSYN−PWY	L-arginine biosynthesis I (via L-ornithine)	Day	*Escherichia coli, Salmonella enterica enterica serovar Typhimurium, Vibrionacae*	In birds L-arginine (amino acid) play role in protein generation and immune response. In *Enterobacteriaceae,* L-ornithine is a precursor of arginine and is generated by the intermediate compounds.	(Caldovic & Tuchman, 2003; Cunin et al., 1986; Lieboldt et al., 2016)
7.	PWY−6385	Peptidoglycan biosynthesis III (mycobacteria)	Day	*Mycobacterium leprae, Mycobacterium tuberculosis*	In *Mycobacteria* the basal structure (peptidoglycan / mucopeptide) is biosynthesized by this pathway. This pathway is same but with little unique end product in different bacteria like *E. coli* so the basal structure differs a bit. Studies shown the organized biogenesis of peptidoglycan in *Mycobacterium tuberculosis* make it resistant to anitibiotics.	(Crick et al., 2001; Kieser et al., 2015; Lavollay et al., 2008)
8.	PWY−7398	Coumarins biosynthesis (engineered)	Day, FI	*Escherichia coli*	De novo coumarin is derived when it is connected with the metabolism of bacteria (*E. coli*), by phenylpropanoid pathway coumarin derives. More than 1300 coumarins discovered and most of them have promised antimicrobial activity.	(Cheke et al., 2022; Lacy & O'Kennedy, 2004; Sahoo et al., 2021; S. M. Yang et al., 2015)

9.	PWY-1541	Superpathway of taurine degradation	BW_Mean, Day, FCR, FI, Gain	*Escherichia coli, Pseudomonas aeruginosa, Bilophila wadsworthia*	Commonly present in bacteria, involves various metabolic pathways in which amino acid like compounds (taurine) are transformed into sulfur complexes. In nervous system and circulatory system, taurine provokes growth of nerve tissues. *Pseudomonas aeruginosa* degrade taurine for energy, sulfur, carbon, and nitrogen.	(S. Han et al., 2020)
10.	PWY0−1533	Methylphosphonate degradation I	FCR	*Escherichia coli*	Phosphonate is common in all bacterial species, serving as phosphorous source and used in many antibiotics.	(Díaz-Mejía et al., 2009; Kamat et al., 2011)
11.	PYRIDNUCSYN−PWY	NAD *de novo* biosynthesis I	FCR	*Bacillus subtilis, Escherichia coli, Pseudomonas aeruginosa, Mycobacterium tuberculosis*	In redox reactions, Nicotinamide adenine dinucleotide (NAD) and nicotinamide adenine dinucleotide phosphate (NADP) are important for the generation of thiazole/oxazole rings from peptides. This is critical for narrow spectrum antibiotic activity.	(Andreoli et al., 1963; Baquero et al., 2022; Begley et al., 2001; Chandler et al., 1970)
12.	PWY−5840	Superpathway of menaquinol-7 biosynthesis	FI, Gain	*Bacillus subtilis, Staphylococcus aureus, Staphylococcus haemolyticus*	Menaquinones are sub-type of vitamin K_2, lipophilic in nature, produced by gut bacteria in animals.	(Bhattacharyya et al., 1997; Liao et al., 2021; Meganathan, 2001)
13.	OANTIGEN−PWY	*O*-antigen building blocks biosynthesis	FCR, FI, Gain	*Escherichia coli*	O-antigens are recurring oligosaccharides (galactose, N-acetylglucosamine, glucose and rhamnose) attached to main core in *E. coli*, served as a vaccine candidate.	(Aguirrezabalaga et al., 2000; S. Harris et al., 2018; Nishi et al., 2021; Stevenson et al., 1994)
14.	PWY−7187	Pyrimidine deoxyribonucleotides *de novo* biosynthesis II	BW_Mean, Day, FCR, FI, Gain	*Escherichia coli, Salmonella enterica enterica serovar Typhimurium, Mycobacterium tuberculosis*	Pyrimidine ribonucleotide is converted into respective deoxyribonucleotide with the help of ribonucleotide reductase enzyme (important in DNA replication/repair).	(Garavaglia et al., 2012; Schultheisz et al., 2011)

Supplementary Table 4: Literature survey of significant MetaCyc pathways implicated in CODA-LASSO (Supplementary Figures 19-23) analyses that were negatively associated with covariates.

Sr. No.	Pathways ID	Pathways Description	Covariate with Negative Association	Expected Taxonomic Range	Significance	Literature
1.	PWY−5188	Tetrapyrrole biosynthesis I (from glutamate)	BW_Mean, Day, FCR, FI, Gain	*Escherichia coli, Salmonella enterica enterica serovar Typhimurium*	It begins with glutamate in *E. coli* and other bacterial species leads to biosynthesis of variety of cofactors (heme, cobalamine, chlorophyll) which act as metal binding factor in various protein structures. If the heme synthesis is blocked then microbe will be sensitive to antimicrobials otherwise may be resistant.	(Bryant et al., 2020; Nardella et al., 2019; Schauer et al., 2002; Woodard & Dailey, 1995)
2.	1CMET2−PWY	Folate transformations III (*E. coli*)	BW_Mean, Day, FI, Gain	*Escherichia coli*	Glutamate, aminobenzoate and pterin fractions compose the folate. The tetrahydrofolate (vitamin B9) directly involves in biosynthesis of vital cofactors in *E. coli*.	(Abeysinghe & Kohen, 2015; de Crécy-Lagard et al., 2007; Rahman et al., 2020)
3.	NAD−BIOSYNT HESIS−II	NAD Biosynthesis	BW_Mean, Day, FI, Gain	*Escherichia coli*	Nicotinamide adenine dinucleotide (NAD) is a coenzyme vital for the metabolism in bacteria. Present in all organisms, it consists of dinucleotide linked over their phosphate groups. Its synthesis is dependent on various intermediate by products and steps essential in the metabolism.	(Belenky et al., 2007; Leonardo et al., 1996; X. Wang et al., 2017)
4.	PWY−6122	5-aminoimidazole ribonucleotide biosynthesis II	BW_Mean, Day, FCR, FI	*Escherichia coli*	This pathway is studied in *E. coli* and is not present in all bacterial species. 5-amino-1(5-phospho-beta-D-ribosyl)-imidazole plays a key role as intermediate for the generation of purine nucleotides and thiamine.	(Bazurto et al., 2016)
5.	PWY−6277	Superpathway of 5-aminoimidazole ribonucleotide biosynthesis	BW_Mean, Day, FCR, FI	*Escherichia coli*	5-amino-1(5-phospho-beta-D-ribosyl)-imidazole plays a key role as intermediate for the generation of purine nucleotides and thiamine. It is synthesized in five major steps with help of enzymes it catalyzes the pathway. On 3rd step of this pathway, *E. coli* has two enzymes which catalyze<br>1) folate-dependent Phospho ribosylglycinamide formyl-transferase and<br>2) Phospho ribosylglycinamide formyl-transferase	(Ahmad et al., 2019; DeMartino et al., 2008; Nygaard & Smith, 1993)
6.	PWY−7400	L-arginine biosynthesis IV (archaebacteria)	Day	*Enterobacteriaceae, Vibrionacae, Myxococcus xanthus*	Biosynthesis of arginine is a complex process and connected with other pathways in organisms like *Enterobacteriaceae, Vibrionaceae* and other Gram negative bacterium (*Myxococcus xanthus*). The pathway in these organisms starts with the acetylation of the amino moiety of L-glutamate.	(Cunin et al., 1986; B. Z. Harris & Singer, 1998; Xu et al., 2000)

7.	PEPTIDOGLYCANSYN−PWY	Peptidoglycan biosynthesis I (*meso*-diaminopimelate containing)	Day	*Bacillus anthracis, Bacillus subtilis, Corynebacterium ammoniagenes, Corynebactrium diphtheriae, Corynebactrium glutamicum, Corynebactrium pseudotuberculosis, Escherichia coli, Pseudomonas aeruginosa*	Peptidoglycan which is common in gram positive and negative bacteria, during this pathway peptidoglycan is biosynthesized. This is six step pathway which occur in cytoplasm.	(Delcour et al., 1999; Di Guilmi et al., 1998; Sauvage et al., 2008)
8.	PWY−5686	UMP biosynthesis I	Day	*Escherichia coli*	In the end of 2 steps of *de novo* biosynthesis of pyrimidine nucleotides pathway, supported by a bi-functional enzyme (UMP synthase) in animal and plant, but in bacteria there are 2 separate proteins for that purpose.	(Bucurenci et al., 1998; Garavaglia et al., 2012; Serina et al., 1995)
9.	PWY−6121	5-aminoimidazole ribonucleotide biosynthesis I	Day	*Escherichia coli, Salmonella enterica enterica serovar Typhimurium*	This pathway is studied in *E. coli* and is not present in all bacterial species. 5-amino-1(5-phospho-beta-D-ribosyl)-imidazole plays a key role as intermediate for the generation of purine nucleotides and thiamine.	(Aiba & Mizobuchi, 1989; Bazurto et al., 2016)
10.	ASPASN−PWY	Superpathway of L-aspartate and L-asparagine biosynthesis	FCR	*Escherichia coli*	Two metabolic pathways attached with L-aspartate in *E. coli.* 1- oxaloacetate formation 2- the fumarate and succinate formation during growth on glucose (anaerobe). It also aids in the synthesis of threonine, pyrimidines and methionine.	(Anishkin et al., 2015; Ardalan et al., 2018; Birolo et al., 1999; Guest et al., 1984)
11.	PWY−5897	Superpathway of menaquinol-11 biosynthesis	FI, Gain	*Bacteroides fragilis, Prevotella intermedia, Prevotella melaninogenica, Prevotella oralis, Prevotella ruminicola*	Menaquinones are sub-type of vitamin K_2. Lipophilic in nature, produced by the bacteria in intestine of animals. Illustrated best in *Bacillus subtilis*. .	(Allaway et al., 2020; Bentley & Meganathan, 1982; Hiratsuka et al., 2008; Yildirim et al., 2021)
12.	NONOXIPENT−PWY	Pentose phosphate pathway (non-oxidative branch) I	Day, FI, Gain	*Escherichia coli*	This pathway in *E. coli*, as a way to breakdown the sugars (glucose or pentoses), but it also supplies the cell with intermediate products when breakdown vitamins, nucleotides and amino acids. It also has a deregulatory role in glycolysis, liver cancers, tumors of malignant nature (cancer of breast and lung).	(Jin & Zhou, 2019; Sprenger, 1995)
13.	GLCMANNANAUT−PWY	Superpathway of *N*-acetylglucosamine, *N*-acetylmannosamine and *N*-acetylneuraminate degradation	BW_Mean, Day, FI	*Clostrudium perfrigens, Escherichia coli*	Cell structure of *E. coli* have important amino sugars on its outer surface which can be carbon, nitrogen source. It is a residue of mucin and the mucin is the main component of mucus present in human gut.	(Plumbridge & Vimr, 1999; Raimondi et al., 2021)
14.	PWY−5898	Superpathway of menaquinol-12 biosynthesis	FI, Gain	*Prevotella brevis, Prevotella oralis, Prevotella ruminicola*	Menaquinones are sub-type of vitamin K_2. Lipophilic in nature, produced by the bacteria in intestine of animals. Illustrated best in *Bacillus subtilis*.	(Bhattacharyya et al., 1997; Liao et al., 2021)

Supplementary Table 5: Tukey's HSD post hoc results of piARGs contribution for pairwise comparison of days where the differences were significant. Where the two days are from the days where diets are different, the background color signifies which two diets these are with the colors matching those in Figure 6.

Comparison	Diff	Lwr	Upr	P.adj
03-06	0.04546212	0.01353218	0.0773921	3.55E-05
03-07	0.05144303	0.01951309	0.083373	5.75E-07
03-08	0.05558426	0.02365432	0.0875142	2.48E-08
03-09	0.06528299	0.03335304	0.0972129	4.57E-11
03-11	0.06581762	0.02444928	0.107186	9.16E-07
03-12	0.06819573	0.03626579	0.1001257	3.98E-11
03-13	0.05085706	0.01892712	0.082787	8.80E-07
03-14	0.04523883	0.01330888	0.0771688	4.11E-05
03-15	0.05400923	0.01600499	0.0920135	3.76E-05
03-16	0.05038941	0.01238517	0.0883936	0.00025
03-17	0.05248827	0.02055833	0.0844182	2.66E-07
03-18	0.03729429	0.00536434	0.0692242	0.00418
03-19	0.04949604	0.0175661	0.081426	2.32E-06
03-21	0.03552978	0.00204136	0.0690182	0.02174
03-22	0.03263558	0.00070563	0.0645655	0.03733
03-25	0.04149932	0.00956937	0.0734293	0.00041
03-26	0.05835267	0.02642273	0.0902826	2.71E-09
03-29	0.05264257	0.02071262	0.0845725	2.37E-07
03-33	0.04947123	0.01754129	0.0814012	2.37E-06
03-34	0.04383651	0.01190657	0.0757665	9.98E-05
04-06	0.03476652	0.00283657	0.0666965	0.01446
04-07	0.04074743	0.00881748	0.0726774	0.00063
04-08	0.04488866	0.01295871	0.0768186	5.14E-05
04-09	0.05458738	0.02265743	0.0865173	5.40E-08
04-11	0.05512201	0.01375367	0.0964904	0.00022
04-12	0.05750013	0.02557018	0.0894301	5.40E-09
04-13	0.04016146	0.00823151	0.0720914	0.00089
04-14	0.03454322	0.00261328	0.0664732	0.01605
04-15	0.04331362	0.00530938	0.0813179	0.0066
04-16	0.0396938	0.00168957	0.077698	0.02746
04-17	0.04179266	0.00986272	0.0737226	0.00035
04-19	0.03880043	0.00687049	0.0707304	0.00188
04-26	0.04765707	0.01572712	0.079587	8.29E-06
04-29	0.04194696	0.01001701	0.0738769	0.00032
04-33	0.03877563	0.00684568	0.0707056	0.00191
04-34	0.03314091	0.00121096	0.0650709	0.03007
05-06	0.04110396	0.00917401	0.0730339	0.00052
05-07	0.04708487	0.01515492	0.0790148	1.22E-05
05-08	0.0512261	0.01929615	0.083156	6.73E-07
05-09	0.06092482	0.02899488	0.0928548	3.47E-10
05-11	0.06145946	0.02009112	0.1028278	9.62E-06
05-12	0.06383757	0.03190762	0.0957675	6.32E-11

05-13	0.0464989	0.01456895	0.0784288	1.80E-05
05-14	0.04088067	0.00895072	0.0728106	0.00059
05-15	0.04965106	0.01164682	0.0876553	0.00036
05-16	0.04603125	0.00802701	0.0840355	0.00202
05-17	0.04813011	0.01620016	0.0800601	6.01E-06
05-18	0.03293613	0.00100618	0.0648661	0.03285
05-19	0.04513788	0.01320793	0.0770678	4.38E-05
05-25	0.03714115	0.00521121	0.0690711	0.00452
05-26	0.05399451	0.02206456	0.0859245	8.52E-08
05-29	0.0482844	0.01635446	0.0802143	5.40E-06
05-33	0.04511307	0.01318312	0.077043	4.45E-05
05-34	0.03947835	0.0075484	0.0714083	0.0013
06-35	-0.0345762	-0.0680646	-0.0010878	0.03241
07-35	-0.0405571	-0.0740456	-0.0070687	0.00202
08-20	-0.0319406	-0.0638706	-1.07E-05	0.04978
08-30	-0.0325456	-0.0644755	-0.0006156	0.03877
08-31	-0.0322972	-0.0642271	-0.0003673	0.043
08-35	-0.0446984	-0.0781868	-0.01121	0.00021
09-10	-0.0382663	-0.0717547	-0.0047779	0.0063
09-20	-0.0416393	-0.0735693	-0.0097094	0.00038
09-22	-0.0326474	-0.0645774	-0.0007175	0.03714
09-23	-0.0359498	-0.0678797	-0.0040199	0.00821
09-24	-0.0375063	-0.0694362	-0.0055763	0.00374
09-28	-0.0389364	-0.0708663	-0.0070064	0.00175
09-30	-0.0422443	-0.0741742	-0.0103144	0.00026
09-31	-0.0419959	-0.0739259	-0.010066	0.00031
09-32	-0.0387598	-0.0706898	-0.0068299	0.00192
09-35	-0.0543971	-0.0878855	-0.0209087	4.21E-07
10-12	0.04117905	0.00769064	0.0746675	0.00146
11-12	-0.042174	-0.0835423	-0.0008056	0.03867
30-30	-0.0427789	-0.0841473	-0.0014106	0.0317
30-31	-0.0425306	-0.0838989	-0.0011622	0.03442
11-35	-0.0549317	-0.0975145	-0.012349	0.00049
12-20	-0.0445521	-0.076482	-0.0126221	6.37E-05
12-22	-0.0355602	-0.0674901	-0.0036302	0.00992
12-23	-0.0388625	-0.0707925	-0.0069326	0.00182
12-24	-0.040419	-0.0723489	-0.0084891	0.00077
12-28	-0.0418491	-0.0737791	-0.0099192	0.00033
12-30	-0.045157	-0.077087	-0.0132271	4.33E-05
12-31	-0.0449087	-0.0768386	-0.0129787	5.07E-05
12-32	-0.0416726	-0.0736025	-0.0097426	0.00037
12-35	-0.0573098	-0.0907983	-0.0238214	5.17E-08
13-35	-0.0399712	-0.0734596	-0.0064828	0.00272
14-35	-0.0343529	-0.0678414	-0.0008645	0.0355
15-35	-0.0431233	-0.082446	-0.0038006	0.01286
16-35	-0.0395035	-0.0788262	-0.0001808	0.04709

17-35	-0.0416024	-0.0750908	-0.008114	0.00117
19-35	-0.0386102	-0.0720986	-0.0051217	0.00534
20-26	0.03470902	0.00277907	0.066639	0.01486
26-28	-0.0320061	-0.063936	-7.61E-05	0.04847
26-30	-0.035314	-0.0672439	-0.003384	0.01117
26-31	-0.0350656	-0.0669956	-0.0031357	0.01257
26-35	-0.0474668	-0.0809552	-0.0139784	4.06E-05
29-35	-0.0417567	-0.0752451	-0.0082683	0.00108
33-35	-0.0385853	-0.0720738	-0.0050969	0.0054

3 References

Abeysinghe, T., & Kohen, A. (2015). Role of long-range protein dynamics in different thymidylate synthase catalyzed reactions. *International Journal of Molecular Sciences*, *16*(4), 7304–7319. https://doi.org/10.3390/ijms16047304

Aguirrezabalaga, I., Olano, C., Allende, N., Rodriguez, L., Braña, A. F., Méndez, C., & Salas, J. A. (2000). Identification and expression of genes involved in biosynthesis of L- oleandrose and its intermediate L-olivose in the oleandomycin producer Streptomyces antibioticus. *Antimicrobial Agents and Chemotherapy*, *44*(5), 1266–1275. https://doi.org/10.1128/AAC.44.5.1266-1275.2000

Ahmad, S., Wang, B., Walker, M. D., Tran, H. K. R., Stogios, P. J., Savchenko, A., Grant, R. A., McArthur, A. G., Laub, M. T., & Whitney, J. C. (2019). An interbacterial toxin inhibits target cell growth by synthesizing (p)ppApp. *Nature*, *575*(7784), 674–678. https://doi.org/10.1038/s41586-019-1735-9

Aiba, A., & Mizobuchi, K. (1989). Nucleotide sequence analysis of genes purH and purD involved in the de novo purine nucleotide biosynthesis of Escherichia coli. *Journal of Biological Chemistry*, *264*(35), 21239–21246. https://doi.org/10.1016/s0021-9258(19)30072-9

Allaway, D., Haydock, R., Lonsdale, Z. N., Deusch, O. D., O'Flynn, C., & Hughes, K. R. (2020). Rapid reconstitution of the fecal microbiome after extended diet-induced changes indicates a stable gut microbiome in healthy adult dogs. *Applied and Environmental Microbiology*, *86*(13). https://doi.org/10.1128/AEM.00562-20/SUPPL_FILE/AEM.00562-20-SD003.XLSX

Allende, A., Koutsoumanis, K., Allende, A., Alvarez-Ordóñez, A., Bolton, D., Bover-Cid, S., Chemaly, M., Davies, R., De Cesare, A., Herman, L., Hilbert, F., Lindqvist, R., Nauta, M., Ru, G., Simmons, M., Skandamis, P., Suffredini, E., Andersson, D. I., Bampidis, V., … Peixe, L. (2021). Maximum levels of cross-contamination for 24 antimicrobial active substances in non-target feed.</br>Part 2: Aminoglycosides/aminocyclitols: apramycin, paromomycin, neomycin and spectinomycin. *EFSA Journal*, *19*(10), e06853. https://doi.org/10.2903/J.EFSA.2021.6853

Anaerofustis. (n.d.). Retrieved November 21, 2022, from https://www.namesforlife.com/10.1601/nm.8462

Andreoli, A. J., Ikeda, M., Nishizuka, Y., & Hayaishi, O. (1963). Quinolinic acid: A precursor to nicotinamide adenine dinucleotide in Escherichia coli. *Biochemical and Biophysical Research Communications*, *12*(2), 92–97. https://doi.org/10.1016/0006-291X(63)90241-9

Anishkin, A., Vanegas, J. M., Rogers, D. M., Lorenzi, P. L., Chan, W. K., Purwaha, P., Weinstein, J. N., Sukharev, S., & Rempe, S. B. (2015). Catalytic Role of the Substrate Defines Specificity of Therapeutic l-Asparaginase. *Journal of Molecular Biology*, *427*(17), 2867–2885. https://doi.org/10.1016/j.jmb.2015.06.017

Antonissen, G., Eeckhaut, V., Van Driessche, K., Onrust, L., Haesebrouck, F., Ducatelle, R., Moore, R. J., & Van Immerseel, F. (2016). Microbial shifts associated with necrotic enteritis. *Avian Pathology : Journal of the W.V.P.A*, *45*(3), 308–312. https://doi.org/10.1080/03079457.2016.1152625

Ardalan, N., Mirzaie, S., Sepahi, A. A., & Khavari-Nejad, R. A. (2018). Novel mutant of Escherichia coli asparaginase II to reduction of the glutaminase activity in treatment of acute lymphocytic leukemia by molecular dynamics simulations and QM-MM studies. *Medical Hypotheses*, *112*, 7–17. https://doi.org/10.1016/j.mehy.2018.01.004

Ash, C., Priest, F. G., & Collins, M. D. (1993). Molecular identification of rRNA group 3 bacilli (Ash, Farrow, Wallbanks and Collins) using a PCR probe test. Proposal for the creation of a new genus Paenibacillus. *Antonie van Leeuwenhoek*, *64*(3–4), 253–260. https://doi.org/10.1007/BF00873085

Awad, W. A., Molnár, A., Aschenbach, J. R., Ghareeb, K., Khayal, B., Hess, C., Liebhart, D., Dublecz, K., & Hess, M. (2015). Campylobacter infection in chickens modulates the intestinal epithelial barrier function. *Innate Immunity*, *21*(2), 151–160. https://doi.org/10.1177/1753425914521648

Baquero, F., del Campo, R., & Martínez, J. L. (2022). Interventions in Nicotinamide Adenine Dinucleotide Metabolism, the Intestinal Microbiota and Microcin Peptide Antimicrobials. *Frontiers in Molecular Biosciences*, *9*. https://doi.org/10.3389/FMOLB.2022.861603

Barnes, E. M. (1979). The intestinal microflora of poultry and game birds during life and after storage. Address of the president of the Society for Applied Bacteriology delivered at a meeting of the society on 10 January 1979. *The Journal of Applied Bacteriology*, *46*(3), 407–419. https://doi.org/10.1111/J.1365-2672.1979.TB00838.X

Bazurto, J. V., Farley, K. R., & Downs, D. M. (2016). An unexpected route to an essential cofactor: Escherichia coli relies on threonine for thiamine biosynthesis. *MBio*, *7*(1). https://doi.org/10.1128/mBio.01840-15

Begley, T. P., Kinsland, C., Mehl, R. A., Osterman, A., & Dorrestein, P. (2001). The biosynthesis of nicotinamide adenine dinucleotides in bacteria. *Vitamins and Hormones*, *61*, 103–119. https://doi.org/10.1016/s0083-6729(01)61003-3

Belenky, P., Bogan, K. L., & Brenner, C. (2007). NAD+ metabolism in health and disease. *Trends in Biochemical Sciences*, *32*(1), 12–19. https://doi.org/10.1016/j.tibs.2006.11.006

Bentley, R., & Meganathan, R. (1982). Biosynthesis of vitamin K (menaquinone) in bacteria. *Microbiological Reviews*, *46*(3), 241–280. https://doi.org/10.1128/mmbr.46.3.241-280.1982

Bhattacharyya, D. K., Kwon, O., & Meganathan, R. (1997). Vitamin K2 (menaquinone) biosynthesis in Escherichia coli: Evidence for the presence of an essential histidine residue in o-succinylbenzoyl coenzyme A synthetase. *Journal of Bacteriology*, *179*(19), 6061–6065. https://doi.org/10.1128/jb.179.19.6061-6065.1997

Biddle, A., Stewart, L., Blanchard, J., & Leschine, S. (2013). Untangling the Genetic Basis of Fibrolytic Specialization by Lachnospiraceae and Ruminococcaceae in Diverse Gut Communities. *Diversity 2013, Vol. 5, Pages 627-640*, *5*(3), 627–640. https://doi.org/10.3390/D5030627

Birolo, L., Malashkevich, V. N., Capitani, G., De Luca, F., Moretta, A., Jansonius, J. N., & Marino, G. (1999). Functional and structural analysis of cis-proline mutants of Escherichia coli aspartate aminotransferase. *Biochemistry*, *38*(3), 905–913. https://doi.org/10.1021/bi981467d

Broom, L. J. (2018). Gut barrier function: Effects of (antibiotic) growth promoters on key barrier components and associations with growth performance. *Poultry Science*, *97*(5), 1572–1578. https://doi.org/10.3382/PS/PEY021

Bryant, D. A., Hunter, C. N., & Warren, M. J. (2020). Biosynthesis of the modified tetrapyrroles—the pigments of life. *The Journal of Biological Chemistry*, *295*(20), 6888. https://doi.org/10.1074/JBC.REV120.006194

Bucurenci, N., Serina, L., Zaharia, C., Landais, S., Danchin, A., & Barzu, O. (1998). Mutational Analysis of UMP Kinase from Escherichia coli. *Journal of Bacteriology*, *180*(3), 473. https://doi.org/10.1128/JB.180.3.473-477.1998

Bui, T. P. N., Ritari, J., Boeren, S., De Waard, P., Plugge, C. M., & De Vos, W. M. (2015). Production of butyrate from lysine and the Amadori product fructoselysine by a human gut commensal. *Nature Communications 2015 6:1*, *6*(1), 1–10. https://doi.org/10.1038/ncomms10062

Caldovic, L., & Tuchman, M. (2003). N-acetylglutamate and its changing role through evolution. *Biochemical Journal*, *372*(2), 279–290. https://doi.org/10.1042/BJ20030002

Calle, M. L., & Susin, A. (2022). coda4microbiome: compositional data analysis for microbiome studies. *BioRxiv*, 2022.06.09.495511. https://doi.org/10.1101/2022.06.09.495511

Carrasco, J. M. D., Casanova, N. A., & Miyakawa, M. E. F. (2019). Microbiota, Gut Health and Chicken Productivity: What Is the Connection? *Microorganisms*, *7*(10). https://doi.org/10.3390/MICROORGANISMS7100374

Chandler, J. L. R., Gholson, R. K., & Scott, T. A. (1970). Studies on the de novo biosynthesis of NAD in Escherichia coli I. Labelling patterns from precursors. *BBA - General Subjects*, *222*(2), 523–526. https://doi.org/10.1016/0304-4165(70)90145-5

Chateau, N., Castellanos, I., & Deschamps, A. M. (1993). Distribution of pathogen inhibition in the Lactobacillus isolates of a commercial probiotic consortium. *The Journal of Applied Bacteriology*, *74*(1), 36–40. https://doi.org/10.1111/J.1365-2672.1993.TB02993.X

Chattopadhyay, M. K. (2014). Use of antibiotics as feed additives: A burning question. *Frontiers in Microbiology*, *5*(JULY), 334. https://doi.org/10.3389/FMICB.2014.00334/BIBTEX

Cheke, R. S., Patel, H. M., Patil, V. M., Ansari, I. A., Ambhore, J. P., Shinde, S. D., Kadri, A., Snoussi, M., Adnan, M., Kharkar, P. S., Pasupuleti, V. R., & Deshmukh, P. K. (2022). Molecular Insights into Coumarin Analogues as Antimicrobial Agents: Recent Developments in Drug Discovery. *Antibiotics*, *11*(5). https://doi.org/10.3390/ANTIBIOTICS11050566

Chellapandi, P., & Prathiviraj, R. (2020). Methanothermobacter thermautotrophicus strain ΔH as a potential microorganism for bioconversion of CO2 to methane. *Journal of CO2 Utilization*, *40*, 101210. https://doi.org/10.1016/J.JCOU.2020.101210

Chen, H. L., Zhao, X. Y., Zhao, G. X., Huang, H. Bin, Li, H. R., Shi, C. W., Yang, W. T., Jiang, Y. L., Wang, J. Z., Ye, L. P., Zhao, Q., Wang, C. F., & Yang, G. L. (2020). Dissection of the cecal microbial community in chickens after Eimeria tenella infection. *Parasites and Vectors*, *13*(1), 1–15. https://doi.org/10.1186/S13071-020-3897-6/FIGURES/11

Chen, Y. ran, Zheng, H. min, Zhang, G. xia, Chen, F. lan, Chen, L. dan, & Yang, Z. cong. (2020). High Oscillospira abundance indicates constipation and low BMI in the Guangdong Gut Microbiome Project. *Scientific Reports*, *10*(1). https://doi.org/10.1038/S41598-020-66369-Z

Chika, E., Charles, E., Ifeanyichukwu, I., & Michael, A. (2018). First Detection of FOX-1 AmpC β-lactamase Gene Expression Among Escherichia coli Isolated from Abattoir Samples in Abakaliki, Nigeria. *Oman Medical Journal*, *33*(3), 243. https://doi.org/10.5001/OMJ.2018.44

Chowdhury, V. S., Han, G., Eltahan, H. M., Haraguchi, S., Gilbert, E. R., Cline, M. A., Cockrem, J. F., Bungo, T., & Furuse, M. (2021). Potential Role of Amino Acids in the Adaptation of Chicks and Market-Age Broilers to Heat Stress. *Frontiers in Veterinary Science*, *7*, 1109. https://doi.org/10.3389/FVETS.2020.610541/BIBTEX

Clavijo, V., Morales, T., Vives-Flores, M. J., & Reyes Muñoz, A. (2022). The gut microbiota of chickens in a commercial farm treated with a Salmonella phage cocktail. *Scientific Reports 2022 12:1*, *12*(1), 1–16. https://doi.org/10.1038/s41598-021-04679-6

Connelly, S., Fanelli, B., Hasan, N. A., Colwell, R. R., & Kaleko, M. (2019). Oral Metallo-Beta-Lactamase Protects the Gut Microbiome From Carbapenem-Mediated Damage and Reduces Propagation of Antibiotic Resistance in Pigs. *Frontiers in Microbiology*, *10*(FEB). https://doi.org/10.3389/FMICB.2019.00101

Costa, M. C., Bessegatto, J. A., Alfieri, A. A., Weese, J. S., Filho, J. A. B., & Oba, A. (2017). Different antibiotic growth promoters induce specific changes in the cecal microbiota membership of broiler chicken. *PLOS ONE*, *12*(2), e0171642. https://doi.org/10.1371/JOURNAL.PONE.0171642

Crick, D. C., Mahapatra, S., & Brennan, P. J. (2001). Biosynthesis of the arabinogalactan-peptidoglycan complex of Mycobacterium tuberculosis. *Glycobiology*, *11*(9). https://doi.org/10.1093/glycob/11.9.107R

Cunin, R., Glansdorff, N., Pierard, A., & Stalon, V. (1986). Biosynthesis and metabolism of arginine in bacteria. *Microbiological Reviews*, *50*(3), 314–352. https://doi.org/10.1128/mmbr.50.3.314-352.1986

Danylec, N., Göbl, A., Stoll, D. A., Hetzer, B., Kulling, S. E., & Huch, M. (2018). Rubneribacter badeniensis gen. Nov., sp. Nov. And enteroscipio rubneri gen. Nov., sp. Nov., new members of the Eggerthellaceae isolated from human faeces. *International Journal of Systematic and Evolutionary Microbiology*, *68*(5), 1533–1540. https://doi.org/10.1099/IJSEM.0.002705/CITE/REFWORKS

Darcy, J. L., Amend, A. S., Swift, S. O. I., Sommers, P. S., & Lozupone, C. A. (2022). specificity: an R package for analysis of feature specificity to environmental and higher dimensional variables, applied to microbiome species data. *BioRxiv*, 2021.11.06.467582. https://doi.org/10.1101/2021.11.06.467582

de Crécy-Lagard, V., El Yacoubi, B., de la Garza, R. D., Noiriel, A., & Hanson, A. D. (2007). Comparative genomics of bacterial and plant folate synthesis and salvage: Predictions and validations. *BMC Genomics*, *8*. https://doi.org/10.1186/1471-2164-8-245

Del Fiol, F. S., Balcão, V. M., Barberato-Fillho, S., Lopes, L. C., & Bergamaschi, C. C. (2018). Obesity: A New Adverse Effect of Antibiotics? *Frontiers in Pharmacology*, *9*. https://doi.org/10.3389/FPHAR.2018.01408

Delcour, J., Ferain, T., Deghorain, M., Palumbo, E., & Hols, P. (1999). The biosynthesis and functionality of the cell-wall of lactic acid bacteria. *Antonie van Leeuwenhoek, International Journal of General and Molecular Microbiology*, *76*(1–4), 159–184. https://doi.org/10.1023/A:1002089722581

DeMartino, J. K., Hwang, I., Connelly, S., Wilson, I. A., & Boger, D. L. (2008). Asymmetric synthesis of inhibitors of glycinamide ribonucleotide transformylase. *Journal of Medicinal Chemistry*, *51*(17), 5441–5448. https://doi.org/10.1021/jm800555h

Dewhirst, F. E., Paster, B. J., Tzellas, N., Coleman, B., Downes, J., Spartt, D. A., & Wade, W. G. (2001). Characterization of novel human oral isolates and cloned 16S rDNA sequences that fall in the family Coriobacteriaceae: description of olsenella gen. nov., reclassification of Lactobacillus uli as Olsenella uli comb. nov. and description of Olsenella profusa sp. nov.

International Journal of Systematic and Evolutionary Microbiology, *51*(Pt 5), 1797–1804. https://doi.org/10.1099/00207713-51-5-1797

Di Guilmi, A. M., Mouz, N., Andrieu, J. P., Hoskins, J., Jaskunas, S. R., Gagnon, J., Dideberg, O., & Vernet, T. (1998). Identification, purification, and characterization of transpeptidase and glycosyltransferase domains of Streptococcus pneumoniae penicillin-binding protein 1a. *Journal of Bacteriology*, *180*(21), 5652–5659. https://doi.org/10.1128/jb.180.21.5652-5659.1998

Diarra, M. S., & Malouin, F. (2014). Antibiotics in Canadian poultry productions and anticipated alternatives. *Frontiers in Microbiology*, *5*(JUN), 282. https://doi.org/10.3389/FMICB.2014.00282/BIBTEX

Díaz-Mejía, J. J., Babu, M., & Emili, A. (2009). Computational and experimental approaches to chart the Escherichia coli cell-envelope-associated proteome and interactome. *FEMS Microbiology Reviews*, *33*(1), 66–97. https://doi.org/10.1111/j.1574-6976.2008.00141.x

Durre, P., & Andreesen, J. R. (1983). Purine and glycine metabolism by purinolytic clostridia. *Journal of Bacteriology*, *154*(1), 192. https://doi.org/10.1128/JB.154.1.192-199.1983

Eeckhaut, V., Wang, J., Van Parys, A., Haesebrouck, F., Joossens, M., Falony, G., Raes, J., Ducatelle, R., & Van Immerseel, F. (2016). The Probiotic Butyricicoccus pullicaecorum Reduces Feed Conversion and Protects from Potentially Harmful Intestinal Microorganisms and Necrotic Enteritis in Broilers. *Frontiers in Microbiology*, *7*(SEP). https://doi.org/10.3389/FMICB.2016.01416

Ekim, B., Calik, A., Ceylan, A., & Saçaklı, P. (2020). Effects of Paenibacillus xylanexedens on growth performance, intestinal histomorphology, intestinal microflora, and immune response in broiler chickens challenged with Escherichia coli K88. *Poultry Science*, *99*(1), 214. https://doi.org/10.3382/PS/PEZ460

El-Adawy, H., Bocklisch, H., Neubauer, H., Hafez, H. M., & Hotzel, H. (2018). Identification, differentiation and antibiotic susceptibility of Gallibacterium isolates from diseased poultry. *Irish Veterinary Journal*, *71*(1), 1–10. https://doi.org/10.1186/S13620-018-0116-2/FIGURES/3

Emami, N. K., Schreier, L. L., Greene, E., Tabler, T., Orlowski, S. K., Anthony, N. B., Proszkowiec-Weglarz, M., & Dridi, S. (2022). Ileal microbial composition in genetically distinct chicken lines reared under normal or high ambient temperatures. *Animal Microbiome*, *4*(1). https://doi.org/10.1186/S42523-022-00183-Y

Farkas, V., Csitári, G., Menyhárt, L., Such, N., Pál, L., Husvéth, F., Rawash, M. A., Mezőlaki, Á., & Dublecz, K. (2022). Microbiota Composition of Mucosa and Interactions between the Microbes of the Different Gut Segments Could Be a Factor to Modulate the Growth Rate of Broiler Chickens. *Animals*, *12*(10), 1296. https://doi.org/10.3390/ANI12101296/S1

Feighner, S. D., & Dashkevicz, M. P. (1987). Subtherapeutic levels of antibiotics in poultry feeds and their effects on weight gain, feed efficiency, and bacterial cholyltaurine hydrolase activity. *Applied and Environmental Microbiology*, *53*(2), 331–336. https://doi.org/10.1128/AEM.53.2.331-336.1987

Feng, P., Li, Q., Sun, H., Gao, J., Ye, X., Tao, Y., Tian, Y., & Wang, P. (2022). Effects of fulvic acid on growth performance, serum index, gut microbiota, and metabolites of Xianju yellow chicken. *Frontiers in Nutrition*, *9*. https://doi.org/10.3389/FNUT.2022.963271/FULL

Ferrario, C., Alessandri, G., Mancabelli, L., Gering, E., Mangifesta, M., Milani, C., Lugli, G. A., Viappiani, A., Duranti, S., Turroni, F., Ossiprandi, M. C., Hiyashi, R., Mackie, R., van Sinderen,

D., & Ventura, M. (2017). Untangling the cecal microbiota of feral chickens by culturomic and metagenomic analyses. *Environmental Microbiology*, *19*(11), 4771–4783. https://doi.org/10.1111/1462-2920.13943

Finn, D. R., Yu, J., Ilhan, Z. E., Fernandes, V. M. C., Penton, C. R., Krajmalnik-Brown, R., Garcia-Pichel, F., & Vogel, T. M. (2020). MicroNiche: an R package for assessing microbial niche breadth and overlap from amplicon sequencing data. *FEMS Microbiology Ecology*, *96*(8), 131. https://doi.org/10.1093/FEMSEC/FIAA131

Furlong, M., Deming-Halverson, S., & Sandler, D. P. (2019). Chronic antibiotic use during adulthood and weight change in the Sister Study. *PLoS ONE*, *14*(5). https://doi.org/10.1371/JOURNAL.PONE.0216959

Garavaglia, M., Rossi, E., & Landini, P. (2012). The Pyrimidine Nucleotide Biosynthetic Pathway Modulates Production of Biofilm Determinants in Escherichia coli. *PLOS ONE*, *7*(2), e31252. https://doi.org/10.1371/JOURNAL.PONE.0031252

Garner, M. R., Flint, J. F., & Russell, J. B. (2002). Allisonella histaminiformans gen. nov., sp. nov.: A Novel Bacterium that Produces Histamine, Utilizes Histidine as its Sole Energy Source, and Could Play a Role in Bovine and Equine Laminitis. *Systematic and Applied Microbiology*, *25*(4), 498–506. https://doi.org/10.1078/07232020260517625

Gong, H., Yang, Z., Celi, P., Yan, L., Ding, X., Bai, S., Zeng, Q., Xu, S., Su, Z., Zhuo, Y., Zhang, K., & Wang, J. (2021). Effect of benzoic acid on production performance, egg quality, intestinal morphology, and cecal microbial community of laying hens. *Poultry Science*, *100*(1), 196–205. https://doi.org/10.1016/J.PSJ.2020.09.065

Guest, J. R., Roberts, R. E., & Wilde, R. J. (1984). Cloning of the aspartase gene (aspA) of Escherichia coli. *Journal of General Microbiology*, *130*(5), 1271–1278. https://doi.org/10.1099/00221287-130-5-1271

Han, H., Zhou, Y., Liu, Q., Wang, G., Feng, J., & Zhang, M. (2021). Effects of Ammonia on Gut Microbiota and Growth Performance of Broiler Chickens. *Animals : An Open Access Journal from MDPI*, *11*(6). https://doi.org/10.3390/ANI11061716

Han, S., Zheng, Y., Yu, Z., Fu, Q., Lian, X., Wang, L., & Song, L. (2020). Comparative characterization of bacterial communities in digestive glands of Crassostrea gigas fed with different microalgal diets. *Invertebrate Survival Journal*, *17*(1), 51–62. https://doi.org/10.25431/1824-307X/ISJ.V0I0.51-62

Harris, B. Z., & Singer, M. (1998). Identification and characterization of the Myxococcus xanthus argE gene. *Journal of Bacteriology*, *180*(23), 6412–6414. https://doi.org/10.1128/jb.180.23.6412-6414.1998

Harris, S., Piotrowska, M. J., Goldstone, R. J., Qi, R., Foster, G., Dobrindt, U., Madec, J. Y., Valat, C., Rao, F. V., & Smith, D. G. E. (2018). Variant O89 O-antigen of E. coli is associated with group 1 capsule loci and multidrug resistance. *Frontiers in Microbiology*, *9*(AUG), 2026. https://doi.org/10.3389/FMICB.2018.02026/BIBTEX

Harry, E. G., Mead, G. C., & Barnum, D. A. (1972). The intestinal flora of the chicken in the period 2 to 6 weeks of age, with particular reference to the anaerobic bacteria. *British Poultry Science*, *13*(3), 311–326. https://doi.org/10.1080/00071667208415953

Hartwich, K., Poehlein, A., & Daniel, R. (2012). The purine-utilizing bacterium Clostridium acidurici

9a: a genome-guided metabolic reconsideration. *PloS One*, *7*(12). https://doi.org/10.1371/JOURNAL.PONE.0051662

Hiratsuka, T., Furihata, K., Ishikawa, J., Yamashita, H., Itoh, N., Seto, H., & Dairi, T. (2008). An alternative menaquinone biosynthetic pathway operating in microorganisms. *Science*, *321*(5896), 1670–1673. https://doi.org/10.1126/science.1160446

Huang, Y., Lv, H., Song, Y., Sun, C., Zhang, Z., & Chen, S. (2021). Community composition of cecal microbiota in commercial yellow broilers with high and low feed efficiencies. *Poultry Science*, *100*(4), 100996. https://doi.org/10.1016/J.PSJ.2021.01.019

Ijaz, U. Z., Sivaloganathan, L., McKenna, A., Richmond, A., Kelly, C., Linton, M., Stratakos, A. C., Lavery, U., Elmi, A., Wren, B. W., Dorrell, N., Corcionivoschi, N., & Gundogdu, O. (2018). Comprehensive Longitudinal Microbiome Analysis of the Chicken Cecum Reveals a Shift From Competitive to Environmental Drivers and a Window of Opportunity for Campylobacter. *Frontiers in Microbiology*, *9*(OCT). https://doi.org/10.3389/FMICB.2018.02452

Jayanama, K., Phuphuakrat, A., Pongchaikul, P., Prombutara, P., Nimitphong, H., Reutrakul, S., & Sungkanuparph, S. (2022). Association between gut microbiota and prediabetes in people living with HIV. *Current Research in Microbial Sciences*, *3*, 100143. https://doi.org/10.1016/J.CRMICR.2022.100143

Jin, L., & Zhou, Y. (2019). Crucial role of the pentose phosphate pathway in malignant tumors. *Oncology Letters*, *17*(5), 4213. https://doi.org/10.3892/OL.2019.10112

Kamat, S. S., Williams, H. J., & Raushel, F. M. (2011). Intermediates in the transformation of phosphonates to phosphate by bacteria. *Nature*, *480*(7378), 570–573. https://doi.org/10.1038/nature10622

Kaplan, R. C., Wang, Z., Usyk, M., Sotres-Alvarez, D., Daviglus, M. L., Schneiderman, N., Talavera, G. A., Gellman, M. D., Thyagarajan, B., Moon, J. Y., Vázquez-Baeza, Y., McDonald, D., Williams-Nguyen, J. S., Wu, M. C., North, K. E., Shaffer, J., Sollecito, C. C., Qi, Q., Isasi, C. R., … Burk, R. D. (2019). Gut microbiome composition in the Hispanic Community Health Study/Study of Latinos is shaped by geographic relocation, environmental factors, and obesity. *Genome Biology*, *20*(1), 1–21. https://doi.org/10.1186/S13059-019-1831-Z/TABLES/6

Kempf, F., Menanteau, P., Rychlik, I., Kubasová, T., Trotereau, J., Virlogeux-Payant, I., Schaeffer, S., Schouler, C., Drumo, R., Guitton, E., & Velge, P. (2020). Gut microbiota composition before infection determines the Salmonella super- and low-shedder phenotypes in chicken. *Microbial Biotechnology*, *13*(5), 1611. https://doi.org/10.1111/1751-7915.13621

Khan, S., & Chousalkar, K. K. (2020). Short-term feeding of probiotics and synbiotics modulates caecal microbiota during Salmonella Typhimurium infection but does not reduce shedding and invasion in chickens. *Applied Microbiology and Biotechnology*, *104*(1), 319–334. https://doi.org/10.1007/S00253-019-10220-7/FIGURES/7

Kieser, K. J., Baranowski, C., Chao, M. C., Long, J. E., Sassetti, C. M., Waldor, M. K., Sacchettini, J. C., Ioerger, T. R., & Rubin, E. J. (2015). Peptidoglycan synthesis in Mycobacterium tuberculosis is organized into networks with varying drug susceptibility. *Proceedings of the National Academy of Sciences of the United States of America*, *112*(42), 13087–13092. https://doi.org/10.1073/PNAS.1514135112/SUPPL_FILE/PNAS.1514135112.SD02.XLS

Kläring, K., Hanske, L., Bui, N., Charrier, C., Blaut, M., Haller, D., Plugge, C. M., & Clavel, T. (2013). Intestinimonas butyriciproducens gen. nov., sp. nov., a butyrate-producing bacterium from the

mouse intestine. *International Journal of Systematic and Evolutionary Microbiology*, *63*(Pt 12), 4606–4612. https://doi.org/10.1099/IJS.0.051441-0

Kofoed, E. M., Yan, D., Katakam, A. K., Reichelt, M., Lin, B., Kim, J., Park, S., Date, S. V., Monk, I. R., Xu, M., Austin, C. D., Maurer, T., & Tan, M. W. (2016). De novo guanine biosynthesis but not the riboswitch-regulated purine salvage pathway is required for Staphylococcus aureus infection in vivo. *Journal of Bacteriology*, *198*(14), 2001–2015. https://doi.org/10.1128/JB.00051-16/SUPPL_FILE/ZJB999094109SO1.PDF

Koga, Y., Kyuragi, T., Nishihara, M., & Sone, N. (1998). Did archaeal and bacterial cells arise independently from noncellular precursors? A hypothesis stating that the advent of membrane phospholipid with enantiomeric glycerophosphate backbones caused the separation of the two lines of descent. *Journal of Molecular Evolution*, *46*(1), 54–63. https://doi.org/10.1007/PL00006283

Könönen, E., Conrads, G., & Nagy, E. (2015). Bacteroides, Porphyromonas, Prevotella, Fusobacterium, and Other Anaerobic Gram-Negative Rods. *Manual of Clinical Microbiology* , 967–993. https://doi.org/10.1128/9781555817381.CH54

Krysiak, K., Konkol, D., & Korczyński, M. (2021). Overview of the Use of Probiotics in Poultry Production. *Animals : An Open Access Journal from MDPI*, *11*(6). https://doi.org/10.3390/ANI11061620

Kulis-Horn, R. K., Persicke, M., & Kalinowski, J. (2014). Histidine biosynthesis, its regulation and biotechnological application in Corynebacterium glutamicum. *Microbial Biotechnology*, *7*(1), 5–25. https://doi.org/10.1111/1751-7915.12055

Kumar, S., Shang, Y., & Kim, W. K. (2019). Insight into dynamics of gut microbial community of broilers fed with fructooligosaccharides supplemented low calcium and phosphorus diets. *Frontiers in Veterinary Science*, *6*(MAR), 95. https://doi.org/10.3389/FVETS.2019.00095/FULL

Lacy, A., & O'Kennedy, R. (2004). Studies on coumarins and coumarin-related compounds to determine their therapeutic role in the treatment of cancer. *Current Pharmaceutical Design*, *10*(30), 3797–3811. http://www.ncbi.nlm.nih.gov/pubmed/15579072

Lavollay, M., Arthur, M., Fourgeaud, M., Dubost, L., Marie, A., Veziris, N., Blanot, D., Gutmann, L., & Mainardi, J. L. (2008). The peptidoglycan of stationary-phase Mycobacterium tuberculosis predominantly contains cross-links generated by L,D-transpeptidation. *Journal of Bacteriology*, *190*(12), 4360–4366. https://doi.org/10.1128/JB.00239-08

Leonardo, M. R., Dailly, Y., & Clark, D. P. (1996). Role of NAD in regulating the adhE gene of Escherichia coli. *Journal of Bacteriology*, *178*(20), 6013–6018. https://doi.org/10.1128/JB.178.20.6013-6018.1996

Li, Y., Zhang, L. L., Liu, L., Tian, Y. Q., Liu, X. F., Li, W. J., & Dai, Y. M. (2017). Paludicola psychrotolerans gen. nov., sp. nov., a novel psychrotolerant chitinolytic anaerobe of the family Ruminococcaceae. *International Journal of Systematic and Evolutionary Microbiology*, *67*(10), 4100–4103. https://doi.org/10.1099/IJSEM.0.002260

Li, Z., Wang, W., Liu, D., & Guo, Y. (2018). Effects of Lactobacillus acidophilus on the growth performance and intestinal health of broilers challenged with Clostridium perfringens. *Journal of Animal Science and Biotechnology*, *9*(1), 1–10. https://doi.org/10.1186/S40104-018-0243-3/TABLES/10

Liao, C., Ayansola, H., Ma, Y., Ito, K., Guo, Y., & Zhang, B. (2021). Advances in Enhanced Menaquinone-7 Production From Bacillus subtilis. *Frontiers in Bioengineering and Biotechnology*, *9*, 656. https://doi.org/10.3389/FBIOE.2021.695526/BIBTEX

Lieboldt, M. A., Frahm, J., Halle, I., Görs, S., Schrader, L., Weigend, S., Preisinger, R., Metges, C. C., Breves, G., & Dänicke, S. (2016). Metabolic and clinical response to Escherichia coli lipopolysaccharide in layer pullets of different genetic backgrounds supplied with graded dietary L-arginine. *Poultry Science*, *95*(3), 595–611. https://doi.org/10.3382/PS/PEV359

Liu, J., Stewart, S. N., Robinson, K., Yang, Q., Lyu, W., Whitmore, M. A., & Zhang, G. (2021). Linkage between the intestinal microbiota and residual feed intake in broiler chickens. *Journal of Animal Science and Biotechnology*, *12*(1). https://doi.org/10.1186/S40104-020-00542-2

Liu, Y., Lin, Q., Huang, X., Jiang, G., Li, C., Zhang, X., Liu, S., He, L., Liu, Y., Dai, Q., & Huang, X. (2021). Effects of Dietary Ferulic Acid on the Intestinal Microbiota and the Associated Changes on the Growth Performance, Serum Cytokine Profile, and Intestinal Morphology in Ducks. *Frontiers in Microbiology*, *12*, 698213. https://doi.org/10.3389/FMICB.2021.698213

Liu, Y., Wang, Q., Liu, H., Niu, J., Jiao, N., Huang, L., Jiang, S., Guan, Q., Yang, W., & Li, Y. (2022). Effects of dietary Bopu powder supplementation on intestinal development and microbiota in broiler chickens. *Frontiers in Microbiology*, *13*. https://doi.org/10.3389/FMICB.2022.1019130

Lopera, T. J., Lujan, J. A., Zurek, E., Zapata, W., Hernandez, J. C., Toro, M. A., Alzate, J. F., Taborda, N. A., Rugeles, M. T., & Aguilar-Jimenez, W. (2021). A specific structure and high richness characterize intestinal microbiota of HIV-exposed seronegative individuals. *PLoS ONE*, *16*(12). https://doi.org/10.1371/JOURNAL.PONE.0260729

Lund, M., Bjerrum, L., & Pedersen, K. (2010). Quantification of Faecalibacterium prausnitzii- and Subdoligranulum variabile-like bacteria in the cecum of chickens by real-time PCR. *Poultry Science*, *89*(6), 1217–1224. https://doi.org/10.3382/PS.2010-00653

Mailhe, M., Ricaboni, D., Vitton, V., Cadoret, F., Fournier, P. E., & Raoult, D. (2017). ' Angelakisella massiliensis' gen. nov., sp. nov., a new bacterial species isolated from human ileum. *New Microbes and New Infections*, *16*, 51–53. https://doi.org/10.1016/J.NMNI.2017.01.003

Malykh, E. A., Butov, I. A., Ravcheeva, A. B., Krylov, A. A., Mashko, S. V., & Stoynova, N. V. (2018). Specific features of l-histidine production by Escherichia coli concerned with feedback control of AICAR formation and inorganic phosphate/metal transport. *Microbial Cell Factories*, *17*(1), 1–15. https://doi.org/10.1186/S12934-018-0890-2/FIGURES/7

May, R. M. (1972). Will a Large Complex System be Stable? *Nature 1972 238:5364*, *238*(5364), 413–414. https://doi.org/10.1038/238413a0

McKenna, A., Ijaz, U. Z., Kelly, C., Linton, M., Sloan, W. T., Green, B. D., Lavery, U., Dorrell, N., Wren, B. W., Richmond, A., Corcionivoschi, N., & Gundogdu, O. (2020). Impact of industrial production system parameters on chicken microbiomes: mechanisms to improve performance and reduce Campylobacter. *Microbiome*, *8*(1). https://doi.org/10.1186/S40168-020-00908-8

Meganathan, R. (2001). Biosynthesis of menaquinone (vitamin K2) and ubiquinone (coenzyme Q): A perspective on enzymatic mechanisms. *Vitamins and Hormones*, *61*, 173–218. https://doi.org/10.1016/s0083-6729(01)61006-9

Memon, F. U., Yang, Y., Zhang, G., Leghari, I. H., Lv, F., Wang, Y., Laghari, F., Khushk, F. A., & Si, H. (2022). Chicken Gut Microbiota Responses to Dietary Bacillus subtilis Probiotic in the Presence and Absence of Eimeria Infection. *Microorganisms*, *10*(8).

https://doi.org/10.3390/MICROORGANISMS10081548

Mesa, D., Lammel, D. R., Balsanelli, E., Sena, C., Noseda, M. D., Caron, L. F., Cruz, L. M., Pedrosa, F. O., & Souza, E. M. (2017). Cecal microbiota in broilers fed with prebiotics. *Frontiers in Genetics*, *8*(OCT), 153. https://doi.org/10.3389/FGENE.2017.00153/BIBTEX

Miao, S., Hong, Z., Jian, H., Xu, Q., Liu, Y., Wang, X., Li, Y., Dong, X., & Zou, X. (2022). Alterations in Intestinal Antioxidant and Immune Function and Cecal Microbiota of Laying Hens Fed on Coated Sodium Butyrate Supplemented Diets. *Animals : An Open Access Journal from MDPI*, *12*(5). https://doi.org/10.3390/ANI12050545

Möller, B., Oßmer, R., Howard, B. H., Gottschalk, G., & Hippe, H. (1984). Sporomusa, a new genus of gram-negative anaerobic bacteria including Sporomusa sphaeroides spec. nov. and Sporomusa ovata spec. nov. *Archives of Microbiology 1984 139:4*, *139*(4), 388–396. https://doi.org/10.1007/BF00408385

Nardella, C., Boi, D., di Salvo, M. L., Barile, A., Stetefeld, J., Tramonti, A., & Contestabile, R. (2019). Isolation of a complex formed between acinetobacter baumannii HemA and HemL, key enzymes of tetrapyrroles biosynthesis. *Frontiers in Molecular Biosciences*, *6*(FEB), 6. https://doi.org/10.3389/FMOLB.2019.00006/BIBTEX

Niku, J., Hui, F. K. C., Taskinen, S., & Warton, D. I. (2019). gllvm: Fast analysis of multivariate abundance data with generalized linear latent variable models in r. *Methods in Ecology and Evolution*, *10*(12), 2173–2182. https://doi.org/10.1111/2041-210X.13303

Nishi, N., Seki, K., Takahashi, D., & Toshima, K. (2021). Synthesis of a Pentasaccharide Repeating Unit of Lipopolysaccharide Derived from Virulent E. coli O1 and Identification of a Glycotope Candidate of Avian Pathogenic E. coli O1. *Angewandte Chemie*, *133*(4), 1817–1824. https://doi.org/10.1002/ANGE.202013729

Nygaard, P., & Smith, J. M. (1993). Evidence for a novel glycinamide ribonucleotide transformylase in Escherichia coli. *Journal of Bacteriology*, *175*(11), 3591–3597. https://doi.org/10.1128/jb.175.11.3591-3597.1993

Oakley, B. B., & Kogut, M. H. (2016). Spatial and temporal changes in the broiler chicken cecal and fecal microbiomes and correlations of bacterial taxa with cytokine gene expression. *Frontiers in Veterinary Science*, *3*(FEB), 11. https://doi.org/10.3389/FVETS.2016.00011/BIBTEX

Oakley, B. B., Lillehoj, H. S., Kogut, M. H., Kim, W. K., Maurer, J. J., Pedroso, A., Lee, M. D., Collett, S. R., Johnson, T. J., & Cox, N. A. (2014). The chicken gastrointestinal microbiome. *FEMS Microbiology Letters*, *360*(2), 100–112. https://doi.org/10.1111/1574-6968.12608

Ocejo, M., Oporto, B., & Hurtado, A. (2019). 16S rRNA amplicon sequencing characterization of caecal microbiome composition of broilers and free-range slow-growing chickens throughout their productive lifespan. *Scientific Reports 2019 9:1*, *9*(1), 1–14. https://doi.org/10.1038/s41598-019-39323-x

Oelschlaeger, P. (2021). β-Lactamases: Sequence, Structure, Function, and Inhibition. *Biomolecules*, *11*(7). https://doi.org/10.3390/BIOM11070986

Paul, S. S., Rama Rao, S. V., Hegde, N., Williams, N. J., Chatterjee, R. N., Raju, M. V. L. N., Reddy, G. N., Kumar, V., Phani Kumar, P. S., Mallick, S., & Gargi, M. (2022). Effects of Dietary Antimicrobial Growth Promoters on Performance Parameters and Abundance and Diversity of Broiler Chicken Gut Microbiome and Selection of Antibiotic Resistance Genes. *Frontiers in*

Microbiology, *13*. https://doi.org/10.3389/FMICB.2022.905050/FULL

Petzoldt, D., Breves, G., Rautenschlein, S., & Taras, D. (2016). Harryflintia acetispora gen. nov., sp. nov., isolated from chicken caecum. *International Journal of Systematic and Evolutionary Microbiology*, *66*(10), 4099–4104. https://doi.org/10.1099/IJSEM.0.001317

Piercy, D. W. T., Williams, R. B., & White, G. (1984). Evaluation of a mixture of trimethoprim and sulphaquinoxaline for the treatment of poultry: safety and palatability studies. *The Veterinary Record*, *114*(3), 60–62. https://doi.org/10.1136/VR.114.3.60

Plumbridge, J., & Vimr, E. (1999). Convergent pathways for utilization of the amino sugars N-acetylglucosamine, N-acetylmannosamine, and N-acetylneuraminic acid by Escherichia coli. *Journal of Bacteriology*, *181*(1), 47–54. https://doi.org/10.1128/JB.181.1.47-54.1999/ASSET/C11D7B3A-D542-464B-B47B-E41112B371E0/ASSETS/GRAPHIC/JB0191080002.JPEG

Polansky, O., Sekelova, Z., Faldynova, M., Sebkova, A., Sisak, F., & Rychlik, I. (2016). Important Metabolic Pathways and Biological Processes Expressed by Chicken Cecal Microbiota. *Applied and Environmental Microbiology*, *82*(5), 1569. https://doi.org/10.1128/AEM.03473-15

Porcheron, G., Chanteloup, N. K., Trotereau, A., Brée, A., & Schouler, C. (2012). Effect of Fructooligosaccharide Metabolism on Chicken Colonization by an Extra-Intestinal Pathogenic Escherichia coli Strain. *PLOS ONE*, *7*(4), e35475. https://doi.org/10.1371/JOURNAL.PONE.0035475

Rahman, M. M., Husna, A., Elshabrawy, H. A., Alam, J., Runa, N. Y., Badruzzaman, A. T. M., Banu, N. A., Al Mamun, M., Paul, B., Das, S., Rahman, M. M., Mahbub-E-Elahi, A. T. M., Khairalla, A. S., & Ashour, H. M. (2020). Isolation and molecular characterization of multidrug-resistant Escherichia coli from chicken meat. *Scientific Reports 2020 10:1*, *10*(1), 1–11. https://doi.org/10.1038/s41598-020-78367-2

Raimondi, S., Musmeci, E., Candeliere, F., Amaretti, A., & Rossi, M. (2021). Identification of mucin degraders of the human gut microbiota. *Scientific Reports 2021 11:1*, *11*(1), 1–10. https://doi.org/10.1038/s41598-021-90553-4

Reimer, L. C., Sardà Carbasse, J., Koblitz, J., Ebeling, C., Podstawka, A., & Overmann, J. (2022). BacDive in 2022: the knowledge base for standardized bacterial and archaeal data. *Nucleic Acids Research*, *50*(D1), D741. https://doi.org/10.1093/NAR/GKAB961

Reith, J., & Mayer, C. (2011). Peptidoglycan turnover and recycling in Gram-Positive bacteria. *Applied Microbiology and Biotechnology*, *92*(1), 1–11. https://doi.org/10.1007/S00253-011-3486-X/FIGURES/2

Richards-Rios, P., Fothergill, J., Bernardeau, M., & Wigley, P. (2020). Development of the Ileal Microbiota in Three Broiler Breeds. *Frontiers in Veterinary Science*, *7*, 17. https://doi.org/10.3389/FVETS.2020.00017/BIBTEX

Sahoo, C. R., Sahoo, J., Mahapatra, M., Lenka, D., Kumar Sahu, P., Dehury, B., Nath Padhy, R., & Kumar Paidesetty, S. (2021). Coumarin derivatives as promising antibacterial agent(s). *Arabian Journal of Chemistry*, *14*(2), 102922. https://doi.org/10.1016/J.ARABJC.2020.102922

Salaheen, S., Kim, S. W., Haley, B. J., Van Kessel, J. A. S., & Biswas, D. (2017). Alternative growth promoters Modulate broiler gut microbiome and enhance body weight gain. *Frontiers in Microbiology*, *8*(OCT), 2088. https://doi.org/10.3389/FMICB.2017.02088/BIBTEX

Sanches, M. S., Baptista, A. A. S., de Souza, M., Menck-Costa, M. F., Justino, L., Nishio, E. K., Oba, A., Bracarense, A. P. F. R. L., & Rocha, S. P. D. (2020). Proteus mirabilis causing cellulitis in broiler chickens. *Brazilian Journal of Microbiology*, *51*(3), 1353. https://doi.org/10.1007/S42770-020-00240-1

Sánchez-Alcoholado, L., Ordóñez, R., Otero, A., Plaza-Andrade, I., Laborda-Illanes, A., Medina, J. A., Ramos-Molina, B., Gómez-Millán, J., & Queipo-Ortuño, M. I. (2020). Gut Microbiota-Mediated Inflammation and Gut Permeability in Patients with Obesity and Colorectal Cancer. *International Journal of Molecular Sciences*, *21*(18), 1–20. https://doi.org/10.3390/IJMS21186782

Sauvage, E., Kerff, F., Terrak, M., Ayala, J. A., & Charlier, P. (2008). The penicillin-binding proteins: Structure and role in peptidoglycan biosynthesis. *FEMS Microbiology Reviews*, *32*(2), 234–258. https://doi.org/10.1111/j.1574-6976.2008.00105.x

Savin, K. W., Moate, P. J., Williams, S. R. O., Bath, C., Hemsworth, J., Wang, J., Ram, D., Zawadzki, J., Rochfort, S., & Cocks, B. G. (2022). Dietary wheat and reduced methane yield are linked to rumen microbiome changes in dairy cows. *PLOS ONE*, *17*(5), e0268157. https://doi.org/10.1371/JOURNAL.PONE.0268157

Schauer, S., Chaturvedi, S., Randau, L., Moser, J., Kitabatake, M., Lorenz, S., Verkamp, E., Schubert, W.-D., Nakayashiki, T., Murai, M., Wall, K., Thomann, H.-U., Heinz, D. W., Inokuchi, H., Söll, D., & Jahn, D. (2002). Escherichia coli Glutamyl-tRNA Reductase. *Journal of Biological Chemistry*, *277*(50), 48657–48663. https://doi.org/10.1074/jbc.m206924200

Schultheisz, H. L., Szymczyna, B. R., Scott, L. G., & Williamson, J. R. (2011). Enzymatic de Novo Pyrimidine Nucleotide Synthesis. *Journal of the American Chemical Society*, *133*(2), 297. https://doi.org/10.1021/JA1059685

Segura-Wang, M., Grabner, N., Koestelbauer, A., Klose, V., & Ghanbari, M. (2021). Genome-Resolved Metagenomics of the Chicken Gut Microbiome. *Frontiers in Microbiology*, *12*, 2390. https://doi.org/10.3389/FMICB.2021.726923/BIBTEX

Serina, L., Blondin, C., Sakamoto, H., Gilles, A. M., Bârzu, O., Krin, E., Sismeiro, O., & Danchin, A. (1995). Escherichia Coli UMP Kinase, a Member of the Aspartokinase Family, Is a Hexamer Regulated by Guanine Nucleotides and UTP. *Biochemistry*, *34*(15), 5066–5074. https://doi.org/10.1021/BI00015A018/ASSET/BI00015A018.FP.PNG_V03

Shalaby, A. M., Khattab, Y. A., & Abdel Rahman, A. M. (2006). Effects of Garlic (Alliumsativum) and chloramphenicol on growth performance, physiological parameters and survival of Nile tilapia (Oreochromis niloticus). *Journal of Venomous Animals and Toxins Including Tropical Diseases*, *12*(2), 172–201. https://doi.org/10.1590/S1678-91992006000200003

Shan, X., Goyal, A., Gregor, R., & Cordero, O. X. (2022). Annotation-free discovery of functional groups in microbial communities. *BioRxiv*, 2022.08.02.502537. https://doi.org/10.1101/2022.08.02.502537

Singh, A., Moestedt, J., Berg, A., & Schnürer, A. (2021). Microbiological Surveillance of Biogas Plants: Targeting Acetogenic Community. *Frontiers in Microbiology*, *12*, 2285. https://doi.org/10.3389/FMICB.2021.700256/BIBTEX

Snel, J., Heinen, P. P., Blok, H. J., Carman, R. J., Duncan, A. J., Allen, P. C., & Collins, M. D. (1995). Comparison of 16S rRNA sequences of segmented filamentous bacteria isolated from mice, rats,

and chickens and proposal of "Candidatus Arthromitus." *International Journal of Systematic Bacteriology*, *45*(4), 780–782. https://doi.org/10.1099/00207713-45-4-780/CITE/REFWORKS

Sprenger, G. A. (1995). Genetics of pentose-phosphate pathway enzymes of Escherichia coli K-12. *Archives of Microbiology*, *164*(5), 324–330. https://doi.org/10.1007/BF02529978

Stahl, M., & Vallance, B. A. (2019). Campylobacter. *Laboratory Models for Foodborne Infections*, 289–303. https://doi.org/10.1201/9781315120089-19

Stanley, D., Hughes, R. J., Geier, M. S., & Moore, R. J. (2016). Bacteria within the Gastrointestinal Tract Microbiota Correlated with Improved Growth and Feed Conversion: Challenges Presented for the Identification of Performance Enhancing Probiotic Bacteria. *Frontiers in Microbiology*, *7*(FEB). https://doi.org/10.3389/FMICB.2016.00187

Stanley, D., Hughes, R. J., & Moore, R. J. (2014). Microbiota of the chicken gastrointestinal tract: influence on health, productivity and disease. *Applied Microbiology and Biotechnology*, *98*(10), 4301–4310. https://doi.org/10.1007/S00253-014-5646-2

Stevenson, G., Neal, B., Liu, D., Hobbs, M., Packer, N. H., Batley, M., Redmond, J. W., Lindquist, L., & Reeves, P. (1994). Structure of the O antigen of Escherichia coli K-12 and the sequence of its rfb gene cluster. *Journal of Bacteriology*, *176*(13), 4144–4156. https://doi.org/10.1128/jb.176.13.4144-4156.1994

Su, Z., Huang, B., Mu, Q., & Wen, D. (2020). Evaluating the Potential Antibiotic Resistance Status in Environment Based on the Trait of Microbial Community. *Frontiers in Microbiology*, *11*, 2381. https://doi.org/10.3389/FMICB.2020.575707/BIBTEX

Sultana, M. F., Suzuki, M., Yamasaki, F., Kubota, W., Takahashi, K., Abo, H., & Kawashima, H. (2022). Identification of Crucial Amino Acid Residues for Antimicrobial Activity of Angiogenin 4 and Its Modulation of Gut Microbiota in Mice. *Frontiers in Microbiology*, *13*, 1914. https://doi.org/10.3389/FMICB.2022.900948/BIBTEX

Susin, A., Wang, Y., Cao, K. A. L., & Luz Calle, M. (2020). Variable selection in microbiome compositional data analysis. *NAR Genomics and Bioinformatics*, *2*(2). https://doi.org/10.1093/NARGAB/LQAA029

Tkach, S., Dorofeyev, A., Kuzenko, I., Boyko, N., Falalyeyeva, T., Boccuto, L., Scarpellini, E., Kobyliak, N., & Abenavoli, L. (2022). Current Status and Future Therapeutic Options for Fecal Microbiota Transplantation. *Medicina (Kaunas, Lithuania)*, *58*(1). https://doi.org/10.3390/MEDICINA58010084

Vallianou, N., Dalamaga, M., Stratigou, T., Karampela, I., & Tsigalou, C. (2021). Do Antibiotics Cause Obesity Through Long-term Alterations in the Gut Microbiome? A Review of Current Evidence. *Current Obesity Reports*, *10*(3), 244. https://doi.org/10.1007/S13679-021-00438-W

Van Hul, M., Le Roy, T., Prifti, E., Dao, M. C., Paquot, A., Zucker, J. D., Delzenne, N. M., Muccioli, G., Clément, K., & Cani, P. D. (2020). From correlation to causality: the case of Subdoligranulum. *Gut Microbes*, *12*(1), 1–13. https://doi.org/10.1080/19490976.2020.1849998

Wang, L., Lilburn, M., & Yu, Z. (2016). Intestinal microbiota of broiler chickens as affected by litter management regimens. *Frontiers in Microbiology*, *7*(MAY), 593. https://doi.org/10.3389/FMICB.2016.00593/BIBTEX

Wang, M., Chan, E. W. C., Yang, C., Chen, K., So, P. kin, & Chen, S. (2020). N-Acetyl-D-Glucosamine Acts as Adjuvant that Re-Sensitizes Starvation-Induced Antibiotic-Tolerant

Population of E. Coli to β-Lactam. *IScience*, *23*(11). https://doi.org/10.1016/J.ISCI.2020.101740

Wang, X., Zhou, Y. J., Wang, L., Liu, W., Liu, Y., Peng, C., & Zhao, Z. K. (2017). Engineering Escherichia coli nicotinic acid mononucleotide adenylyltransferase for fully active amidated NAD biosynthesis. *Applied and Environmental Microbiology*, *83*(13), 692–709. https://doi.org/10.1128/AEM.00692-17/SUPPL_FILE/ZAM999117928S1.PDF

Wasmund, K., Pelikan, C., Schintlmeister, A., Wagner, M., Watzka, M., Richter, A., Bhatnagar, S., Noel, A., Hubert, C. R. J., Rattei, T., Hofmann, T., Hausmann, B., Herbold, C. W., & Loy, A. (2021). Genomic insights into diverse bacterial taxa that degrade extracellular DNA in marine sediments. *Nature Microbiology*, *6*(7), 885–898. https://doi.org/10.1038/S41564-021-00917-9

Wei, X., Tao, J., Xiao, S., Jiang, S., Shang, E., Zhu, Z., Qian, D., & Duan, J. (2018). Xiexin Tang improves the symptom of type 2 diabetic rats by modulation of the gut microbiota. *Scientific Reports*, *8*(1). https://doi.org/10.1038/S41598-018-22094-2

Wetzel, S., Lachance, H., & Waldmann, H. (2010). Natural products as lead sources for drug development. *Comprehensive Natural Products II: Chemistry and Biology*, *3*, 5–46. https://doi.org/10.1016/B978-008045382-8.00058-7

Woodard, S. I., & Dailey, H. A. (1995). Regulation of Heme Biosynthesis in Escherichia coli. *Archives of Biochemistry and Biophysics*, *316*(1), 110–115. https://doi.org/10.1006/ABBI.1995.1016

Wu, Y.-T., Shen, S.-J., Liao, K.-F., & Huang, C.-Y. (2022). Dietary Plant and Animal Protein Sources Oppositely Modulate Fecal Bilophila and Lachnoclostridium in Vegetarians and Omnivores. *Microbiology Spectrum*, *10*(2). https://doi.org/10.1128/SPECTRUM.02047-21

Xiao, S. S., Mi, J. D., Mei, L., Liang, J., Feng, K. X., Wu, Y. B., Liao, X. Di, & Wang, Y. (2021). Microbial Diversity and Community Variation in the Intestines of Layer Chickens. *Animals : An Open Access Journal from MDPI*, *11*(3), 1–17. https://doi.org/10.3390/ANI11030840

Xiao, Y., Xiang, Y., Zhou, W., Chen, J., Li, K., & Yang, H. (2017). Microbial community mapping in intestinal tract of broiler chicken. *Poultry Science*, *96*(5), 1387–1393. https://doi.org/10.3382/PS/PEW372

Xu, Y., Liang, Z., Legrain, C., Rüger, H. J., & Glansdorff, N. (2000). Evolution of arginine biosynthesis in the bacterial domain: Novel gene- enzyme relationships from psychrophilic Moritella strains (Vibrionaceae) and evolutionary significance of N-α-acetyl ornithinase. *Journal of Bacteriology*, *182*(6), 1609–1615. https://doi.org/10.1128/JB.182.6.1609-1615.2000

Yang, J., Li, Y., Wen, Z., Liu, W., Meng, L., & Huang, H. (2021). Oscillospira - a candidate for the next-generation probiotics. *Gut Microbes*, *13*(1). https://doi.org/10.1080/19490976.2021.1987783

Yang, S. (2018). *otuSummary : Summarizing OTU table regarding the composition, abundance and beta diversity of abundant and rare biospheres*. https://github.com/camel315/otuSummary

Yang, S. M., Shim, G. Y., Kim, B. G., & Ahn, J. H. (2015). Biological synthesis of coumarins in Escherichia coli. *Microbial Cell Factories*, *14*(1). https://doi.org/10.1186/s12934-015-0248-y

Yang, Y., Ashworth, A. J., Willett, C., Cook, K., Upadhyay, A., Owens, P. R., Ricke, S. C., DeBruyn, J. M., & Moore, P. A. (2019). Review of Antibiotic Resistance, Ecology, Dissemination, and Mitigation in U.S. Broiler Poultry Systems. In *Frontiers in Microbiology* (Vol. 10). Frontiers Media S.A. https://doi.org/10.3389/fmicb.2019.02639

Yildirim, E., Ilina, L., Laptev, G., Filippova, V., Brazhnik, E., Dunyashev, T., Dubrovin, A., Novikova, N., Tiurina, D., Tarlavin, N., & Laishev, K. (2021). The structure and functional profile of ruminal microbiota in young and adult reindeers (Rangifer tarandus) consuming natural winter-spring and summer-autumn seasonal diets. *PeerJ*, *9*, e12389. https://doi.org/10.7717/PEERJ.12389/SUPP-2

Yonatan, Y., Amit, G., Friedman, J., & Bashan, A. (2022). Complexity–stability trade-off in empirical microbial ecosystems. *Nature Ecology & Evolution 2022 6:6*, *6*(6), 693–700. https://doi.org/10.1038/s41559-022-01745-8

Yu, Y., Lu, J., Oliphant, K., Gupta, N., Claud, K., & Lu, L. (2020). Maternal administration of probiotics promotes gut development in mouse offsprings. *PLOS ONE*, *15*(8), e0237182. https://doi.org/10.1371/JOURNAL.PONE.0237182

Zenner, C., Hitch, T. C. A., Riedel, T., Wortmann, E., Tiede, S., Buhl, E. M., Abt, B., Neuhaus, K., Velge, P., Overmann, J., Kaspers, B., & Clavel, T. (2021). Early-Life Immune System Maturation in Chickens Using a Synthetic Community of Cultured Gut Bacteria. *MSystems*, *6*(3). https://doi.org/10.1128/MSYSTEMS.01300-20

Zhang, Q., Zhang, S., Wu, S., Madsen, M. H., & Shi, S. (2022). Supplementing the early diet of broilers with soy protein concentrate can improve intestinal development and enhance short-chain fatty acid-producing microbes and short-chain fatty acids, especially butyric acid. *Journal of Animal Science and Biotechnology*, *13*(1), 1–17. https://doi.org/10.1186/S40104-022-00749-5/FIGURES/6

Zhang, X., Hu, Y., Ansari, A. R., Akhtar, M., Chen, Y., Cheng, R., Cui, L., Nafady, A. A., Elokil, A. A., Abdel-Kafy, E. S. M., & Liu, H. (2022). Caecal microbiota could effectively increase chicken growth performance by regulating fat metabolism. *Microbial Biotechnology*, *15*(3), 844. https://doi.org/10.1111/1751-7915.13841

Zhu, F. C., Lian, C. A., & He, L. S. (2020). Genomic Characterization of a Novel Tenericutes Bacterium from Deep-Sea Holothurian Intestine. *Microorganisms*, *8*(12), 1–16. https://doi.org/10.3390/MICROORGANISMS8121874

Zhu, X. Y., Zhong, T., Pandya, Y., & Joerger, R. D. (2002). 16S rRNA-based analysis of microbiota from the cecum of broiler chickens. *Applied and Environmental Microbiology*, *68*(1), 124–137. https://doi.org/10.1128/AEM.68.1.124-137.2002

Zoetendal, E. G., Plugge, C. M., Akkermans, A. D. L., & de Vos, W. M. (2003). Victivallis vadensis gen. nov., sp. nov., a sugar-fermenting anaerobe from human faeces. *International Journal of Systematic and Evolutionary Microbiology*, *53*(Pt 1), 211–215. https://doi.org/10.1099/IJS.0.02362-0

Zou, A., Nadeau, K., Xiong, X., Wang, P. W., Copeland, J. K., Lee, J. Y., Pierre, J. S., Ty, M., Taj, B., Brumell, J. H., Guttman, D. S., Sharif, S., Korver, D., & Parkinson, J. (2022). Systematic profiling of the chicken gut microbiome reveals dietary supplementation with antibiotics alters expression of multiple microbial pathways with minimal impact on community structure. *Microbiome*, *10*(1). https://doi.org/10.1186/S40168-022-01319-7

Zou, A., Sharif, S., & Parkinson, J. (2018). Lactobacillus elicits a “Marmite effect” on the chicken cecal microbiome. *NPJ Biofilms and Microbiomes*, *4*(1). https://doi.org/10.1038/S41522-018-0070-5
